# A Thiol-Mediated Three-Step Ring Expansion Cascade for the Conversion of Indoles into Functionalized Quinolines

**DOI:** 10.1021/acs.orglett.1c00205

**Published:** 2021-03-01

**Authors:** Nantachai Inprung, Michael J. James, Richard J. K. Taylor, William P. Unsworth

**Affiliations:** Department of Chemistry, University of York, Heslington, York, U.K.., YO10 5DD

## Abstract

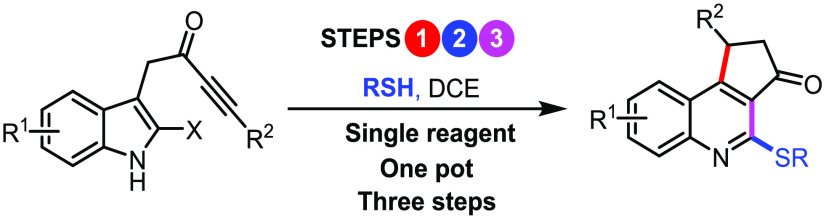

An operationally simple, high yielding three-step cascade process is described for the direct conversion of indole-tethered ynones into functionalized quinolines. A single “multitasking” thiol reagent is used to promote a three-step dearomatizing spirocyclization, nucleophilic substitution, and one-atom ring expansion reaction cascade under remarkably mild conditions. In addition, a novel route to thio-oxindoles is described, which was discovered by serendipity.

Cascade reactions (chemical processes by which two or more consecutive reactions take place in a single pot-process, also known as “tandem” or “domino” reactions) have wide utility in synthetic chemistry.^1,2^ Incorporating cascade reaction sequences into synthetic routes can significantly improve the speed and ease with which complex target molecules can be prepared and often means that the direct handling of reactive, unstable and/or toxic species can be avoided by forming these intermediates in situ.

This manuscript concerns a three-step cascade reaction sequence, starting from indole-tethered ynones **1** (Scheme 1). In recent years, ynones of this type have emerged as valuable precursors for the preparation of a diverse array of molecular scaffolds.^3−6^ For example, our groups and others have shown that the activation of the alkyne moiety of **1** promotes efficient dearomatizing spirocyclization^7,8^ to form medicinally important spirocyclic indolenines **2**;^9,10^ this is most commonly done using π-acidic catalysts (especially Ag(I) species), although Brønsted acids, palladium(II) complexes, and electrophilic halogenation reagents can also be used (**1 → 2**, Scheme 1a, step 1).^3,11,12^ Our groups have also shown that dearomatization works well on 2-halogenated indoles (i.e., **1** where X = Cl, Br or I) and that the resulting indoleninyl halide products (i.e., **2** where X = Cl, Br or I) can be transformed further via reaction with nucleophiles, or via Pd-catalyzed cross-coupling, to substitute the halide for various other groups (**2 → 3**, Scheme 1a, step 2).^5^ Finally, our groups and others have demonstrated that spirocyclic indolenines of the form **3** will rearrange via a one-atom ring expansion reaction^13^ to form annulated quinolines, with both acidic and basic reagents able to promote this transformation (**3 → 4**, Scheme 1a, step 3).^6^

**Scheme 1:**
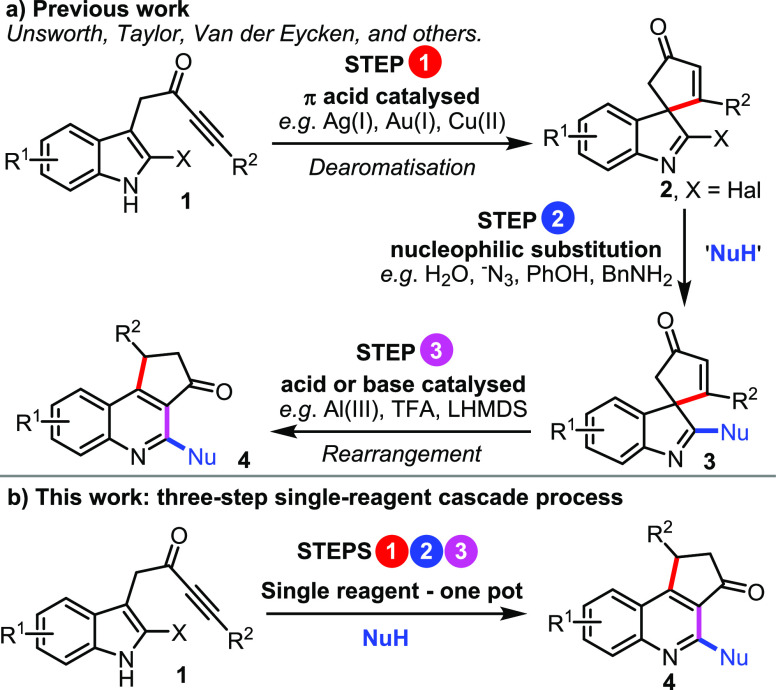
Transformations of Indole-Tethered Ynones

Efficient protocols for each of the individual steps represented in Scheme 1a are therefore established, but three steps are still required to generate functionalized quinolines **4** from ynones **1**. Quinolines are found in many marketed drugs, as well as in various other applications.^14^ On the basis of a growing understanding of each of the three individual processes discussed above,^3,5,6^ we recognized that certain reagents may be able to promote all three steps and enable the transformation of **1** into **4** via a single-cascade process (Scheme 1b); such a reagent would need to act as an acid to promote step 1, a nucleophile in step 2, and a Brønsted acid to promote step 3. The successful realization of this strategy is reported herein, with thiols emerging as the optimum “multitasking” reagent class capable of promoting the envisaged cascade, under remarkably mild and operationally simple conditions.

We started by exploring the reactivity of model 2-bromo ynone **1a**_**Br**_ with various reagents (**NuH**) that we thought might have the required acidity and nucleophilicity to promote its conversion into a quinoline of the form **4**. Phenol was tested first, and added to a solution of **1a**_**Br**_ in DCE,^15^ but no reaction was observed after stirring at RT or 60 °C (entries 1 and 2, Table 1). Next, TFA was included as an additive in the reaction, which led to the consumption of the starting material, but the only tractable products observed were oxindole **7a** (presumably formed via acid-mediated dearomatizing spirocyclization and hydrolysis of the resulting spirocycle **5a**),^5^ and bromoquinoline **8**, which likely formed via a Bronsted acid-mediated rearrangement of **5a** (*cf*. step 3).^6b^ A more acidic **NuH** reagent, 4-nitrophenol, was tested but no reaction was observed at RT (entry 4), while at 60 °C the same side products **7a** and **8** were formed (entry 5). We then decided to move on to species of similarly acidity to phenol, but also more nucleophilic, and pleasingly, thiols^16^ were found to possess this attractive combination of properties; using *n-*propanethiol, no conversion was observed at RT (entry 6), but excellent conversion into the desired quinoline **4a** was observed upon heating to 60 °C (entry 7). Furthermore, the more acidic thiophenol was able to promote the conversion of **1a**_**Br**_ into quinoline **4b** smoothly at RT (entry 8).

**Table 1:**
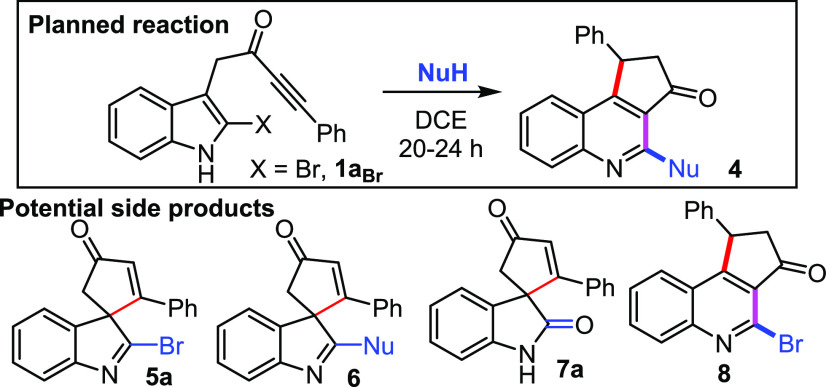
Initial Optimization^a^

entry	nucleophile (NuH)	temp	outcome^b^
1	phenol (Nu = PhO)	RT	no reaction
2	phenol (Nu = PhO)	60 °C	no reaction
3	phenol (Nu = PhO) with 1 equiv of TFA	RT	**7a** (62%) **8** (21%)
4	4-nitrophenol (Nu = 4-NO_2_C_6_H_4_O)	RT	no reaction
5	4-nitrophenol (Nu = 4-NO_2_C_6_H_4_O)	60 °C	**7a** (35%) **8** (45%)
6	*n*-propanethiol (Nu = n-PrS)	RT	no reaction
7	*n*-propanethiol (Nu = n-PrS)	60 °C	**4a** (95%)
8	thiophenol (Nu = PhS)	RT	**4b** (93%)

a **1a**_**Br**_ (1 equiv) and **NuH** (1.6 equiv) were stirred in DCE (0.1 M, degassed) for 20−24 h at the specified temperature.

b Yields are isolated material after column chromatography.

With conditions for the cascade established, attention turned to examining the reaction scope. A range of aromatic thiols were tested (Scheme 2A), and all reacted well with ynone **1a**_**Br**_; quinolines **4b**–**k** were all prepared in this manner, generally in high yield, under the standard RT conditions using a range of electronically diverse substituted thiophenols. Other aliphatic thiols were also explored, with quinolines **4a** and **4l**–**n** prepared, although in this series heating to 60 °C was required. The yield for quinoline **4n** was comparatively low (53%), with thio-oxindole **9a** also formed in 27% yield; this unexpected side reaction is discussed later in the manuscript (see Scheme 3).^17^

**Scheme 2:**
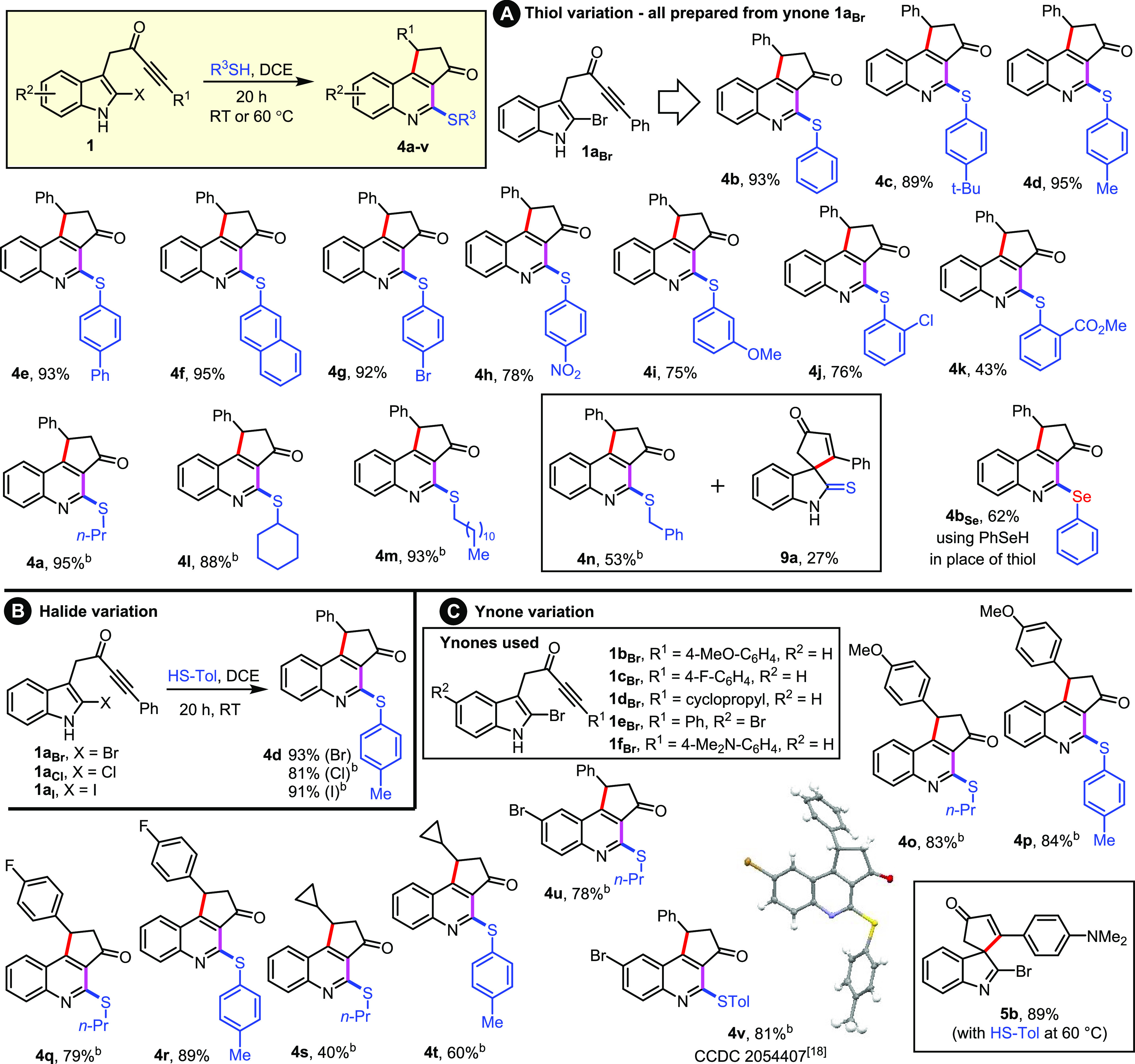
Scope of the Three-Step Thiol-Mediated Cascade for the Conversion of Ynones **1** into Quinolines **4** **1** (1 equiv) and RSH (1.6 equiv) were stirred in DCE (0.1 M) for 20 h at RT unless specified. Reaction performed at 60 °C. HS-Tol = 4-methylbenzenethiol.

**Scheme 3:**
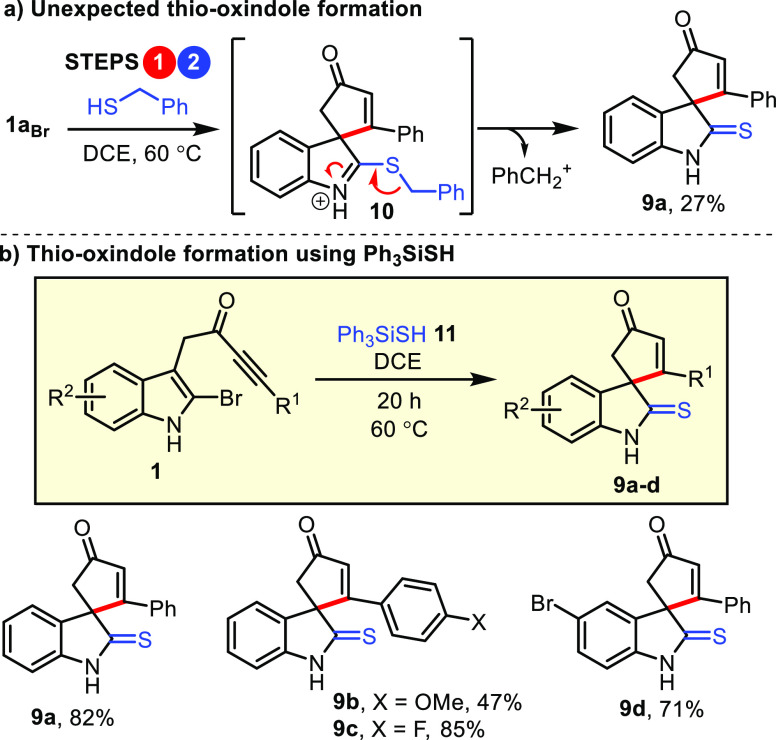
Conversion of Ynones **1** into Thio-Oxindoles **9** via a Desilylative Cascade Process **1** (1 equiv) and thiol **11** (1.6 equiv) were stirred in DCE (0.1 M) for 20 h at 60 °C.

Next, the 2-halide substituent was varied (Scheme 2B). Thus, 2-chloro (**1a**_**Cl**_) and 2-iodo (**1a**_**I**_) analogues of ynone **1a**_**Br**_ were prepared,^5^ and both reacted smoothly with 4-methylbenzenethiol to form quinoline **4d** in high yield, albeit at a higher reaction temperature (60 °C). Finally, we explored variation of the indole-tethered ynone component **1**. Four different additional 2-bromo-indole-tethered ynones were successfully tested, with variations to the ynone and the indole motifs explored. For each ynone, a representative aliphatic (*n*-propanethiol) and aromatic thiol (4-methylbenzenethiol) were tested, with the expected quinoline products **4o**–**v** to be isolated successfully in all cases.^18^ The only substrate tested that did not deliver the expected quinoline was 4-NMe_2_-substituted ynone **1f**_**Br**_; in this case, spirocyclic indoleninyl bromide **5b** was isolated in 89% yield.^19^ Despite not delivering the expected quinoline, the isolation of spirocycle **5b** does provide indirect mechanistic evidence for the intermediacy of indoleninyl halides in the reaction cascade (see later for discussion). Finally, by replacing the thiol with benzeneselenol, the analogous selenide product **4b**_**Se**_ was obtained in 62% yield.

The unexpected isolation of thio-oxindole **9a** during the synthesis of **4n** prompted additional studies, in part to better understand this side reaction, but also to try and harness it productively, as a new way to make thio-oxindoles.^20^ Our theory for how thio-oxindole **9a** formed is summarized in Scheme 3a. The reaction is likely to have started as expected, and thus it proceeded through the normal dearomatizing spirocyclization and nucleophilic substitution steps (i.e., steps 1 and 2). This would generate spirocycle **10**, and at this point, it appears that the route diverges, with some of the material going on to form quinoline **4n** in the usual way, and the rest undergoing debenzylation, either via an S_N_1-type pathway as drawn, or the analogous S_N_2-type cleavage (not shown). To test this idea and improve the yield of thio-oxindole **9a**, the reaction was repeated using the silylated thiol Ph_3_SiSH **11**; the idea was that the weak Si–S would cleave more easily than the S–Bn bond in **10**, and facilitate thio-oxindole formation via a desilylative mechanism. This idea worked well; the reaction of ynone **1a**_**Br**_ with Ph_3_SiSH **11** using the standard 60 °C procedure led to the formation of thio-oxindole **9a** in 82% isolated yield (Scheme 3b). The same procedure was applied to other 2-halo-indole-tethered ynones, with thio-oxindoles **9b**–**9d** (47–85%) prepared in the same way.

A proposed mechanism for the three-step cascade is outlined in Scheme 4a. The cascade likely initiates with dearomatizing spirocyclization, promoted by the relatively acidic thiol (**A** → **B**, step 1, Scheme 4a); protic acids have been shown to promote spirocyclization of related ynones,^3b,6b^ and the isolation of spirocyclic indoleninyl bromide **5b** discussed earlier lends further support to this notion. The resulting iminium–thiolate ion pair **2** may then undergo facile nucleophilic substitution to afford substituted spirocycle **12** (step 2).^5^ The rearrangement of **12** into **17** is then thought to proceed via a previously studied acid-catalyzed one-atom ring-expansion.^6c^

**Scheme 4:**
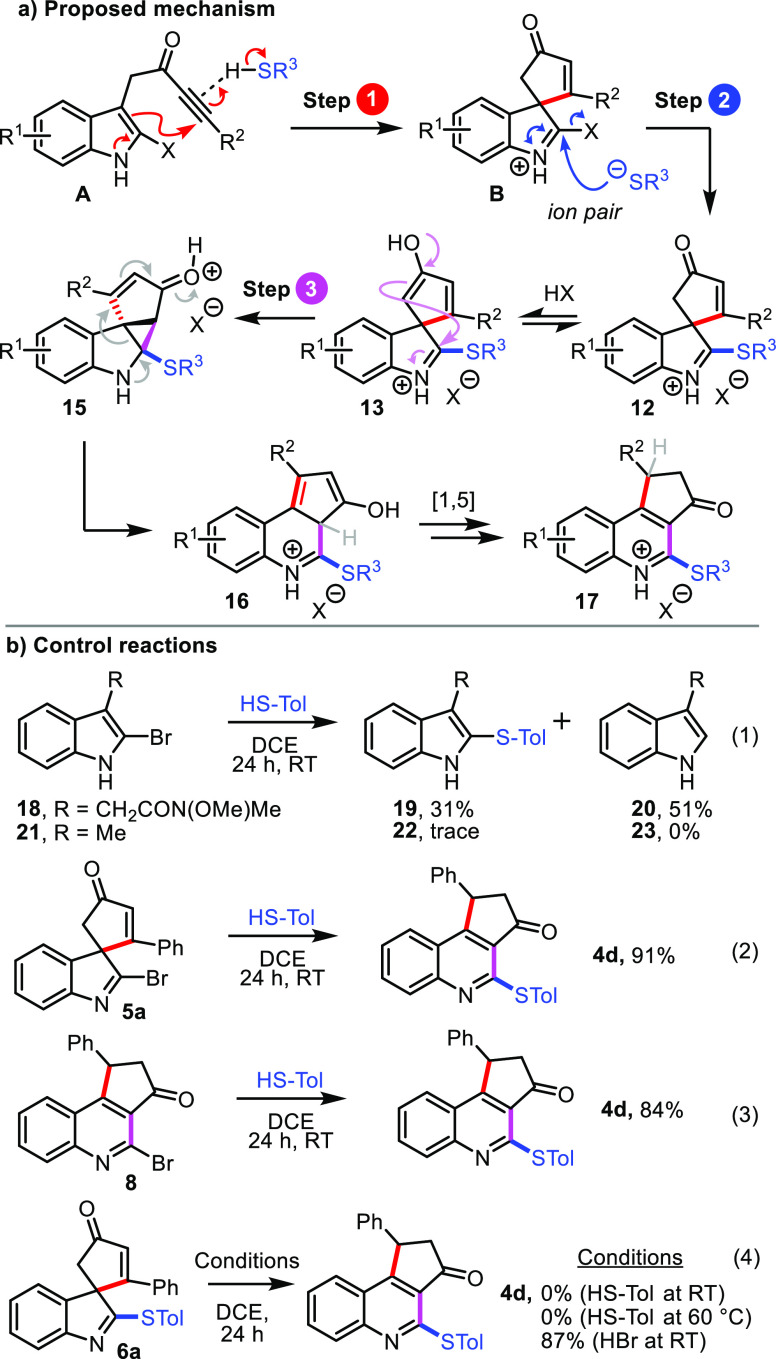
Proposed Mechanism and Control Reactions

Several control experiments were conducted to investigate this mechanism and the ordering of the steps. First, to probe whether the nucleophilic substitution step may proceed *before* spirocyclization, 2-bromo-indole substrates lacking an ynone substituent (**18** and **21**) were each reacted under the standard conditions with 4-methylbenzenethiol (Scheme 4b, eq 1). In the case of indole **18**, some bromide substitution was indeed observed, with sulfide **19** formed in 31% yield. This confirms that nucleophilic substitution directly on the indole is possible, although the yield was low, and the major product was in fact the reduced product **20**. Treating the analogous 3-methylindole **21** in the same way resulted in trace formation of **22** only. In view of these results, and given that no reduction products were observed in any of the synthetic reactions, it seems unlikely that nucleophilic substitution precedes dearomatizing spirocyclization.

We then questioned whether the iminium–thiolate ion pair **B** might first undergo ring expansion to form a quinoline and that nucleophilic substitution follows this step. To probe this idea, both indoleninyl bromide **5a** and 2-bromoquinoline **8** were reacted with 4-methylbenzenethiol under the standard reaction conditions. Interestingly, both reactions afforded the expected quinoline product **4d** in high yields (Scheme 4b, eqs 2 and 3), suggesting that the order of steps 2 and 3 could be interchanged.

To investigate this idea further, a discrete sample of the substituted spirocyclic sulfide **6a** was reacted with 4-methylbenzenethiol under the standard reaction conditions (eq 4). No conversion into quinoline **4d** was observed and only **6a** was recovered after stirring for 24 h at both RT and 60 °C. However, the quinoline product **4d** could be formed in high yield at RT upon the addition of 1.1 equiv of 48% aq. HBr to spirocyclic sulfide **6a**. This result suggests that a strong Brønsted acid is required to promote the ring expansion, and such an acid would only be present in the reaction following the nucleophilic substitution step (which generates HX), thus supporting the originally proposed order of steps. Furthermore, the success of the series of thio-oxindole forming reactions described in Scheme 3 also supports the same pathway, because in these reactions the successful formation of spirocyclic products **9a**–**d** means that nucleophilic substitution must have out-competed ring expansion in these cases.

Considering all these observations, we can be confident that the first step of the cascade is a thiol-promoted dearomatizing spirocyclization (step 1). The next step is most likely to be nucleophilic substitution (step 2) of the resultant iminium–thiolate ion pair, which generates a strong Brønsted acid (HBr) in situ. This acid then promotes a one-atom ring expansion (step 3) to form a stable aromatic quinoline product **4**. Some interchange in the ordering of steps 2 and 3 cannot be ruled out once a reasonable concentration of HBr has built up in the reaction, however.

In summary, a three-step cascade process has been developed that allows for the direct conversion of 2-halo-indole-tethered ynones into substituted quinolines. The key to the process is the use of thiols as “multitasking” reagents able to promote dearomatizing spirocyclization and nucleophilic substitution directly, as well promoting a one-atom ring expansion indirectly, via the formation of a strong Brønsted acid (HBr) in situ. The reactions are very simple to perform^21^ and are typically high yielding, enabling the facile synthesis of a diverse array of functionalized quinolines. They are also easily scalable; for example, quinoline **4d** was formed in 97% yield on a 1 mmol scale (see Supporting Information). In addition, a related route to thio-oxindoles was also developed following a serendipitous discovery of an unexpected side reaction.

## Supporting Information Available

Experimental procedures, characterization, and copies of ^1^H and ^13^C NMR spectra for all compounds (PDF)

## Accession Codes

CCDC 2054407 contains the supplementary crystallographic data for this paper. These data can be obtained free of charge via www.ccdc.cam.ac.uk/data_request/cif, or by emailing data_request@ccdc.cam.ac.uk, or by contacting The Cambridge Crystallographic Data Centre, 12 Union Road, Cambridge CB2 1EZ, UK; fax: +44 1223 336033.
