# Supplementary material for: A Thiol-Mediated Three-Step Ring Expansion Cascade for the Conversion of Indoles into Functionalized Quinolines
Source: Org Lett. 2021 Mar 1;23(6):2063–8. doi: 10.1021/acs.orglett.1c00205 (PMC8041380; doi:10.1021/acs.orglett.1c00205)
Supplement: Supplementary file 1 — ol1c00205_si_001.pdf [file ol1c00205_si_001.pdf]

Supporting Information

A thiol-mediated three-step ring expansion cascade for the conversion of indoles into functionalised quinolines

Nantachai Inprung, Michael J. James*, Richard J. K. Taylor*, William P. Unsworth*
Department of Chemistry, University of York, Heslington, York, U.K., YO10 5DD

Table of Contents

General Information 2

General Procedures 3

Experimental Procedures and Characterisation Data 4

^{1}H, ^{13}C and ^{19}F NMR Spectra 31

X-Ray Crystallography 72

References 73

General Information

All reagents were purchased from commercial sources and purification. Anhydrous CH_2Cl_2 and THF were obtained from an Innovative Technology Inc. PureSolv® solvent purification system. Anhydrous DCE was obtained from Sigma Aldrich; ^{1}H NMR, ^{13}C NMR and ^{19}F NMR spectra were recorded on a JEOL ECX400 or JEOL ECS400 spectrometer, operating at 400 MHz, 100 MHz and 376 Hz respectively. All spectral data was acquired at 295 K. Chemical shifts (δ) are quoted in parts per million (ppm). The residual solvent peak, δ_H 7.26 and δ_c 77.2 for $CDCl_3$, δ_H 3.33 and δ_c 39.5 for DMSO-d6 was used as a reference. Coupling constants (J) are reported in Hertz (Hz) to the nearest 0.5 Hz. The multiplicity abbreviations used are: s singlet, d doublet, t triplet, p pentet, sx sextet, br s broad singlet, br d broad doublet, dd doublet of doublets, ddd doublet of doublet of doublets and m multiplet. Signal assignment was achieved by analysis of DEPT, COSY, HMBC and HSQC experiments where required. Infrared (IR) spectra were recorded on a PerkinElmer UATR 2 spectrometer as a thin film dispersed from either CH_2Cl_2 or $CDCl_3$. Mass spectra (high-resolution) were obtained by the University of York Mass Spectrometry Service, using Electrospray Ionisation (ESI) on a Bruker Daltonics, Micro-tof spectrometer. Melting points were determined using Gallenkamp apparatus. Thin layer chromatography was carried out on Merck silica gel $60F_{254}$ pre-coated aluminium foil sheets and were visualised using UV light (254 nm) and stained with basic aqueous potassium permanganate. Flash column chromatography was carried out using slurry packed Fluka silica gel (SiO_2), 35–70 μm, 60 Å, under a light positive pressure, eluting with the specified solvent system.

General Procedures

General Procedure A

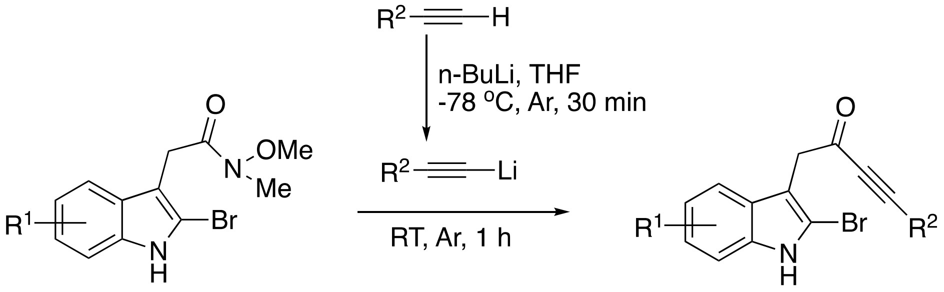

All indolyl ynones were synthesized using the following procedure, based on a literature method.[1] To a stirred solution of alkyne (2.50 mmol) in THF (3 mL) in an dried-oven round-bottom flask at −78 °C under argon was added *n*-BuLi (1.00 mL, 2.5 mmol, 2.5 M in hexanes) dropwise. The resulting solution was stirred for 30 min at −78 °C. Next, the solution was transferred via cannula into another round-bottom flask containing a solution of Weinreb amide (1.00 mmol) in dry THF (10 mL) at −78 °C. Then, the mixture was warmed to room temperature and stirred for 1 hour. After the reaction was completed, it was quenched with sat. aq. NH_4Cl (20 mL), diluted with water (30 mL) and extracted with EtOAc (3 × 50 mL). The organic layers were combined, washed with brine (50 mL), dried over $MgSO_4$, concentrated *in vacuo* and purified by flash column chromatography.

General Procedure B

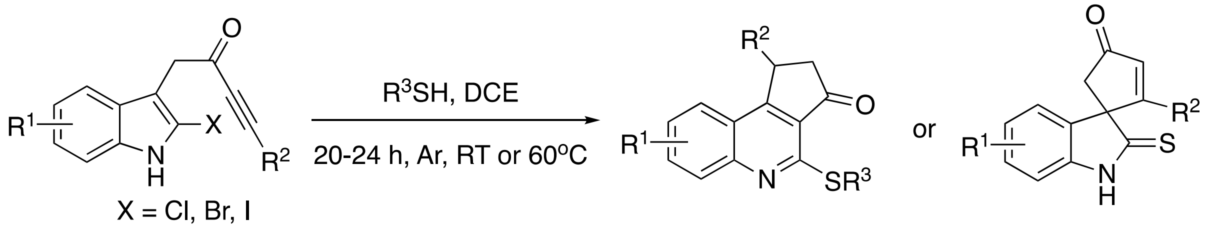

To a solution of indolyl-ynone (0.20 mmol) in DCE (2 mL, 0.1 M) in a sealed vial was added thiol (0.32 mmol). Then, the vial was degassed with argon for 5 minutes. The solution mixture was stirred for 20 – 44 hours at room temperature or 60 °C in a heating block. The reaction mixture was filtered, concentrated *in vacuo* and purified by flash column chromatography.

Experimental Procedures and Characterisation Data

Synthetic procedures and characterization data for compounds **1a$_{Br}$**, **1a$_{Cl}$**, **1a$_{I}$** were reported previously.[1]

1-(2-Bromo-1*H*-indol-3-yl)-4-(4-methoxyphenyl)but-3-yn-2-one (1b$_{Br}$)

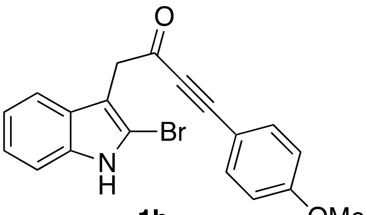

1b$_{Br}$

Synthesized using **General Procedure A** with 2-(2-bromo-1*H*-indol-3-yl)-*N*-methoxy-*N*-methylacetamide (1.35 g, 4.55 mmol), 1-ethynyl-4-methoxybenzene (1.45 mL, 11.4 mmol) and *n*-BuLi (4.55 mL, 2.5 M in hexanes) in dry THF (20 mL). Purification by flash column chromatography (hexane:EtOAc, 6:1 then 4:1 v/v) afforded the title product (1.00 g, 60%) as a yellow solid.

mp: 76 – 80 °C; **R$_f$** 0.45 (hexane:EtOAc, 2:1 v/v); **^{1}H NMR** (400 MHz, $CDCl_3$) δ 8.35 (1H, br s), 7.56 – 7.52 (1H, m), 7.31 – 7.22 (m, 3H), 7.19 – 7.14 (1H, m), 7.14 – 7.09 (1H, m), 6.80 – 6.75 (m, 2H), 3.98 (s, 2H), 3.77 (s, 3H); **^{13}C NMR** (100 MHz, $CDCl_3$) δ 184.4 (C), 161.8 (C), 136.2 (C), 135.4 (2CH), 128.0 (C), 122.8 (CH), 120.7 (CH), 118.7 (CH), 114.4 (2CH), 111.8 (C), 110.7 (CH), 110.4 (C), 108.3 (C), 93.7 (C), 87.9 (C), 55.5 (CH_3), 41.8 (CH_2); **HRMS** (ESI) m/z : $[M + Na]^+$ Calcd for $C_{19}H_{14}{}^{79}BrNNaO_2$ 390.0100; Found 390.0093, $[M + H]^+$ Calcd for $C_{19}H_{15}{}^{79}BrNO_2$ 368.0281; Found 368.0277; **ν_{max}** (thin film)/cm^{-1} 3302, 2924, 2194, 1651, 1600, 1508, 1250, 1171, 1026, 831, 735.

1-(2-Bromo-1*H*-indol-3-yl)-4-(4-fluorophenyl)but-3-yn-2-one (1c$_{Br}$)

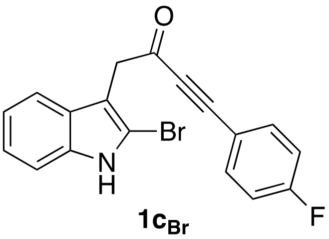

1c$_{Br}$

Synthesized using **General Procedure A** with 2-(2-bromo-1*H*-indol-3-yl)-*N*-methoxy-*N*-methylacetamide (0.90 g, 3.03 mmol), 1-ethynyl-4-fluorobenzene (0.91 g, 7.57 mmol) and *n*-BuLi (3.03 mL, 2.5 M in hexanes) in dry THF (20 mL). Purification by flash column

chromatography (hexane:EtOAc, 5:1 v/v) afforded the title product (1.00 g, 93%) as a yellow solid.

mp: 137 – 140 °C; **R_f** 0.51 (hexane:EtOAc, 2:1 v/v); **^{1}H NMR** (400 MHz, $CDCl_3$) δ 8.34 (1H, br s), 7.57 – 7.54 (1H, m), 7.34 – 7.27 (3H, m), 7.23 – 7.18 (1H, m), 7.18 – 7.12 (1H, m), 7.03 – 6.95 (2H, m), 4.02 (2H, s); **^{13}C NMR** (100 MHz, $CDCl_3$) δ 184.3 (C), 164.1 (C, d, C–F, $^1J_{C–F}$ = 254.0 Hz), 136.2 (C), 135.6 (2CH, d, C–F, $^3J_{C–F}$ = 9.0 Hz), 127.9 (C), 122.9 (CH), 120.8 (CH), 118.5 (CH), 116.2 (2CH, d, C–F, $^2J_{C–F}$ = 22.3 Hz), 116.1 (C, d, C–F, $^4J_{C–F}$ = 3.3 Hz), 110.8 (CH), 110.5 (C), 107.9 (C), 91.5 (C), 87.8 (C), 41.8 (CH_2); **^{19}F NMR** (376 MHz, $CDCl_3$) −105.85 – −105.97 (1F, m); **HRMS** (ESI) m/z: $[M + Na]^+$ Calcd for $C_{18}H_{11}{}^{79}BrFNNaO$ 377.9900; Found 377.9900, $[M + H]^+$ Calcd for $C_{18}H_{12}{}^{79}BrFNO$ 356.0081; Found 356.0079; **ν_{max}** (thin film)/cm^{-1} 3383, 3313, 2203, 1657, 1599, 1505, 1234, 837, 743.

1-(2-Bromo-1*H*-indol-3-yl)-4-cyclopropylbut-3-yn-2-one (1d$_{Br}$)

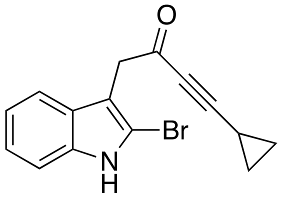

1d$_{Br}$

Synthesized using **General Procedure A** with 2-(2-bromo-1*H*-indol-3-yl)-*N*-methoxy-*N*-methylacetamide (0.90 g, 3.03 mmol), ethynylcyclopropane (0.64 mL, 7.57 mmol) and *n*-BuLi (3.03 mL, 2.5 M in hexanes) in dry THF (20 mL). Purification by flash column chromatography (hexane:EtOAc, 4:1 v/v) afforded the title product (0.87 g, 95%) as a pale yellow oil.

R_f 0.53 (hexane:EtOAc, 2:1 v/v); **^{1}H NMR** (400 MHz, $CDCl_3$) δ 8.36 (1H, br s), 7.48 (1H, br d, *J* = 7.8 Hz), 7.29 – 7.25 (m, 1H), 7.20 – 7.15 (1H, m), 7.14 – 7.09 (1H, m), 3.87 (2H, s), 1.31 – 1.20 (1H, m), 0.91 – 0.82 (2H, m), 0.71 – 0.65 (2H, m); **^{13}C NMR** (100 MHz, $CDCl_3$) δ 184.4 (C), 136.1 (C), 127.8 (C), 122.7 (CH), 120.5 (CH), 118.5 (CH), 110.7 (CH), 110.3 (C), 108.2 (C), 101.1 (C), 76.6 (C), 41.7 (CH_2), 9.9 ($2CH_2$), −0.2 (CH); **HRMS** (ESI) m/z: $[M + Na]^+$ Calcd for $C_{15}H_{12}{}^{79}BrNNaO$ 323.9994; Found 323.9992, $[M + H]^+$ Calcd for $C_{15}H_{13}{}^{79}BrNO$ 302.0175; Found 302.0175; **ν_{max}** (thin film)/cm^{-1} 3309, 2197, 1651, 1450, 1420, 1337, 1238, 946, 742.

1-(2,5-Dibromo-1*H*-indol-3-yl)-4-phenylbut-3-yn-2-one (1e$_{Br}$)

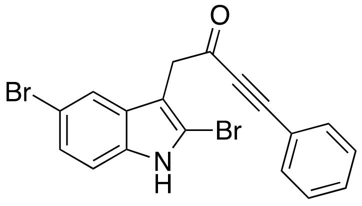

1e$_{Br}$

Synthesized using **General Procedure A** with 2-(2,5-dibromo-1*H*-indol-3-yl)-*N*-methoxy-*N*-methylacetamide (0.7702 g, 2.05 mmol), ethynylbenzene (0.56 mL, 5.12 mmol) and *n*-BuLi (2.05 mL, 2.5 M in hexanes) in dry THF (20 mL). Purification by flash column chromatography (hexane:EtOAc, 6:1 then 4:1 v/v) afforded the title product (0.76 g, 89%) as a yellow solid.

mp: 121 – 124 °C; **R$_f$** 0.55 (hexane:EtOAc, 2:1 v/v); **^{1}H NMR** (400 MHz, $CDCl_3$) δ 8.34 (1H, s), 7.71 (1H, d, *J* = 1.8 Hz), 7.46 – 7.39 (3H, m), 7.37 – 7.31 (2H, m), 7.28 (1H, dd, *J* = 8.6, 1.9 Hz), 7.17 (1H, d, *J* = 8.6 Hz), 3.99 (2H, s); **^{13}C NMR** (100 MHz, $CDCl_3$) δ 183.7 (C), 134.8 (C), 133.4 (2CH), 131.0 (CH), 129.5 (C), 128.7 (2CH), 125.8 (CH), 121.2 (CH), 119.8 (C), 114.2 (C), 112.2 (CH), 111.8 (C), 107.7 (C), 92.7 (C), 87.8 (C), 41.7 (CH_2); **HRMS** (ESI) m/z: $[M + Na]^+$ Calcd for $C_{18}H_{11}{}^{79}Br_2NNaO$ 437.9100; Found 437.9093; **ν_{max}** (thin film)/cm^{-1} 3405, 3302, 2201, 1656, 1442, 1081, 757, 687.

1-(2-Bromo-1*H*-indol-3-yl)-4-(4-(dimethylamino)phenyl)but-3-yn-2-one (1f$_{Br}$)

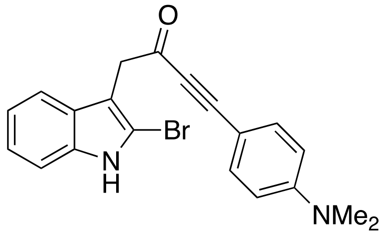

Synthesized using **General Procedure A** with 2-(2-bromo-1*H*-indol-3-yl)-*N*-methoxy-*N*-methylacetamide (0.7934 g, 2.67 mmol), 4-ethynyl-*N*,*N*-dimethylaniline (0.97 g, 6.68 mmol) and *n*-BuLi (2.67 mL, 2.5 M in hexanes) in dry THF (20 mL). Purification by flash column chromatography (hexane:EtOAc, 4:1 then 2:1 v/v) afforded the title product (0.72 g, 84%) as a yellow solid.

mp: 125 – 128 °C; R$_f$ 0.33 (hexane:EtOAc, 2:1 v/v); **^{1}H NMR** (400 MHz, $CDCl_3$) δ 8.30 (1H, br s), 7.60 – 7.56 (1H, m), 7.32 – 7.27 (1H, m), 7.23 – 7.16 (3H, m), 7.16 – 7.11 (1H, m), 6.56 – 6.51 (2H, m), 3.99 (2H, s), 2.98 (6H, s); **^{13}C NMR** (100 MHz, $CDCl_3$) δ 184.5 (C), 151.8 (C), 136.2 (C), 135.3 (CH), 133.1 (CH), 128.0 (C), 122.6 (CH), 120.6 (CH), 118.7 (CH), 111.8 (CH), 111.5 (CH), 110.7 (CH), 110.3 (C), 108.7 (C), 105.5 (C), 97.1 (C), 88.8 (C), 41.6 (CH_2),

40.1 ($2CH_3$); **HRMS** (ESI) m/z: $[M + Na]^+$ Calcd for $C_{20}H_{17}{}^{79}BrN_2NaO$ 403.0416; Found 403.0407, $[M + H]^+$ Calcd for $C_{20}H_{18}{}^{79}BrN_2O$ 381.0597; Found 381.0585; $\mathbf{\nu_{max}}$ (thin film)/cm^{-1} 3276, 2187, 2149, 1595, 1526, 1372, 1098, 817, 742.

1-Phenyl-4-(phenylthio)-1,2-dihydro-3*H*-cyclopenta[*c*]quinolin-3-one (4b)

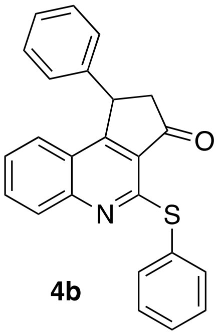

4b

Synthesized using **General Procedure B** with 1-(2-bromo-1*H*-indol-3-yl)-4-phenylbut-3-yn-2-one (67.6 mg, 0.20 mmol), thiolphenol (32.6 μL, 0.32 mmol), and DCE (2 mL, 0.1M) at RT. Purification by flash column chromatography (hexane:EtOAc, 6:1 v/v) afforded the title product (68.0 mg, 93%) as a yellow solid.

mp: 152 – 156 °C; $\mathbf{R_f}$ 0.60 (hexane:EtOAc, 2:1 v/v); $\mathbf{^1H}$ **NMR** (400 MHz, $CDCl_3$) δ 7.74 – 7.66 (3H, m), 7.63 – 7.54 (2H, m), 7.50 – 7.45 (3H, m), 7.33 – 7.22 (4H, m), 7.15 – 7.09 (2H, m), 4.97 (1H, dd, *J* = 8.1, 2.7 Hz), 3.41 (1H, dd, *J* = 19.2, 8.1 Hz), 2.77 (1H, dd, *J* = 19.2, 2.7 Hz); $\mathbf{^{13}C}$ **NMR** (100 MHz, $CDCl_3$) δ 203.5 (C), 166.9 (C), 156.9 (C), 150.6 (C), 142.7 (C), 135.9 (2CH), 132.6 (CH), 129.4 (3CH), 129.1 (CH), 129.0 (2CH), 128.7 (C), 128.0 (C), 127.5 (CH), 127.4 (2CH), 126.1 (CH), 125.5 (CH), 123.5 (C), 47.5 (CH), 43.8 (CH_2); **HRMS** (ESI) m/z: $[M + Na]^+$ Calcd for $C_{24}H_{17}NNaOS$ 390.0923; Found 390.0924, $[M + H]^+$ Calcd for $C_{24}H_{18}NOS$ 368.1104; Found 368.1105; $\mathbf{\nu_{max}}$ (thin film)/cm^{-1} 1706, 1612, 1577, 1548, 1495, 1404, 1302, 1160, 1098, 954.

Spectroscopic data matched those previously reported in the literature.[1, 2]

4-((4-(*tert*-Butyl)phenyl)thio)-1-phenyl-1,2-dihydro-3*H*-cyclopenta[*c*]quinolin-3-one (4c)

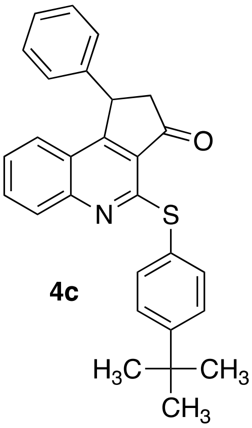

4c

Synthesized using **General Procedure B** with 1-(2-bromo-1*H*-indol-3-yl)-4-phenylbut-3-yn-2-one (67.6 mg, 0.20 mmol), 4-(*tert*-butyl)benzenethiol (55.2 μL, 0.32 mmol), and DCE (2 mL, 0.1M) at RT. Purification by flash column chromatography (hexane:EtOAc, 6:1 v/v) afforded the title product (75.0 mg, 89%) as a pale yellow oil.

$\mathbf{R_f}$ 0.40 (hexane:EtOAc, 4:1 v/v); **^{1}H NMR** (400 MHz, $CDCl_3$) δ 7.74 (1H, br d, *J* = 8.2 Hz), 7.67 – 7.62 (2H, m), 7.63 – 7.59 (1H, m), 7.58 – 7.54 (1H, m), 7.53 – 7.47 (2H, m), 7.33 – 7.21 (4H, m), 7.15 – 7.10 (2H, m), 4.96 (1H, dd, *J* = 8.0, 2.7 Hz), 3.40 (1H, dd, *J* = 19.2, 8.0 Hz), 2.77 (1H, dd, *J* = 19.2, 2.7 Hz), 1.40 (9H, s); **^{13}C NMR** (100 MHz, $CDCl_3$) 203.6 (C), 166.9 (C), 157.3 (C), 152.3 (C), 150.6 (C), 142.8 (C), 135.4 (2CH), 132.5 (CH), 129.5 (CH), 129.4 (2CH), 128.1 (C), 127.5 (3CH), 126.13 (2CH), 126.06 (CH), 125.5 (CH), 125.1 (C), 123.5 (C), 47.6 (CH_2), 43.8 (CH), 34.9 (C), 31.5 ($3CH_3$); **HRMS** (ESI) m/z: $[M + Na]^+$ Calcd $C_{28}H_{25}NNaOS$ 446.1549; Found 446.1555, $[M + H]^+$ Calcd for $C_{28}H_{26}NOS$ 424.1730; Found 424.1737; $\mathbf{\nu_{max}}$ (thin film)/cm^{-1} 2962, 1706, 1613, 1578, 1548, 1494, 1403, 1302, 1235, 954, 909. 761.

1-Phenyl-4-(*p*-tolylthio)-1,2-dihydro-3*H*-cyclopenta[*c*]quinolin-3-one (4d)

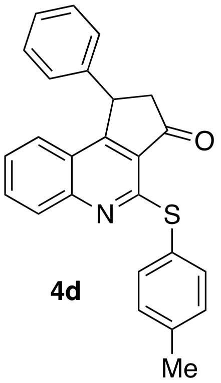

4d

Synthesized using **General Procedure B** with 1-(2-bromo-1*H*-indol-3-yl)-4-phenylbut-3-yn-2-one (67.6 mg, 0.20 mmol), 4-methylbenzenethiol (39.7 mg, 0.32 mmol) and DCE (2 mL, 0.1M) at RT. Purification by flash column chromatography (hexane:EtOAc, 6:1 v/v) afforded the title product (72.7 mg, 95%) as a pale yellow solid.

mp: 172 – 175 °C; $\mathbf{R_f}$ 0.40 (hexane:EtOAc, 4:1 v/v); **^{1}H NMR** (400 MHz, $CDCl_3$) δ 7.73 (1H, br d, *J* = 8.4 Hz), 7.63 – 7.53 (4H, m), 7.35 – 7.21 (6H, m), 7.16 – 7.09 (2H, m), 4.96 (1H, dd, *J* = 8.1, 2.7 Hz), 3.40 (1H, dd, *J* = 19.2, 8.1 Hz), 2.76 (1H, dd, *J* = 19.2, 2.7 Hz), 2.44 (3H, s); **^{13}C NMR** (100 MHz, $CDCl_3$) δ 203.6 (C), 166.8 (C), 157.3 (C), 150.6 (C), 142.8 (C), 139.2 (C), 135.8 (2CH), 132.5 (CH), 129.9 (2CH), 129.44 (CH), 129.39 (2CH), 128.1 (C), 127.5 (3CH), 126.0 (CH), 125.5 (CH), 125.0 (C), 123.4 (C), 47.6 (CH_2), 43.8 (CH), 21.6 (CH_3); **HRMS** (ESI) m/z: $[M + Na]^+$ Calcd for $C_{25}H_{19}NNaOS$ 404.1080; Found 404.1084, $[M + H]^+$ Calcd for $C_{25}H_{20}NOS$ 382.1260; Found 382.1261; $\mathbf{\nu_{max}}$ (thin film)/cm^{-1} 3060, 3025, 1709, 1615, 1579, 1549, 1493, 1404, 1305, 952, 761.

The same compound was also prepared on 1 mmol scale from 1-(2-bromo-1*H*-indol-3-yl)-4-phenylbut-3-yn-2-one (338.2 mg, 1.00 mmol), 4-methylbenzenethiol (198.7 mg, 1.60 mmol) and DCE (10 mL, 0.1M) at RT for 47 h, which afforded **4d** in 97% yield (370 mg)

4-([1,1'-Biphenyl]-4-ylthio)-1-phenyl-1,2-dihydro-3*H*-cyclopenta[*c*]quinolin-3-one (4e)

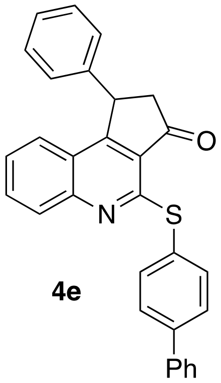

4e

Synthesized using **General Procedure B** with 1-(2-bromo-1*H*-indol-3-yl)-4-phenylbut-3-yn-2-one (67.6 mg, 0.20 mmol), [1,1'-biphenyl]-4-thiol (59.6 mg, 0.32 mmol), and DCE (2 mL, 0.1 M) at RT. Purification by flash column chromatography (hexane:EtOAc, 6:1 v/v) afforded the title product (82.3 mg, 93%) as a pale yellow solid.

mp: 105 – 107 °C; $\mathbf{R_f}$ 0.33 (hexane:EtOAc, 4:1 v/v); **^{1}H NMR** (400 MHz, $CDCl_3$) δ 7.81 – 7.77 (2H, m), 7.76 (1H, ddd, *J* = 8.5, 1.2, 0.6 Hz), 7.73 – 7.67 (4H, m), 7.63 – 7.56 (2H, m), 7.52 – 7.46 (2H, m), 7.42 – 7.37 (1H, m), 7.34 – 7.23 (m, 4H), 7.16 – 7.11 (2H, m), 4.98 (1H, dd, *J* = 8.1, 2.7 Hz), 3.41 (1H, dd, *J* = 19.2, 8.1 Hz), 2.78 (1H, dd, *J* = 19.2, 2.7 Hz); **^{13}C NMR** (100 MHz, $CDCl_3$) δ 203.5 (C), 166.9 (C), 156.9 (C), 150.6 (C), 142.7 (C), 141.9 (C), 140.6 (C), 136.1 (3CH), 132.6 (CH), 129.4 (2CH), 129.0 (2CH), 128.1 (C), 127.8 (CH), 127.7 (2CH), 127.6 (C), 127.50 (CH), 127.46 (2CH), 127.3 (2CH), 126.2 (CH), 125.6 (CH), 123.5 (C), 47.6 (CH_2), 43.8 (CH); **HRMS** (ESI) m/z: $[M + Na]^+$ Calcd for $C_{30}H_{21}NNaOS$ 466.1236; Found 466.1243, $[M + H]^+$ Calcd for $C_{30}H_{22}NOS$ 444.1417; Found 444.1420; $\mathbf{\nu_{max}}$ (thin film)/cm^{-1} 3068, 3033, 1708, 1578, 1548, 1479, 1404, 1308, 1161, 954, 760, 700.

4-(Naphthalen-2-ylthio)-1-phenyl-1,2-dihydro-3*H*-cyclopenta[*c*]quinolin-3-one (4f)

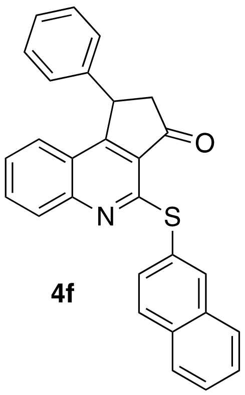

4f

Synthesized using **General Procedure B** with 1-(2-bromo-1*H*-indol-3-yl)-4-phenylbut-3-yn-2-one (67.6 mg, 0.20 mmol), 2-naphthalenethiol (51.3 mg, 0.32 mmol), and DCE (2 mL, 0.1

M) at RT. Purification by flash column chromatography (hexane:EtOAc, 6:1 v/v) afforded the title product (79.6 mg, 95%) as a yellow solid.

mp: 161 – 165 °C; **R_f** 0.34 (hexane:EtOAc, 4:1 v/v); **^{1}H NMR** (400 MHz, $CDCl_3$) δ 8.25 (1H, br s), 7.94 – 7.85 (3H, m), 7.75 (1H, dd, *J* = 8.6, 1.7 Hz), 7.67 – 7.63 (1H, m), 7.59 – 7.49 (4H, m), 7.34 – 7.22 (4H, m), 7.16 – 7.10 (2H, m), 4.98 (1H, dd, *J* = 8.1, 2.7 Hz), 3.42 (1H, dd, *J* = 19.2, 8.1 Hz), 2.79 (1H, dd, *J* = 19.2, 2.7 Hz); **^{13}C NMR** (100 MHz, $CDCl_3$) δ 203.5 (C), 166.9 (C), 157.0 (C), 150.6 (C), 142.7 (C), 134.9 (CH), 133.9 (C), 133.4 (C), 132.8 (CH), 132.6 (CH), 129.39 (2CH), 129.37 (CH), 128.13 (C), 128.11 (CH), 128.06 (C), 127.9 (CH), 127.5 (CH), 127.5 (2CH), 127.0 (CH), 126.4 (C), 126.3 (CH), 126.1 (CH), 125.5 (CH), 123.5 (C), 47.6 (CH_2), 43.8 (CH_3); **HRMS** (ESI) m/z: $[M + Na]^+$ Calcd for $C_{28}H_{19}NNaOS$ 440.1080; Found 440.1081, $[M + H]^+$ Calcd for $C_{28}H_{20}NOS$ 418.1260; Found 418.1260; **ν_{max}** (thin film)/cm^{-1} 3060, 1705, 1578, 1549, 1404, 1303, 1160, 954, 761, 730.

4-((4-Bromophenyl)thio)-1-phenyl-1,2-dihydro-3*H*-cyclopenta[*c*]quinolin-3-one (4g)

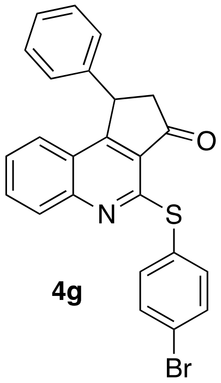

4g

Synthesized using **General Procedure B** with 1-(2-bromo-1*H*-indol-3-yl)-4-phenylbut-3-yn-2-one (67.6 mg, 0.20 mmol), 4-bromobenzene thiol (60.5 mg, 0.32 mmol), and DCE (2 mL, 0.1 M) at RT. Purification by flash column chromatography (hexane:EtOAc, 6:1 v/v) afforded the title product (82.1 mg, 92%) as a pale yellow solid.

mp: 222 – 224 °C; **R_f** 0.38 (hexane:EtOAc, 4:1 v/v); **^{1}H NMR** (400 MHz, $CDCl_3$) δ 7.74 (1H, br d, *J* = 8.4 Hz), 7.67 – 7.50 (6H, m), 7.35 – 7.22 (4H, m), 7.16 – 7.08 (2H, m), 4.98 (1H, dd, *J* = 8.1, 2.7 Hz), 3.40 (1H, dd, *J* = 19.2, 8.1 Hz), 2.77 (1H, dd, *J* = 19.2, 2.7 Hz); **^{13}C NMR** (100 MHz, $CDCl_3$) δ 203.5 (C), 167.0 (C), 156.1 (C), 150.5 (C), 142.6 (C), 137.4 (2CH), 132.7 (CH), 132.2 (2CH), 129.45 (2CH), 129.38 (CH), 128.0 (C), 127.9 (C), 127.6 (CH), 127.5 (2CH), 126.4 (CH), 125.6 (CH), 123.7 (C), 123.6 (C), 47.5 (CH_2), 43.9 (CH); **HRMS** (ESI) m/z: $[M + Na]^+$ Calcd for $C_{24}H_{16}{}^{79}BrNNaOS$ 468.0028; Found 468.0024, $[M + H]^+$ Calcd for

$C_{24}H_{17}{}^{79}BrNOS$ 446.0209; Found 446.0199; **ν_{max}** (thin film)/cm^{-1} 3064, 1708, 1611, 1578, 1548, 1473, 1404, 1160, 1098, 1010, 954, 762.

4-((4-Nitrophenyl)thio)-1-phenyl-1,2-dihydro-3*H*-cyclopenta[*c*]quinolin-3-one (4h)

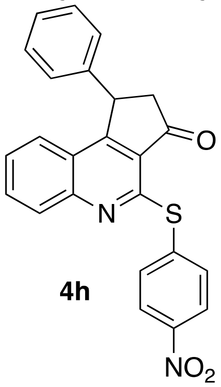

4h

Synthesized using **General Procedure B** with 1-(2-bromo-1*H*-indol-3-yl)-4-phenylbut-3-yn-2-one (67.6 mg, 0.20 mmol), 4-nitrobenzenethiol (49.7 mg, 0.32 mmol), and DCE (2 mL, 0.1 M) at RT. Purification by flash column chromatography (hexane:EtOAc, 6:1 v/v) afforded the title product (64.2 mg, 78%) as a pale yellow solid.

mp: 213 – 215 °C; **R_f** 0.56 (hexane:EtOAc, 2:1 v/v); **^{1}H NMR** (400 MHz, $CDCl_3$) δ 8.34 – 8.27 (2H, m), 7.93 – 7.87 (2H, m), 7.75 (1H, ddd, *J* = 8.5, 1.3, 0.6 Hz), 7.69 – 7.63 (1H, m), 7.61 (1H, ddd, *J* = 8.3, 1.5, 0.6 Hz), 7.36 – 7.23 (4H, m), 7.16 – 7.09 (2H, m), 5.01 (1H, dd, *J* = 8.0, 2.7 Hz), 3.43 (1H, dd, *J* = 19.3, 8.0 Hz), 2.79 (1H, dd, *J* = 19.3, 2.7 Hz); **^{13}C NMR** (100 MHz, $CDCl_3$) δ 203.4 (C), 167.3 (C), 154.5 (C), 150.4 (C), 148.0 (C), 142.4 (C), 138.2 (C), 135.8 (2CH), 133.0 (CH), 129.5 (2CH), 129.3 (CH), 128.0 (C), 127.6 (CH), 127.4 (2CH), 126.8 (CH), 125.7 (CH), 123.8 (C), 123.7 (2CH), 47.5 (CH_2), 43.9 (CH); **HRMS** (ESI) m/z: $[M + Na]^+$ Calcd for $C_{24}H_{16}N_2NaO_3S$ 435.0774; Found 435.0775, $[M + H]^+$ Calcd for $C_{24}H_{17}N_2O_3S$ 413.0954; Found 413.0956; **ν_{max}** (thin film)/cm^{-1} 3064, 3029, 1705, 1574, 1549, 1515, 1340, 1306, 953, 909, 852, 762, 729.

4-((3-Methoxyphenyl)thio)-1-phenyl-1,2-dihydro-3*H*-cyclopenta[*c*]quinolin-3-one (4i)

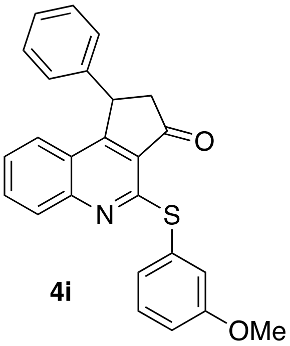

4i

Synthesized using **General Procedure B** with 1-(2-bromo-1*H*-indol-3-yl)-4-phenylbut-3-yn-2-one (67.6 mg, 0.20 mmol), 3-methoxybenzenethiol (39.7 μL, 0.32 mmol), and DCE (2 mL, 0.1 M) at RT. Purification by flash column chromatography (hexane:EtOAc, 6:1 v/v) afforded the title product (60.0 mg, 75%) as a yellow solid.

mp: 78 – 80 °C; $\mathbf{R_f}$ 0.28 (hexane:EtOAc, 4:1 v/v); ^{1}**H NMR** (400 MHz, $CDCl_3$) δ 7.74 (1H, ddd, *J* = 8.5, 1.2, 0.6 Hz), 7.63 – 7.58 (1H, m), 7.56 (1H, ddd, *J* = 8.3, 1.5, 0.6 Hz), 7.38 (1H, t, *J* = 8.2 Hz), 7.33 – 7.21 (6H, m), 7.14 – 7.09 (2H, m), 7.01 (1H, ddd, *J* = 8.2, 2.5, 1.2 Hz), 4.96 (1H, dd, *J* = 8.0, 2.7 Hz), 3.84 (3H, s), 3.39 (1H, dd, *J* = 19.2, 8.0 Hz), 2.76 (1H, dd, *J* = 19.2, 2.7 Hz); ^{13}**C NMR** (100 MHz, $CDCl_3$) δ 203.5 (C), 166.9 (C), 159.8 (C), 156.8 (C), 150.6 (C), 142.7 (C), 132.6 (CH), 129.70 (C), 129.69 (CH), 129.42 (CH), 129.39 (2CH), 128.0 (C), 127.9 (CH), 127.5 (CH), 127.4 (2CH), 126.2 (CH), 125.5 (CH), 123.5 (C), 120.6 (CH), 115.4 (CH), 55.5 (CH_3), 47.5 (CH_2), 43.8 (CH); **HRMS** (ESI) m/z: $[M + Na]^+$ Calcd for $C_{25}H_{19}NNaO_2S$ 420.1029; Found 420.1033, $[M + H]^+$ Calcd for $C_{25}H_{20}NO_2S$ 398.1209; Found 398.1214; $\boldsymbol{\nu_{max}}$ (thin film)/cm^{-1} 3064, 1707, 1613, 1577, 1549, 1478, 1404, 1304, 1283, 1233, 1041, 762.

4-((2-Chlorophenyl)thio)-1-phenyl-1,2-dihydro-3*H*-cyclopenta[*c*]quinolin-3-one (4j)

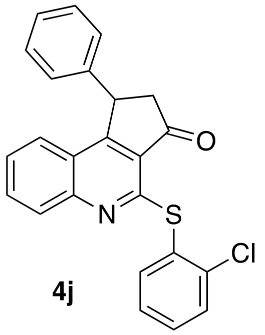

4j

Synthesized using **General Procedure B** with 1-(2-bromo-1*H*-indol-3-yl)-4-phenylbut-3-yn-2-one (67.6 mg, 0.20 mmol), 2-chlorobenzenethiol (36.3 μL, 0.32 mmol), and DCE (2 mL, 0.1 M) at RT. Purification by flash column chromatography (hexane:EtOAc, 6:1 v/v) afforded the title product (61.0 mg, 76%) as a white solid.

mp: 94 – 97 °C; **R$_f$** 0.36 (hexane:EtOAc, 4:1 v/v); **^{1}H NMR** (400 MHz, $CDCl_3$) δ 7.78 (1H, dd, *J* = 7.6, 1.7 Hz), 7.68 (1H, ddd, *J* = 8.5, 1.3, 0.6 Hz), 7.63 – 7.54 (3H, m), 7.46 – 7.40 (1H, m), 7.38 – 7.33 (1H, m), 7.33 – 7.22 (4H, m), 7.15 – 7.10 (2H, m), 4.98 (1H, dd, *J* = 8.0, 2.7 Hz), 3.41 (1H, dd, *J* = 19.2, 8.0 Hz), 2.78 (1H, dd, *J* = 19.2, 2.7 Hz); **^{13}C NMR** (100 MHz, $CDCl_3$) δ 203.5 (C), 166.9 (C), 155.3 (C), 150.6 (C), 142.7 (C), 140.5 (C), 138.1 (CH), 132.6 (CH), 130.9 (CH), 130.1 (CH), 129.5 (CH), 129.4 (2CH), 128.4 (C), 128.1 (C), 127.50 (CH), 127.48 (2CH), 127.2 (CH), 126.2 (CH), 125.6 (CH), 123.5 (C), 47.5 (CH_2), 43.9 (CH); **HRMS** (ESI) m/z: $[M + Na]^+$ Calcd for $C_{24}H_{16}{}^{35}ClNNaOS$ 424.0533; Found 424.0544, $[M + H]^+$ Calcd for $C_{24}H_{17}{}^{35}ClNOS$ 402.0714; Found 402.0723; **ν_{max}** (thin film)/cm^{-1} 3063, 1707, 1613, 1579, 1550, 1453, 1405, 1161, 1096, 954, 759, 732.

Methyl 2-((3-oxo-1-phenyl-2,3-dihydro-1*H*-cyclopenta[*c*]quinolin-4-yl)thio)benzoate (4k)

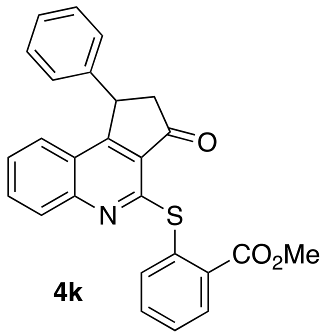

4k

Synthesized using **General Procedure B** with 1-(2-bromo-1*H*-indol-3-yl)-4-phenylbut-3-yn-2-one (67.6 mg, 0.20 mmol), methyl thiolsalicylate (44.0 μL, 0.32 mmol), and DCE (2 mL, 0.1

M) at RT. Purification by flash column chromatography (hexane:EtOAc, 5:1 v/v) afforded the title product (36.6 mg, 43%) as a yellow solid.

mp: 85 – 88 °C; **R_f** 0.20 (hexane:EtOAc, 4:1 v/v); **^{1}H NMR** (400 MHz, $CDCl_3$) δ 8.02 – 7.99 (1H, m), 7.80 – 7.73 (2H, m), 7.62 – 7.56 (1H, m), 7.58 – 7.49 (3H, m), 7.33 – 7.22 (4H, m), 7.14 – 7.09 (2H, m), 4.96 (1H, dd, *J* = 8.1, 2.7 Hz), 3.60 (3H, s), 3.40 (1H, dd, *J* = 19.2, 8.1 Hz), 2.76 (1H, dd, *J* = 19.2, 2.7 Hz); **^{13}C NMR** (100 MHz, $CDCl_3$) δ 203.2 (C), 167.7 (C), 166.9 (C), 155.7 (C), 150.3 (C), 142.7 (C), 136.4 (CH), 136.2 (C), 132.6 (CH), 131.7 (CH), 130.5 (CH), 129.4 (2CH), 129.2 (CH), 129.0 (CH), 128.1 (C), 127.50 (2CH), 127.47 (2CH), 126.2 (CH), 125.5 (CH), 123.5 (C), 52.1 (CH_3), 47.5 (CH_2), 43.9 (CH); **HRMS** (ESI) m/z: $[M + Na]^+$ Calcd for $C_{26}H_{19}NNaO_3S$ 448.0978; Found 448.0976, $[M + H]^+$ Calcd for $C_{26}H_{20}NO_3S$ 426.1158; Found 426.1158; **ν_{max}** (thin film)/cm^{-1} 3068, 2950, 1710, 1612, 1578, 1550, 1294, 1256, 1114, 1056, 955, 760.

1-Phenyl-4-(propylthio)-1,2-dihydro-3*H*-cyclopenta[*c*]quinolin-3-one (4a)

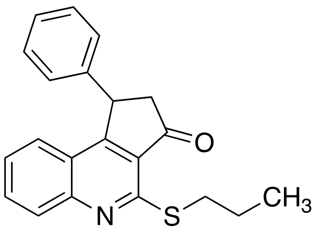

4a

Synthesized using **General Procedure B** with 1-(2-bromo-1*H*-indol-3-yl)-4-phenylbut-3-yn-2-one (67.6 mg, 0.20 mmol), 1-propanethiol (29.0 μL, 0.32 mmol), and DCE (2 mL, 0.1 M) at 60 °C. Purification by flash column chromatography (hexane:EtOAc, 8:1 v/v) afforded the title product (63.4 mg, 95%) as a pale yellow solid.

mp: 146 – 148 °C; **R_f** 0.48 (hexane:EtOAc, 4:1 v/v); **^{1}H NMR** (400 MHz, $CDCl_3$) δ 7.99 (1H, br d, *J* = 8.4 Hz), 7.71 – 7.63 (1H, m), 7.58 (1H, dd, *J* = 8.4, 1.4 Hz), 7.34 – 7.21 (4H, m), 7.14 – 7.09 (2H, m), 4.93 (1H, dd, *J* = 8.0, 2.7 Hz), 3.41 (2H, t, *J* = 7.4 Hz), 3.35 (1H, dd, *J* = 19.2, 8.0 Hz), 2.72 (1H, dd, *J* = 19.2, 2.7 Hz), 1.89 (2H, sx, *J* = 7.4 Hz), 1.15 (3H, t, *J* = 7.4 Hz); **^{13}C NMR** (100 MHz, $CDCl_3$) δ 203.6 (C), 166.6 (C), 157.7 (C), 150.7 (C), 142.9 (C), 132.6 (CH), 129.4 (2CH), 128.9 (CH), 128.5 (C), 127.5 (2CH), 127.4 (CH), 125.74 (CH), 125.70 (CH), 123.0 (C), 47.6 (CH_2), 43.6 (CH), 30.7 (CH_2), 22.5 (CH_2), 13.9 (CH_3); **HRMS** (ESI) m/z: $[M + Na]^+$ Calcd for $C_{21}H_{19}NNaOS$ 356.1080; Found 356.1072, $[M + H]^+$ Calcd for $C_{21}H_{20}NOS$ 334.1260; Found 334.1253; **ν_{max}** (thin film)/cm^{-1} 2963, 2929, 1707, 1613, 1580, 1548, 1495, 1404, 1161, 1099, 762, 701.

4-(Cyclohexylthio)-1-phenyl-1,2-dihydro-3*H*-cyclopenta[*c*]quinolin-3-one (4l)

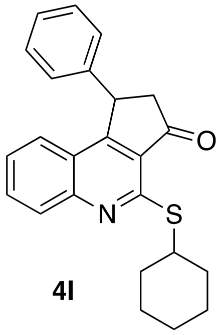

4l

Synthesized using **General Procedure B** with 1-(2-bromo-1*H*-indol-3-yl)-4-phenylbut-3-yn-2-one (67.6 mg, 0.20 mmol), cyclohexanethiol (39.1 μL, 0.32 mmol), and DCE (2 mL, 0.1 M) at 60 °C. Purification by flash column chromatography (hexane:EtOAc, 7:1 v/v) afforded the title product (65.8 mg, 88%) as a pale yellow solid.

mp: 172 – 175 °C; $\mathbf{R_f}$ 0.46 (hexane:EtOAc, 4:1 v/v); ^{1}**H NMR** (400 MHz, $CDCl_3$) δ 7.96 (1H, br d, *J* = 8.4 Hz), 7.72 – 7.65 (1H, m), 7.56 (1H, dd, *J* = 8.2, 1.4 Hz), 7.32 – 7.20 (4H, m), 7.13 – 7.06 (2H, m), 4.91 (1H, dd, *J* = 8.0, 2.7 Hz), 4.35 – 4.26 (1H, m), 3.33 (1H, dd, *J* = 19.1, 8.0 Hz), 2.70 (1H, dd, *J* = 19.1, 2.7 Hz), 2.29 – 2.15 (2H, m), 1.91 – 1.79 (2H, m), 1.74 – 1.49 (4H, m), 1.29 – 1.21 (2H, m); ^{13}**C NMR** (100 MHz, $CDCl_3$) δ 203.5 (C), 166.6 (C), 157.6 (C), 150.7 (C), 142.9 (C), 132.6 (CH), 129.5 (CH), 129.4 (2CH), 129.0 (CH), 127.5 (2CH), 127.4 (C), 125.7 (2CH), 123.0 (C), 47.6 (CH_2), 43.6 (CH), 41.2 (CH), 33.1 (CH_2), 33.0 (CH_2), 29.8 (CH_2), 26.4 (CH_2), 26.06 (CH_2); **HRMS** (ESI) m/z: $[M + Na]^+$ Calcd for $C_{24}H_{23}NNaOS$ 396.1393; Found 396.1396, $[M + H]^+$ Calcd for $C_{24}H_{24}NOS$ 374.1573; Found 374.1578; ν_{max} (thin film)/cm^{-1} 2927, 2851, 1707, 1613, 1579, 1548, 1404, 1299, 1161, 956.84, 761, 730.

4-(Dodecylthio)-1-phenyl-1,2-dihydro-3*H*-cyclopenta[*c*]quinolin-3-one (4m)

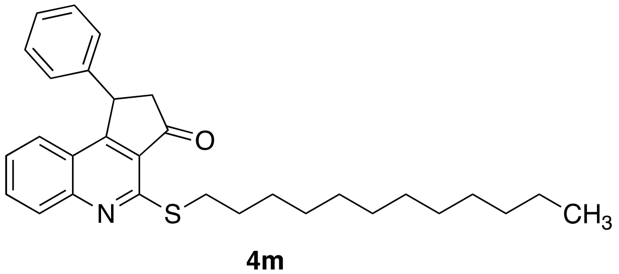

4m

Synthesized using **General Procedure B** with 1-(2-bromo-1*H*-indol-3-yl)-4-phenylbut-3-yn-2-one (67.6 mg, 0.20 mmol), 1-dodecanethiol (76.6 μL, 0.32 mmol), and DCE (2 mL, 0.1 M) at 60 °C. Purification by flash column chromatography (hexane:EtOAc, 8:1 v/v) afforded the title product (85.1 mg, 93%) as a pale yellow solid.

mp: 102 – 104 °C; **R_f** 0.64 (hexane:EtOAc, 4:1 v/v); **^{1}H NMR** (400 MHz, $CDCl_3$) δ 7.78 (1H, ddd, J = 8.5, 1.1, 0.5 Hz), 7.72 – 7.65 (1H, m), 7.57 (1H, ddd, J = 8.3, 1.5, 0.5 Hz), 7.33 – 7.19 (4H, m), 7.14 – 7.07 (2H, m), 4.92 (1H, dd, J = 8.0, 2.7 Hz), 3.41 (2H, t, J = 7.4 Hz), 3.34 (1H, dd, J = 19.1, 8.0 Hz), 2.71 (1H, dd, J = 19.1, 2.7 Hz), 1.83 (2H, p, J = 7.4 Hz), 1.54 (2H, p, J = 7.4 Hz), 1.37 – 1.21 (16H, m), 0.88 (3H, t, J = 6.9 Hz); **^{13}C NMR** (100 MHz, $CDCl_3$) δ 203.6 (C), 166.6 (C), 157.7 (C), 150.7 (C), 142.9 (C), 132.6 (CH), 129.4 (2CH), 128.9 (CH), 128.5 (C), 127.5 (2CH), 127.4 (CH), 125.7 (CH), 125.7 (CH), 123.1 (C), 47.6 (CH_2), 43.6 (CH), 32.1 (CH_2), 29.83 (CH_2), 29.79 (2CH_2), 29.7 (CH_2), 29.5 (CH_2), 29.4 (CH_2), 29.3 (CH_2), 29.1 (CH_2), 28.7 (CH_2), 22.8 (CH_2), 14.3 (CH_3); **HRMS** (ESI) m/z: $[M + Na]^+$ Calcd for $C_{30}H_{37}NNaOS$ 482.2488; Found 482.2488, $[M + H]^+$ Calcd for $C_{30}H_{38}NOS$ 460.2669; Found 460.2680; **ν_{max}** (thin film)/cm^{-1} 2922, 2852, 1709, 1613, 1580, 1547, 1404, 1300, 1098, 957, 761, 701.

4-(Benzylthio)-1-phenyl-1,2-dihydro-3*H*-cyclopenta[*c*]quinolin-3-one (4n)

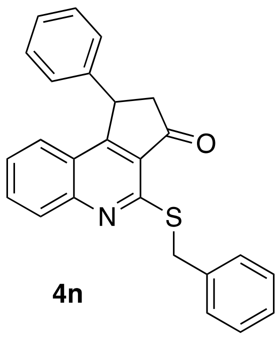

4n

Synthesized using **General Procedure B** with 1-(2-bromo-1*H*-indol-3-yl)-4-phenylbut-3-yn-2-one (67.6 mg, 0.20 mmol), benzylmercaptan (37.5 μL, 0.32 mmol), and DCE (2 mL, 0.1 M) at 60 °C. Purification by flash column chromatography (hexane:EtOAc, 6:1 v/v) afforded the title product (40.8 mg, 53%) as a pale yellow solid.

mp: 188 – 190 °C; **R_f** 0.38 (hexane:EtOAc, 4:1 v/v); **^{1}H NMR** (400 MHz, $CDCl_3$) δ 8.05 (1H, ddd, J = 8.5, 1.2, 0.6 Hz), 7.75 – 7.69 (1H, m), 7.60 – 7.53 (3H, m), 7.35 – 7.21 (7H, m), 7.13 – 7.08 (2H, m), 4.92 (1H, dd, J = 8.0, 2.7 Hz), 4.68 (2H, s), 3.33 (1H, dd, J = 19.1, 8.0 Hz), 2.70 (1H, dd, J = 19.1, 2.7 Hz); **^{13}C NMR** (100 MHz, $CDCl_3$) δ 203.4 (C), 166.7 (C), 156.8 (C), 150.6 (C), 142.7 (C), 138.2 (C), 132.7 (CH), 129.7 (2CH), 129.4 (2CH), 128.9 (CH), 128.5 (2CH), 128.4 (C), 127.5 (3CH), 127.2 (CH), 125.9 (CH), 125.8 (CH), 123.3 (C), 47.5 (CH_2), 43.8 (CH), 33.0 (CH_2); **HRMS** (ESI) m/z: $[M + Na]^+$ Calcd for $C_{25}H_{19}NNaOS$ 404.1080; Found 404.1080, $[M + H]^+$ Calcd for $C_{25}H_{20}NOS$ 382.1260; Found 382.1260; **ν_{max}** (thin film)/cm^{-1} 3060, 3028, 1707, 1612, 1579, 1548, 1495, 1404, 1161, 1098, 762.

1-Phenyl-4-(phenylselanyl)-1,2-dihydro-3*H*-cyclopenta[*c*]quinolin-3-one ($4b_{Se}$)

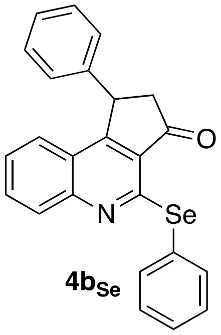

$4b_{Se}$

Synthesized using **General Procedure B** with 1-(2-bromo-1*H*-indol-3-yl)-4-phenylbut-3-yn-2-one (67.6 mg, 0.20 mmol), benzeneselenol (34.0 μL, 0.32 mmol), and DCE (2 mL, 0.1 M) at RT. Purification by flash column chromatography (hexane:EtOAc, 4:1 then 2:1 v/v) afforded the title product (51.1 mg, 62%) as a yellow solid.

mp: 161 – 163 °C; $\mathbf{R_f}$ 0.36 (hexane:EtOAc, 4:1 then 2:1 v/v); **^{1}H NMR** (400 MHz, $CDCl_3$) δ 7.82 – 7.78 (2H, m), 7.74 (1H, ddd, *J* = 8.5, 1.3, 0.7 Hz), 7.63 – 7.58 (1H, m), 7.56 (1H, ddd, *J* = 8.3, 1.6, 0.7 Hz), 7.48 – 7.42 (3H, m), 7.34 – 7.23 (4H, m), 7.14 – 7.09 (2H, m), 4.98 (1H, dd, *J* = 8.0, 2.7 Hz), 3.40 (1H, dd, *J* = 19.2, 8.0 Hz), 2.76 (1H, dd, *J* = 19.2, 2.7 Hz); **^{13}C NMR** (100 MHz, $CDCl_3$) δ 204.2 (C), 166.2 (C), 155.4 (C), 151.3 (C), 142.6 (C), 137.0 (2CH), 132.5 (CH), 129.9 (C), 129.6 (CH), 129.4 (2CH), 129.0 (2CH), 128.75 (CH), 127.49 (CH), 127.45 (2CH), 126.4 (C), 126.3 (CH), 125.6 (CH), 123.7 (C), 47.4 (CH_2), 43.9 (CH); **HRMS** (ESI) m/z: $[M + Na]^+$ Calcd for $C_{24}H_{17}NNaOSe$ 438.0368; Found 438.0372, $[M + H]^+$ Calcd for $C_{24}H_{18}NO^{80}Se$ 416.0548; Found 416.0551; $\mathbf{\nu_{max}}$ (thin film)/cm^{-1} 3061, 1704, 1613, 1574, 1548, 1404, 1303, 1157, 1092, 944, 760, 739.

1-(4-Methoxyphenyl)-4-(propylthio)-1,2-dihydro-3*H*-cyclopenta[*c*]quinolin-3-one (4o)

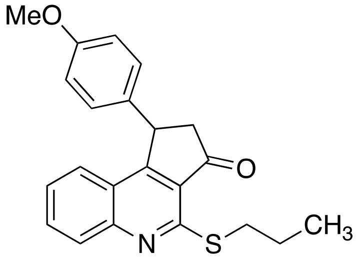

4o

Synthesized using **General Procedure B** with 1-(2-bromo-1*H*-indol-3-yl)-4-(4-methoxyphenyl) but-3-yn-2-one (73.6 mg, 0.20 mmol), propanethiol (29.0 μL, 0.32 mmol) and DCE (2 mL, 0.1 M) at 60 °C. Purification by flash column chromatography (hexane:EtOAc, 5:1 v/v) afforded the title product (60.4 mg, 83%) as a yellow solid.

mp: 128 – 130 °C; **R_f** 0.36 (hexane:EtOAc, 4:1 v/v); **^{1}H NMR** (400 MHz, $CDCl_3$) δ 7.99 (2H, br d, J = 8.5 Hz), 7.72 – 7.66 (1H, m), 7.59 (1H, ddd, J = 8.2, 1.5, 0.6 Hz), 7.30 – 7.26 (1H, m), 7.04 – 6.97 (2H, m), 6.84 – 6.78 (2H, m), 4.88 (1H, dd, J = 8.0, 2.7 Hz), 3.76 (3H, s), 3.40 (2H, t, J = 7.3 Hz), 3.32 (1H, dd, J = 19.1, 8.0 Hz), 2.67 (1H, dd, J = 19.1, 2.7 Hz), 1.87 (2H, sx, J = 7.3 Hz), 1.13 (3H, t, J = 7.3 Hz); **^{13}C NMR** (100 MHz, $CDCl_3$) δ 203.7 (C), 166.9 (C), 158.8 (C), 157.7 (C), 150.5 (C), 134.9 (C), 132.6 (CH), 128.8 (2CH), 128.5 (CH), 128.4 (C), 125.79 (CH), 125.76 (CH), 123.1 (C), 114.7 (2CH), 55.4 (CH_3), 47.7 (CH_2), 42.9 (CH), 30.7 (CH_2), 22.5 (CH_2), 13.9 (CH_3); **HRMS** (ESI) m/z: $[M + Na]^+$ Calcd for $C_{22}H_{21}NNaO_2S$ 386.1185; Found 386.1181, $[M + H]^+$ Calcd for $C_{22}H_{22}NO_2S$ 364.1366; Found 364.1365; $\mathbf{\nu_{max}}$ (thin film)/cm^{-1} 2961, 2931, 1707, 1612, 1549, 1511, 1247, 1034, 957, 765.

1-(4-Methoxyphenyl)-4-(*p*-tolylthio)-1,2-dihydro-3*H*-cyclopenta[*c*]quinolin-3-one (4p)

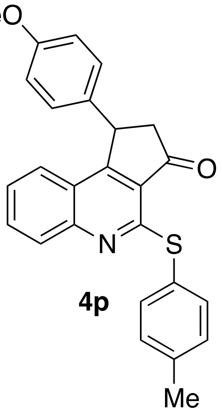

4p

Synthesized using **General Procedure B** with 1-(2-bromo-1*H*-indol-3-yl)-4-(4-methoxyphenyl) but-3-yn-2-one (73.6 mg, 0.20 mmol), 4-methylbenzenethiol (39.7 mg, 0.32 mmol) and DCE (2 mL, 0.1 M) at 60 °C. Purification by flash column chromatography (hexane:EtOAc, 6:1 v/v) afforded the title product (69.5 mg, 84%) as a pale yellow solid.

mp: 144 – 146 °C; **R_f** 0.46 (hexane:EtOAc, 4:1 v/v); **^{1}H NMR** (400 MHz, $CDCl_3$) δ 7.23 (1H, br d, J = 8.4 Hz), 7.62 – 7.54 (4H, m), 7.33 – 7.21 (3H, m), 7.07 – 6.98 (2H, m), 6.85 – 6.78 (2H, m), 4.92 (1H, dd, J = 8.0, 2.7 Hz), 3.77 (3H, s), 3.37 (1H, dd, J = 19.2, 8.0 Hz), 2.73 (1H, dd, J = 19.2, 2.7 Hz), 2.45 (3H, s); **^{13}C NMR** (100 MHz, $CDCl_3$) δ 203.8 (C), 167.1 (C), 158.8 (C), 157.3 (C), 150.6 (C), 139.2 (C), 135.8 (2CH), 134.8 (C), 132.5 (CH), 129.9 (2CH), 129.4 (CH), 128.5 (2CH), 128.0 (C), 126.0 (CH), 125.6 (CH), 125.0 (C), 123.5 (C), 114.7 (2CH), 55.4 (CH_3), 47.7 (CH_2), 43.0 (CH), 21.6 (CH_3); **HRMS** (ESI) m/z: $[M + Na]^+$ Calcd for $C_{26}H_{21}NNaO_2S$ 434.1185; Found 434.1198, $[M + H]^+$ Calcd for $C_{26}H_{22}NO_2S$ 412.1366; Found 412.1381; $\mathbf{\nu_{max}}$ (thin film)/cm^{-1} 2955, 2836, 1706, 1612, 1578, 1550, 1511, 1404, 1303, 1247, 1179, 954, 731.

1-(4-Fluorophenyl)-4-(propylthio)-1,2-dihydro-3*H*-cyclopenta[*c*]quinolin-3-one (4q)

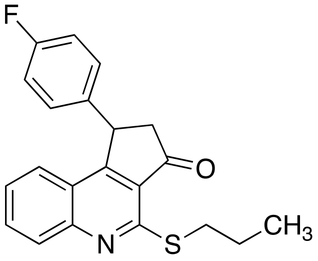

4q

Synthesized using **General Procedure B** with 1-(2-bromo-1*H*-indol-3-yl)-4-(4-fluorophenyl) but-3-yn-2-one (71.2 mg, 0.20 mmol), propanethiol (29.0 μL, 0.32 mmol) and DCE (2 mL, 0.1 M) at 60 °C. Purification by flash column chromatography (hexane:EtOAc, 6:1 v/v) afforded the title product (55.8 mg, 79%) as a pale yellow solid.

mp: 180 – 182 °C; $\mathbf{R_f}$ 0.45 (hexane:EtOAc, 4:1 v/v); **^{1}H NMR** (400 MHz, $CDCl_3$) δ 8.01 (1H, br d, *J* = 8.5 Hz), 7.73 – 7.67 (1H, m), 7.53 (1H, ddd, *J* = 8.2, 1.5, 0.6 Hz), 7.32 – 7.26 (1H, m), 7.11 – 7.03 (2H, m), 7.01 – 6.95 (2H, m), 4.92 (1H, dd, *J* = 8.1, 2.7 Hz), 3.40 (2H, t, *J* = 7.4 Hz), 3.34 (1H, dd, *J* = 19.1, 8.1 Hz), 2.66 (1H, dd, *J* = 19.1, 2.7 Hz), 1.87 (2H, sx, *J* = 7.4 Hz), 1.13 (3H, t, *J* = 7.4 Hz); **^{13}C NMR** (100 MHz, $CDCl_3$) δ 203.2 (C), 166.3 (C), 162.0 (C, d, C–F, $^1J_{C–F}$ = 246.4 Hz), 157.8 (C), 150.6 (C), 138.6 (C, d, C–F, $^4J_{C–F}$ = 3.4 Hz), 132.8 (CH), 129.0 (2CH, d, C–F , $^3J_{C–F}$ = 7.7 Hz), 128.9 (CH), 128.5 (C), 125.9 (CH), 125.6 (CH), 122.9 (C), 116.3 (2CH, d, C–F, $^2J_{C–F}$ = 21.6 Hz), 47.5 (CH_2), 42.8 (CH), 30.7 (CH_2), 22.5 (CH_2), 13.9 (CH_3); **^{19}F NMR** (376 MHz, $CDCl_3$) δ −114.62 – −114.74 (1F, m); **HRMS** (ESI) m/z: $[M + Na]^+$ Calcd for $C_{21}H_{18}FNNaOS$ 374.0985; Found 374.0975, $[M + H]^+$ Calcd for $C_{21}H_{19}FNOS$ 352.1166; Found 352.1155; $\mathbf{\nu_{max}}$ (thin film)/cm^{-1} 2963, 2926, 1708, 1580, 1548, 1508, 1403, 1225, 1160, 957, 837, 766.

1-(4-Fluorophenyl)-4-(*p*-tolylthio)-1,2-dihydro-3*H*-cyclopenta[*c*]quinolin-3-one (4r)

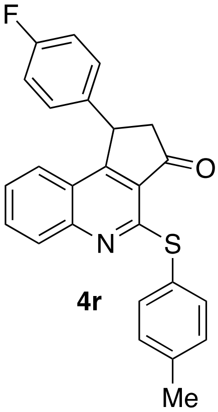

4r

Synthesized using **General Procedure B** with 1-(2-bromo-1*H*-indol-3-yl)-4-(4-fluorophenyl) but-3-yn-2-one (71.2 mg, 0.20 mmol), 4-methylbenzenethiol (39.7 mg, 0.32 mmol) and DCE (2 mL, 0.1 M) at RT. Purification by flash column chromatography (hexane:EtOAc, 6:1 then 4:1 v/v) afforded the title product (71.0 mg, 89%) as a pale yellow solid.

mp: 200 – 202 °C; $\mathbf{R_f}$ 0.38 (hexane:EtOAc, 4:1 v/v); **^{1}H NMR** (400 MHz, $CDCl_3$) δ 7.74 (1H, br d, *J* = 8.5 Hz), 7.63 – 7.55 (3H, m), 7.51 (1H, dd, *J* = 8.2, 1.4 Hz), 7.32 – 7.24 (3H, m), 7.12 – 7.05 (2H, m), 7.03 – 6.95 (2H, m), 4.96 (1H, dd, *J* = 8.1, 2.7 Hz), 3.39 (1H, dd, *J* = 19.1, 8.1 Hz), 2.71 (1H, dd, *J* = 19.1, 2.7 Hz), 2.44 (3H, s); **^{13}C NMR** (100 MHz, $CDCl_3$) δ 203.3 (C), 166.5 (C), 162.0 (C, d, C–F, $^4J_{C–F}$ = 246.6 Hz), 157.4 (C), 150.7 (C), 139.3 (C), 138.5 (C, d, C–F, $^4J_{C–F}$ = 3.3 Hz), 135.8 (2CH), 132.6 (CH), 129.9 (2CH), 129.5 (CH), 129.0 (2CH, d, C–F, $^3J_{C–F}$ = 8.1 Hz), 128.0 (C), 126.1 (CH), 125.4 (CH), 124.9 (C), 123.3 (C), 116.4 (2CH, d, C–F, $^2J_{C–F}$ = 21.6 Hz), 47.5 (CH_2), 43.0 (CH), 21.6 (CH_3); **^{19}F NMR** (376 MHz, $CDCl_3$) δ −114.55 – −114.67 (1F, m); **HRMS** (ESI) m/z: $[M + Na]^+$ Calcd for $C_{25}H_{18}FNNaOS$ 422.0985; Found 422.0998, $[M + H]^+$ Calcd for $C_{25}H_{19}FNOS$ 400.1166; Found 400.1174; $\mathbf{\nu_{max}}$ (thin film)/cm^{-1} 3073, 2918, 1708, 1580, 1550, 1509, 1404, 1160, 954, 838.

1-Cyclopropyl-4-(propylthio)-1,2-dihydro-3*H*-cyclopenta[*c*]quinolin-3-one (4s)

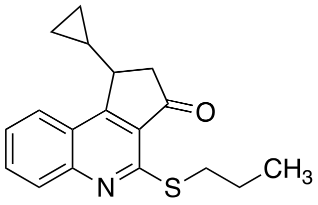

4s

Synthesized using **General Procedure B** with 1-(2-bromo-1*H*-indol-3-yl)-4-cyclopropylbut-3-yn-2-one (60.4 mg, 0.20 mmol), propanethiol (29.0 μL, 0.32 mmol) and DCE (2 mL, 0.1 M)

at 60 °C. Purification by flash column chromatography (hexane:EtOAc, 8:1 v/v) afforded the title product (23.6 mg, 40%) as a yellow solid.

mp: 99 – 102 °C; **R_f** 0.53 (hexane:EtOAc, 4:1 v/v); **^{1}H NMR** (400 MHz, $CDCl_3$) δ 8.20 (1H, ddd, *J* = 8.2, 1.5, 0.6 Hz), 8.01 (1H, ddd, *J* = 8.5, 1.3, 0.6 Hz), 7.81 – 7.75 (1H, m), 7.54 – 7.48 (1H, m), 3.46 – 3.40 (1H, m), 3.36 (2H, t, *J* = 7.3 Hz), 2.93 (1H, dd, *J* = 18.8, 7.5 Hz), 2.63 (1H, dd, *J* = 18.8, 2.0 Hz), 1.83 (2H, sx, *J* = 7.3 Hz), 1.16 – 1.01 (4H, m), 0.82 – 0.74 (1H, m), 0.68 – 0.61 (1H, m), 0.61 – 0.53 (1H, m), 0.37 – 0.30 (m, 1H); **^{13}C NMR** (100 MHz, $CDCl_3$) δ 203.9 (C), 168.2 (C), 157.8 (C), 150.4 (C), 132.6 (CH), 129.0 (CH), 127.5 (C), 126.0 (CH), 125.6 (CH), 123.5 (C), 43.5 (CH_2), 41.0 (CH), 30.6 (CH_2), 22.5 (CH_2), 17.3 (CH), 13.9 (CH_3), 7.5 (CH_2), 4.1 (CH_2); **HRMS** (ESI) m/z: $[M + Na]^+$ Calcd for $C_{18}H_{19}NNaOS$ 320.1080; Found 320.1078, $[M + H]^+$ Calcd for $C_{18}H_{20}NOS$ 298.1260 ; Found 298.1260; **ν_{max}** (thin film)/cm^{-1} 2963, 1706, 1612, 1578, 1548, 1405, 1160, 1100, 762.

1-Cyclopropyl-4-(*p*-tolylthio)-1,2-dihydro-3*H*-cyclopenta[*c*]quinolin-3-one (4t)

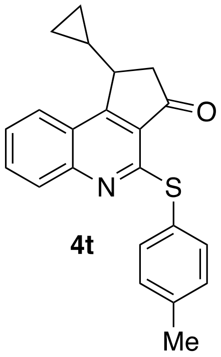

4t

Synthesized using **General Procedure B** with 1-(2-bromo-1*H*-indol-3-yl)-4-cyclopropylbut-3-yn-2-one (60.4 mg, 0.20 mmol), 4-methylbenzenethiol (39.7 mg, 0.32 mmol) and DCE (2 mL, 0.1 M) at 60 °C. Purification by flash column chromatography (hexane:EtOAc, 6:1 v/v) afforded the title product (41.8 mg, 60%) as a yellow solid.

mp: 99 – 101 °C; **R_f** 0.39 (hexane:EtOAc, 4:1 v/v); **^{1}H NMR** (400 MHz, $CDCl_3$) δ 8.20 (1H, ddd, *J* = 8.2, 1.5, 0.6 Hz), 7.76 (1H, ddd, *J* = 8.5, 1.4, 0.6 Hz), 7.72 – 7.65 (1H, m), 7.57 – 7.53 (2H, m), 7.52 – 7.46 (1H, m), 7.31 – 7.21 (m, 2H), 3.51 – 3.45 (1H, m), 2.99 (1H, dd, *J* = 18.8, 7.6 Hz), 2.69 (1H, dd, *J* = 18.8, 2.0 Hz), 2.43 (3H, s), 1.14 – 1.03 (1H, m), 0.84 – 0.75 (1H, m), 0.72 – 0.54 (m, 2H), 0.39 – 0.31 (1H, m); **^{13}C NMR** (100 MHz, $CDCl_3$) δ 203.9 (C), 168.5 (C), 157.5 (C), 150.5 (C), 139.1 (C), 135.7 (2CH), 132.5 (CH), 129.8 (2CH), 129.5 (CH), 127.1 (C), 125.9 (CH), 125.8 (CH), 125.2 (C), 123.9 (C), 43.6 (CH_2), 41.2 (CH), 21.6 (CH_3), 17.3 (CH), 7.5 (CH_2), 4.1 (CH_2); **HRMS** (ESI) m/z: $[M + Na]^+$ Calcd for $C_{22}H_{19}NNaOS$ 368.1080;

Found 368.1080, [M + H]$^+$ Calcd for $C_{22}H_{20}NOS$ 346.1260; Found 346.1257; **ν$_{max}$** (thin film)/cm^{-1} 1706, 1612, 1576, 1550, 1493, 1406, 1302, 1159, 1091, 765.

8-Bromo-1-phenyl-4-(propylthio)-1,2-dihydro-3*H*-cyclopenta[*c*]quinolin-3-one (4u)

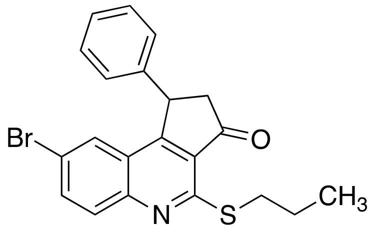

4u

Synthesized using **General Procedure B** with 1-(2,5-dibromo-1H-indol-3-yl)-4-phenylbut-3-yn-2-one (83.4 mg, 0.20 mmol), propanethiol (29.0 μL, 0.32 mmol) and DCE (2 mL, 0.1 M) at 60 °C. Purification by flash column chromatography (hexane:EtOAc, 6:1 v/v) afforded the title product (64.7 mg, 78%) as a yellow solid.

mp: 165 – 169 °C; **R$_f$** 0.54 (hexane:EtOAc, 4:1 v/v); **^{1}H NMR** (400 MHz, $CDCl_3$) δ 7.83 (1H, d, *J* = 8.9 Hz), 7.73 (1H, dd, *J* = 8.9, 2.2 Hz), 7.67 (1H, d, *J* = 2.2 Hz), 7.34 – 7.23 (3H, m), 7.12 – 7.06 (2H, m), 4.86 (1H, dd, *J* = 8.0, 2.6 Hz), 3.39 – 3.30 (3H, m), 3.35 (1H, dd, *J* = 19.2, 8.0 Hz), 2.72 (dd, *J* = 19.2, 2.6 Hz, 1H), 1.85 (2H, sx, *J* = 7.4 Hz), 1.12 (3H, t, *J* = 7.4 Hz); **^{13}C NMR** (100 MHz, $CDCl_3$) δ 203.3 (C), 165.4 (C), 158.4 (C), 149.2 (C), 142.1 (C), 135.8 (CH), 130.5 (CH), 129.5 (2CH), 128.9 (C), 127.8 (CH), 127.7 (CH), 127.4 (2CH), 124.3 (C), 119.3 (C), 47.5 (CH_2), 43.5 (CH), 30.8 (CH_2), 22.4 (CH_2), 13.9 (CH_3); **HRMS** (ESI) m/z: [M + Na]$^+$ Calcd for $C_{21}H_{18}{}^{79}BrNNaOS$ 434.0185 ;Found 434.0194; **ν$_{max}$** (thin film)/cm^{-1} 2964, 2927, 1709, 1577, 1542, 1386, 1066, 936, 830, 701.

8-Bromo-1-phenyl-4-(*p*-tolylthio)-1,2-dihydro-3*H*-cyclopenta[*c*]quinolin-3-one (4v)

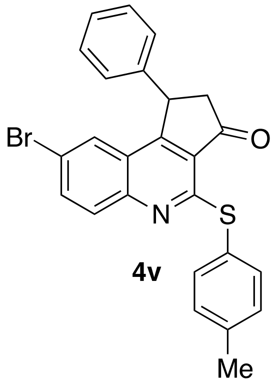

Synthesized using **General Procedure B** with 1-(2,5-dibromo-1H-indol-3-yl)-4-phenylbut-3-yn-2-one (83.4 mg, 0.20 mmol), 4-methylbenzenethiol (39.7 mg, 0.32 mmol) and DCE (2 mL, 0.1 M) at 60 °C. Purification by flash column chromatography (hexane:EtOAc, 6:1 v/v) afforded the title product (74.2 mg, 81%) as a yellow solid.

mp: 170 – 172 °C; **R_f** 0.45 (hexane:EtOAc, 4:1 v/v); **^{1}H NMR** (400 MHz, $CDCl_3$) δ 7.67 (1H, dd, *J* = 2.2, 0.6 Hz), 7.63 (1H, dd, *J* = 8.9, 2.2 Hz), 7.59 – 7.54 (3H, m), 7.36 – 7.24 (5H, m), 7.14 – 7.09 (2H, m), 4.90 (1H, dd, *J* = 8.1, 2.7 Hz), 3.40 (1H, dd, *J* = 19.2, 8.1 Hz), 2.77 (1H, dd, *J* = 19.2, 2.7 Hz), 2.44 (3H, s); **^{13}C NMR** (100 MHz, $CDCl_3$) δ 203.3 (C), 165.6 (C), 158.1 (C), 149.2 (C), 142.1 (C), 139.4 (C), 135.8 (2CH), 135.6 (CH), 131.0 (CH), 129.9 (2CH), 129.6 (2CH), 128.5 (C), 127.8 (CH), 127.6 (CH), 127.4 (2CH), 124.7 (C), 124.6 (C), 119.7 (C), 47.5 (CH_2), 43.7 (CH), 21.6 (CH_3); **HRMS** (ESI) m/z: $[M + Na]^+$ Calcd for $C_{25}H_{18}{}^{79}BrNNaOS$ 482.0185; Found 482.0186, $[M + H]^+$ Calcd for $C_{25}H_{19}{}^{79}BrNOS$ 460.0365; Found 460.0368; **ν_{max}** (thin film)/cm^{-1} 3027, 2916, 1711, 1575, 1544, 1490, 1387, 1066, 962, 829, 702.

2'-Bromo-2-(4-(dimethylamino)phenyl)spiro[cyclopentane-1,3'-indol]-2-en-4-one (5b)

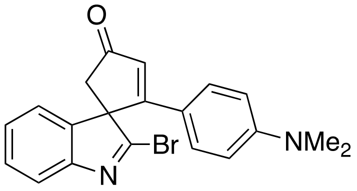

Synthesized using general method B with 1-(2-bromo-1*H*-indol-3-yl)-4-(4-(dimethylamino) phenyl)but-3-yn-2-one (76.3 mg, 0.20 mmol), 4-methylbenzenethiol (39.7 mg, 0.32 mmol) and DCE (2 mL, 0.1 M) at 60 °C. Purification by flash column chromatography (hexane:EtOAc, 4:1 then 2:1 v/v) afforded the title product (68.0 mg, 89%) as a yellow solid.

mp: 195 – 197 °C; **R_f** 0.48 (hexane:EtOAc, 1:1 v/v); **^{1}H NMR** (400 MHz, $CDCl_3$) δ 7.70 – 7.67 (1H, m), 7.41 (ddd, *J* = 7.8, 7.2, 1.6 Hz, 1H), 7.27 – 7.22 (1H, m), 7.21 (ddd, *J* = 7.4, 1.6,

0.7 Hz, 1H), 6.95 – 6.87 (2H, m), 6.75 (1H, s), 6.45 (2H, br d, *J* = 8.8 Hz), 2.95 (1H, d, *J* = 18.3 Hz), 2.94 (6H, s), 2.62 (1H, d, *J* = 18.3 Hz); **^{13}C NMR** (100 MHz, $CDCl_3$) δ 203.3 (C), 170.2 (C), 167.2 (C), 153.5 (C), 152.3 (C), 142.7 (C), 129.2 (CH), 129.0 (2CH), 127.6 (CH), 126.5 (CH), 122.0 (CH), 121.3 (CH), 118.8 (C), 111.7 (2CH), 69.5 (C), 45.4 (CH_2), 40.0 ($2CH_3$); **HRMS** (ESI) m/z: $[M + Na]^+$ Calcd for $C_{20}H_{17}BrN_2NaO$ 403.0416; Found 403.0424, $[M + H]^+$ Calcd for $C_{20}H_{18}BrN_2O$ 381.0597; Found 381.0601; $\mathbf{\nu_{max}}$ (thin film)/cm^{-1} 2909, 1687, 1606, 1570, 1522, 1202, 1171, 946, 819, 731.

2-Phenyl-2'-thioxospiro[cyclopentane-1,3'-indolin]-2-en-4-one (9a)

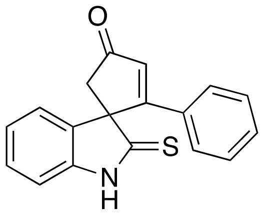

9a

Synthesized using **General Procedure B** with 1-(2-bromo-1H-indol-3-yl)-4-phenylbut-3-yn-2-one (67.6 mg, 0.20 mmol), triphenylsilanethiol (93.6 mg, 0.32 mmol), and DCE (2 mL, 0.1 M) at 60 °C. Purification by flash column chromatography (hexane:EtOAc, 4:1 then 2:1 v/v) afforded the title product (47.9 mg, 82%) as a yellow solid.

mp: 200 – 202 °C ; $\mathbf{R_f}$ 0.31 (hexane:EtOAc, 2:1 v/v); **^{1}H NMR** (400 MHz, $CDCl_3$) δ 10.42 (1H, br s), 7.34 (1H, ddd, *J* = 7.8, 6.3, 2.5 Hz), 7.31 – 7.25 (1H, m), 7.22 – 7.13 (4H, m), 7.11 – 7.04 (3H, m), 6.82 (1H, s), 3.18 (1H, d, *J* = 18.0 Hz), 2.81 (1H, d, *J* = 18.0 Hz); **^{13}C NMR** (100 MHz, $CDCl_3$) δ 207.8 (C), 205.6 (C), 172.7 (C), 142.4 (C), 136.1 (C), 132.6 (C), 131.6 (CH), 131.0 (CH), 129.5 (CH), 128.9 (2CH), 127.6 (2CH), 125.2 (CH), 123.7 (CH), 110.8 (CH), 68.1 (C), 53.1 (CH_2); **HRMS** (ESI) m/z: $[M + Na]^+$ Calcd for $C_{18}H_{13}NNaOS$ 314.0610 ; Found 314.0615, $[M + H]^+$ Calcd for $C_{18}H_{14}NOS$ 292.0791; Found 292.0796; $\mathbf{\nu_{max}}$ (thin film)/cm^{-1} 3173, 3056, 1685, 1465, 1439, 1345, 1270, 1227, 1206, 965, 753, 728.

Spiro thio-oxindole **9a** was also synthesized using **General Procedure B** with 1-(2-bromo-1H-indol-3-yl)-4-phenylbut-3-yn-2-one (67.6 mg, 0.20 mmol), benzylmercaptan (37.5 μL, 0.32 mmol) and DCE (2 mL, 0.1M) at 60 °C for 24 hours. The crude product was purified by column chromatography (hexane:EtOAc, 6:1 then 2:1 v/v) to afford the title product as a yellow solid (15.7 mg, 27%).

2-(4-Methoxyphenyl)-2'-thioxospiro[cyclopentane-1,3'-indolin]-2-en-4-one (9b)

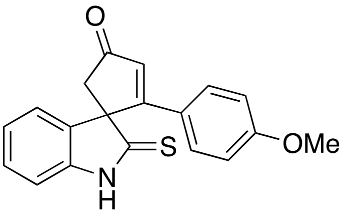

9b

Synthesized using **General Procedure B** with 1-(2-bromo-1*H*-indol-3-yl)-4-(4-methoxyphenyl) but-3-yn-2-one (73.6 mg, 0.20 mmol), triphenylsilanethiol (93.6 mg, 0.32 mmol) and DCE (2 mL, 0.1 M) at 60 °C. Purification by flash column chromatography (hexane:EtOAc, 3:1 then 2:1 v/v) afforded the title product (30.0 mg, 47%) as a brown oil.

$\mathbf{R_f}$ 0.25 (hexane:EtOAc, 2:1 v/v); ^{1}**H NMR** (400 MHz, $CDCl_3$) δ 10.89 (1H, br s), 7.33 (1H, ddd, *J* = 7.7, 5.8, 3.1 Hz), 7.16 – 7.09 (3H, m), 7.08 – 7.02 (2H, m), 6.82 (1H, s), 6.72 – 6.67 (2H, m), 3.69 (3H, s), 3.15 (1H, d, *J* = 18.0 Hz), 2.79 (1H, d, *J* = 18.0 Hz); ^{13}**C NMR** (100 MHz, $CDCl_3$) δ 208.2 (C), 205.6 (C), 172.1 (C), 161.9 (C), 142.3 (C), 136.6 (C), 129.5 (2CH), 129.4 (2CH), 125.2 (CH), 124.8 (C), 123.6 (CH), 114.4 (2CH), 111.0 (CH), 67.9 (C), 55.4 (CH_3), 53.3 (CH_2); **HRMS** (ESI) m/z: $[M + Na]^+$ Calcd for $C_{19}H_{15}NNaO_2S$ 344.0716; Found 344.0716, $[M + H]^+$ Calcd for $C_{19}H_{16}NO_2S$ 322.0896; Found 322.0898; $\mathbf{\nu_{max}}$ (thin film)/cm^{-1} 3163, 1678, 1603, 1583, 1510, 1467, 1436, 1259, 1180, 834, 728.

2-(4-Fluorophenyl)-2'-thioxospiro[cyclopentane-1,3'-indolin]-2-en-4-one (9c)

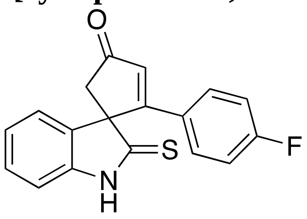

9c

Synthesized using **General Procedure B** with 1-(2-bromo-1*H*-indol-3-yl)-4-(4-fluorophenyl) but-3-yn-2-one (71.2 mg, 0.20 mmol), triphenylsilanethiol (93.6 mg, 0.32 mmol) and DCE (2 mL, 0.1 M) at 60 °C. Purification by flash column chromatography (hexane:EtOAc, 4:1 then 2:1 v/v) afforded the title product (52.3 mg, 85%) as an orange oil.

$\mathbf{R_f}$ 0.39 (hexane:EtOAc, 2:1 v/v); ^{1}**H NMR** (400 MHz, $CDCl_3$) δ 10.78 (1H, br s), 7.38 – 7.31 (1H, m), 7.17 – 7.10 (3H, m), 7.09 – 7.01 (2H, m), 6.91 – 6.83 (2H, m), 6.80 (1H, s), 3.17 (1H, d, *J* = 18.1 Hz), 2.82 (1H, d, *J* = 18.1 Hz); ^{13}**C NMR** (100 MHz, $CDCl_3$) δ 207.5 (C), 205.6 (C), 171.5 (C), 164.1 (C, d, C–F, $^{4}J_{C–F}$ = 253.2 Hz), 142.4 (C), 135.8 (C), 131.4 (CH), 129.8 (2CH, d, C–F, $^{3}J_{C–F}$ = 8.7 Hz), 129.7 (CH), 128.7 (C, d, C–F, $^{4}J_{C–F}$ = 3.4 Hz), 125.3 (CH), 123.7 (CH), 116.2 (2CH, d, C–F, $^{2}J_{C–F}$ = 22.4 Hz), 111.0 (CH), 68.1 (C), 53.0 (CH_2); ^{19}**F NMR**

(376 MHz, $CDCl_3$) δ −107.85 – −108.03 (1F, m); **HRMS** (ESI) m/z: $[M + Na]^+$ Calcd for $C_{18}H_{12}FNNaOS$ 332.0516; Found 332.0516, $[M + H]^+$ Calcd for $C_{18}H_{13}FNOS$ 310.0696; Found 310.0693; **ν_{max}** (thin film)/cm^{-1} 3177, 1685, 1601, 1507, 1467, 1437, 1236, 1162, 837, 753, 729.

5'-Bromo-2-phenyl-2'-thioxospiro[cyclopentane-1,3'-indolin]-2-en-4-one (9d)

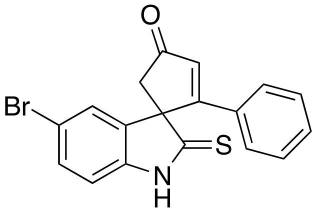

9d

Synthesized using **General Procedure B** with 1-(2,5-dibromo-1*H*-indol-3-yl)-4-phenylbut-3-yn-2-one (83.4 mg, 0.20 mmol), triphenylsilanethiol (93.6 mg, 0.32 mmol) and DCE (2 mL, 0.1 M) at 60 °C. Purification by flash column chromatography (hexane:EtOAc, 4:1 then 2:1 v/v) afforded the title product (52.4 mg, 71%) as a pink solid.

mp: 247 – 249 °C; **R_f** 0.44 (hexane:EtOAc, 2:1 v/v); **^{1}H NMR** (400 MHz, DMSO-d_6) δ 13.20 (1H, br s), 7.53 – 7.47 (2H, m), 7.36 – 7.24 (3H, m), 7.13 – 7.05 (3H, m), 7.04 (1H, s), 2.84 (1H, d, J = 17.9 Hz), 2.72 (1H, d, J = 17.9 Hz); **^{13}C NMR** (100 MHz, DMSO-d_6) δ 207.2 (C), 204.5 (C), 170.1 (C), 142.7 (C), 138.4 (C), 132.3 (C), 132.1 (CH), 131.8 (CH), 130.9 (CH), 128.9 (2CH), 127.2 (2CH), 126.9 (CH), 116.8 (C), 112.8 (CH), 67.6 (C), 52.1 (CH_2); **HRMS** (ESI) m/z: $[M + Na]^+$ Calcd for $C_{18}H_{12}{}^{79}BrNNaOS$ 391.9715; Found 391.9718; **ν_{max}** (thin film)/cm^{-1} 3187, 1689, 1457, 1403, 1331, 766.

2-Phenyl-2'-(*p*-tolylthio)spiro[cyclopentane-1,3'-indol]-2-en-4-one (6a)

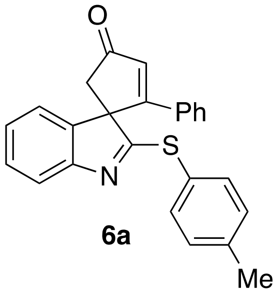

6a

Synthesized using **General Procedure B** with 1-(2-iodo-1*H*-indol-3-yl)-4-phenylbut-3-yn-2-one (77.0 mg, 0.20 mmol), 4-methylbenzenethiol (39.7 mg, 0.32 mmol) and DCE (2 mL, 0.1 M) at RT. Purification by flash column chromatography (hexane:EtOAc, 4:1 then 2:1 v/v) afforded the title product (55.0 mg, 72%) as a yellow solid.

mp: 164 – 166 °C; **R_f** 0.48 (hexane:EtOAc, 4:1 v/v); **^{1}H NMR** (400 MHz, $CDCl_3$) δ 7.51 (1H, br d, *J* = 7.8 Hz), 7.40 – 7.29 (4H, m), 7.25 – 7.18 (4H, m), 7.16 – 7.05 (4H, m), 6.83 (1H, s), 3.17 (1H, d, *J* = 18.6 Hz), 2.82 (1H, d, *J* = 18.6 Hz), 2.38 (3H, s); **^{13}C NMR** (100 MHz, $CDCl_3$) δ 204.8 (C), 183.7 (C), 172.2 (C), 154.8 (C), 141.8 (C), 140.1 (C), 134.6 (2CH), 132.4 (C), 131.4 (CH), 131.1 (CH), 130.4 (2CH), 129.1 (CH), 129.0 (2CH), 127.4 (2CH), 125.8 (CH), 123.5 (C), 121.4 (CH), 120.3 (CH), 67.4 (C), 48.3 (CH_2), 21.5 (CH_3); **HRMS** (ESI) m/z: [M + Na]$^+$ Calcd for $C_{25}H_{19}NNaOS$ 404.1080; Found 404.1074, [M + H]$^+$ Calcd for $C_{25}H_{20}NOS$ 382.1260; Found 382.1258; $\mathbf{v_{max}}$ (thin film)/cm^{-1} 3060, 1721, 1696, 1510, 1493, 1448, 953, 762.

2-Phenylspiro[cyclopentane-1,3'-indolin]-2-ene-2',4-dione (7a)

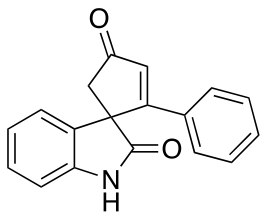

7a

Synthesized using **General Procedure B** with 1-(2-bromo-1*H*-indol-3-yl)-4-phenylbut-3-yn-2-one (67.6 mg, 0.20 mmol), phenol (30.1 mg, 0.32 mmol), trifluoroacetic acid (15.3 μL, 0.20 mmol) and DCE (2 mL, 0.1 M). Purification by flash column chromatography (hexane:EtOAc, 4:1 then 2:1 v/v) afforded the title product (33.9 mg, 62%) as a brown oil.

R_f 0.14 (hexane:EtOAc, 2:1 v/v); **^{1}H NMR** (400 MHz, $CDCl_3$) δ 9.24 (1H, br s), 7.34 – 7.17 (6H, m), 7.09 – 6.98 (3H, m), 6.82 (1H, s), 3.14 (1H, d, *J* = 18.2 Hz), 2.74 (1H, d, *J* = 18.2 Hz); **^{13}C NMR** (100 MHz, $CDCl_3$) δ 205.3 (C), 179.5 (C), 172.0 (C), 140.5 (C), 132.5 (C), 131.7 (CH), 131.6 (C), 131.2 (CH), 129.4 (CH), 129.1 (2CH), 127.4 (2CH), 123.9 (CH), 123.3 (CH), 111.0 (CH), 58.3 (C), 49.0 (CH_2); **HRMS** (ESI) m/z: [M + Na]$^+$ Calcd for $C_{18}H_{13}NNaO_2$ 298.0838; Found 298.0838, [M + H]$^+$ Calcd for $C_{18}H_{14}NO_2$ 276.1019; Found 276.1019; $\mathbf{v_{max}}$ (thin film)/cm^{-1} 3229, 1712, 1696, 1619, 1592, 1570, 1324, 1204, 750, 731.

4-Bromo-1-phenyl-1,2-dihydro-3*H*-cyclopenta[*c*]quinolin-3-one (8)

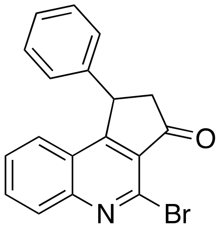

8

Synthesized using **General Procedure B** with 1-(2-bromo-1*H*-indol-3-yl)-4-phenylbut-3-yn-2-one (67.6 mg, 0.20 mmol), phenol (30.1 mg, 0.32 mmol), trifluoroacetic acid (15.3 μL, 0.20 mmol) and DCE (2 mL, 0.1 M) at RT. Purification by flash column chromatography (hexane:EtOAc, 4:1 then 2:1 v/v) afforded the title product (14.2 mg, 21%) as a yellow solid.
mp: 146 – 149 °C; $\mathbf{R_f}$ 0.48 (hexane:EtOAc, 2:1 v/v); **^{1}H NMR** (400 MHz, $CDCl_3$) δ 8.14 (1H, br d, *J* = 8.4 Hz), 7.83 – 7.78 (1H, m), 7.70 (1H, ddd, *J* = 8.4, 1.4, 0.6 Hz), 7.51 – 7.44 (1H, m), 7.34 – 7.23 (3H, m), 7.14 – 7.07 (2H, m), 4.96 (1H, dd, *J* = 8.2, 2.8 Hz), 3.42 (1H, dd, *J* = 19.2, 8.2 Hz), 2.80 (1H, dd, *J* = 19.2, 2.8 Hz); **^{13}C NMR** (100 MHz, $CDCl_3$) δ 200.7 (C), 168.8 (C), 150.8 (C), 142.3 (C), 136.6 (C), 133.5 (CH), 129.7 (CH), 129.6 (CH), 129.5 (CH), 129.0 (C), 128.0 (C), 127.7 (CH), 127.5 (CH), 127.4 (CH), 125.8 (CH), 124.8 (C), 48.0 (CH_2), 42.7 (CH); **HRMS** (ESI) m/z: $[M + Na]^+$ Calcd for $C_{18}H_{12}{}^{79}BrNNaO$ 359.9994; Found 359.9997, $[M + H]^+$ Calcd for $C_{18}H_{13}{}^{79}BrNO$ 338.0175; Found 338.0176; $\mathbf{\nu_{max}}$ (thin film)/cm^{-1} 3063, 2924, 1721, 1613, 1568, 1559, 1498, 1404, 1089, 762.

2-Phenyl-2'-(phenylthio)spiro[cyclopentane-1,3'-indol]-2-en-4-one (6a)

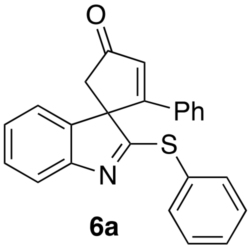

6a

Synthesized using **General Procedure B** with 1-(2-bromo-1*H*-indol-3-yl)-4-phenylbut-3-yn-2-one (67.6 mg, 0.20 mmol), thiolphenol (32.6 μL, 0.32 mmol), and MeCN (2 mL, 0.1 M) at RT. Purification by flash column chromatography (hexane:EtOAc, 6:1 v/v) afforded the title product (14.4 mg, 20%) as a yellow solid.
mp: 169 – 173 °C; $\mathbf{R_f}$ 0.50 (hexane:EtOAc, 2:1 v/v); **^{1}H NMR** (400 MHz, $CDCl_3$) δ 7.54 – 7.47 (3H, m), 7.43 – 7.37 (3H, m), 7.37 – 7.31 (2H, m), 7.27 – 7.20 (2H, m), 7.18 – 7.12 (2H, m), 7.11 – 7.05 (2H, m), 6.84 (1H, s), 3.18 (1H, d, *J* = 18.6 Hz), 2.83 (1H, d, *J* = 18.6 Hz); **^{13}C**

NMR (100 MHz, $CDCl_3$) δ 204.8 (C), 183.2 (C), 172.1 (C), 154.7 (C), 141.7. (C), 134.5 (2CH), 132.3 (C), 131.4 (CH), 131.1 (CH) 129.7 (CH), 129.6 (2CH), 129.2 (CH), 129.1 (2CH), 127.4 (2CH), 127.2 (C), 125.9 (CH), 121.4 (CH), 120.3 (CH), 67.5 (C), 48.2 (CH_2); **HRMS** (ESI) m/z: $[M + Na]^+$ Calcd for $C_{24}H_{17}NNaOS$ 390.0923; Found 390.0927, $[M + H]^+$ Calcd for $C_{24}H_{18}NOS$ 368.1104; Found 368.1106; $\mathbf{\nu_{max}}$ (thin film)/cm^{-1} 3059, 2923, 1722, 1697, 1511, 1448, 1261, 953, 762, 747, 687.

Spectroscopic data matched those previously reported in the literature.[1, 2]

^{1}H, ^{13}C and ^{19}F NMR Spectra

^{1}H NMR spectrum of **1-(2-Bromo-1*H*-indol-3-yl)-4-(4-methoxyphenyl)but-3-yn-2-one (1b$_{Br}$)** (400 MHz, $CDCl_3$)

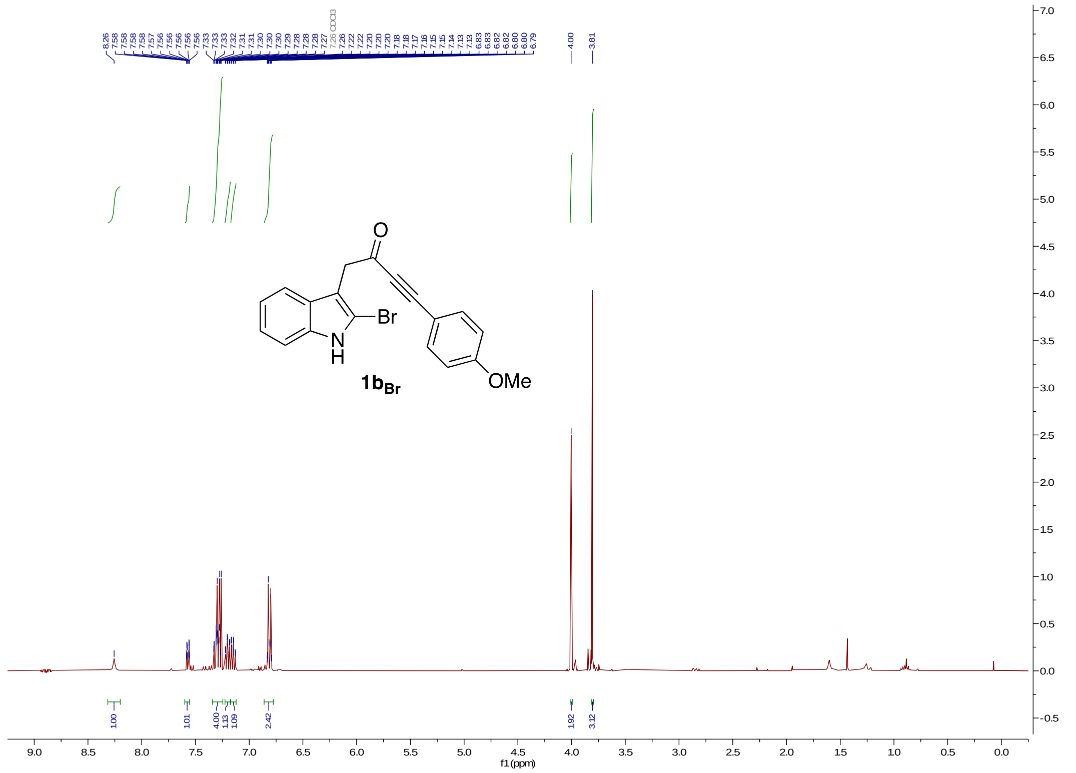

^{13}C NMR spectrum of **1-(2-Bromo-1*H*-indol-3-yl)-4-(4-methoxyphenyl)but-3-yn-2-one (1b$_{Br}$)** (100 MHz, $CDCl_3$)

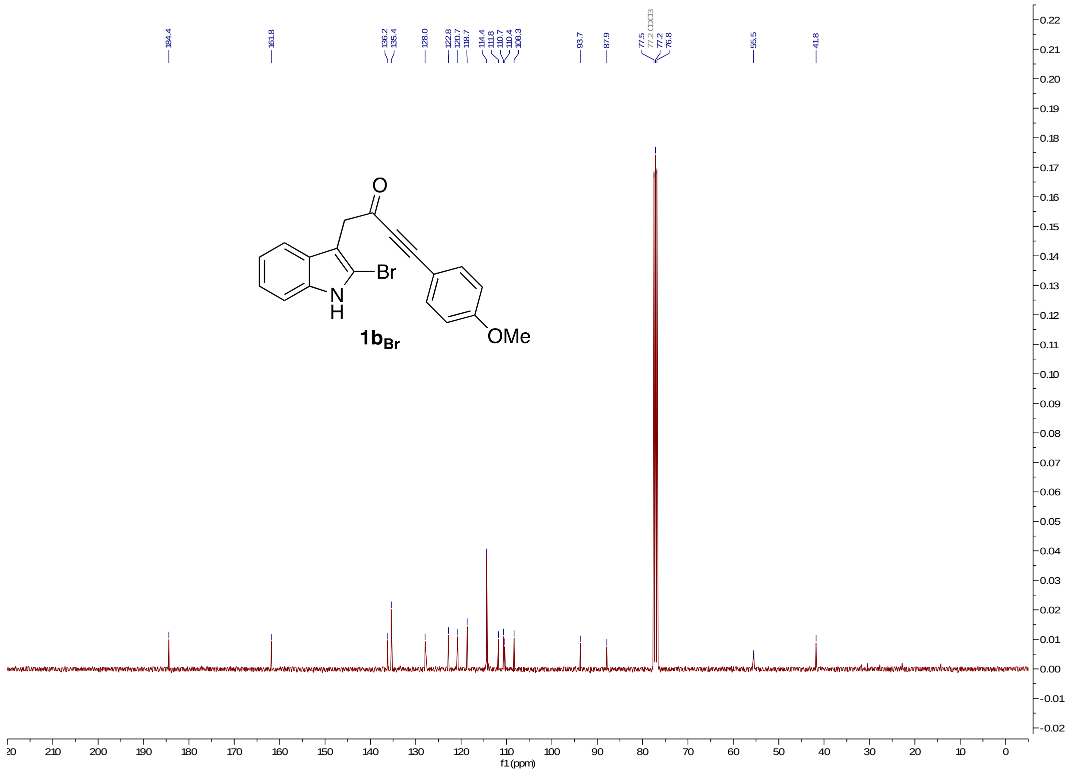

^{1}H NMR spectrum of **1-(2-Bromo-1*H*-indol-3-yl)-4-(4-fluorophenyl)but-3-yn-2-one ($1c_{Br}$)** (400 MHz, $CDCl_3$)

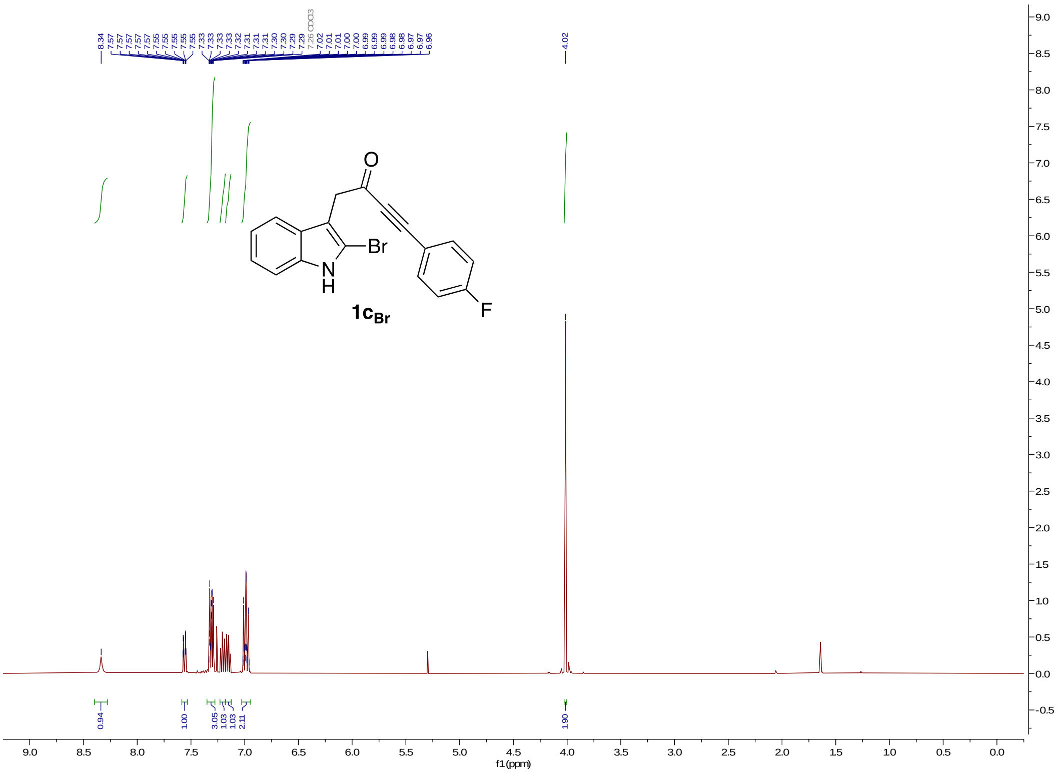

^{13}C NMR spectrum of **1-(2-Bromo-1*H*-indol-3-yl)-4-(4-fluorophenyl)but-3-yn-2-one ($1c_{Br}$)** (100 MHz, $CDCl_3$)

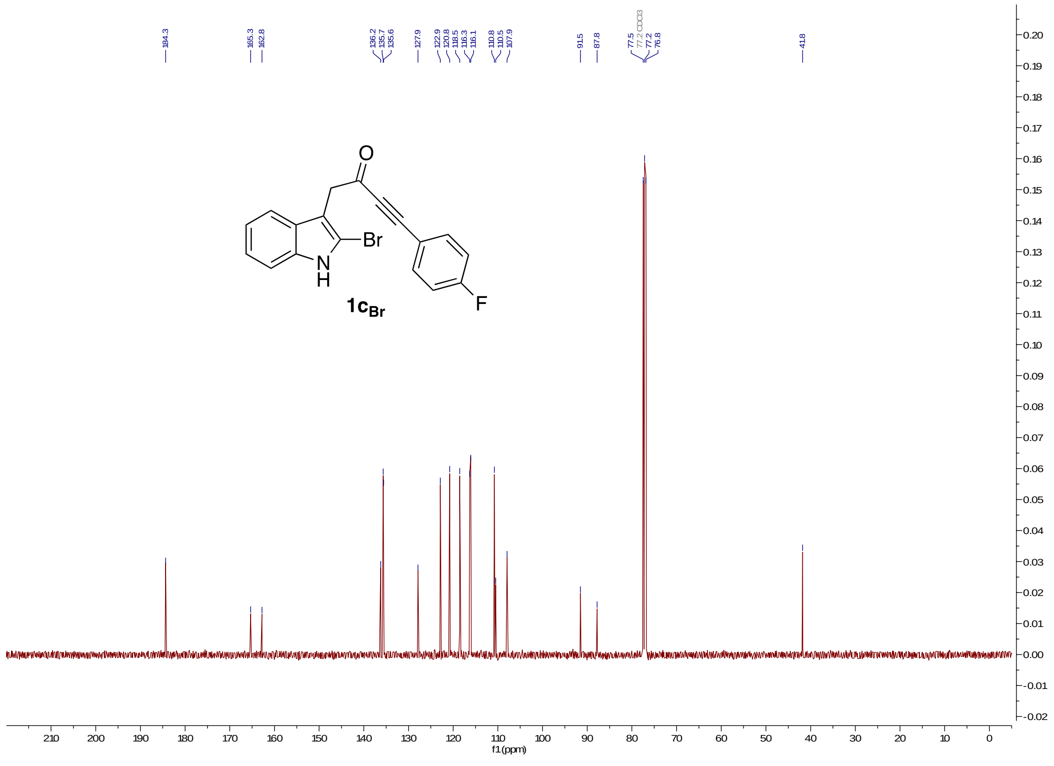

^{19}F NMR spectrum of **1-(2-Bromo-1*H*-indol-3-yl)-4-(4-fluorophenyl)but-3-yn-2-one (1c$_{Br}$)** (376 MHz, $CDCl_3$)

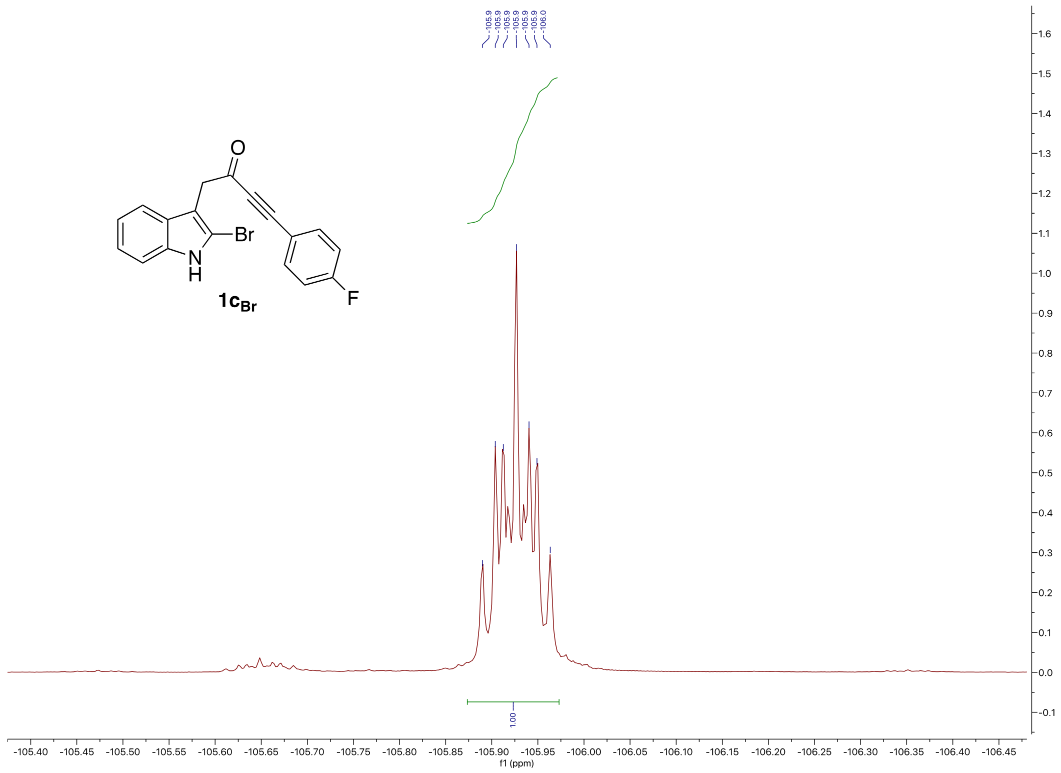

^{1}H NMR spectrum of **1-(2-Bromo-1*H*-indol-3-yl)-4-cyclopropylbut-3-yn-2-one ($1d_{Br}$)** (400 MHz, $CDCl_3$)

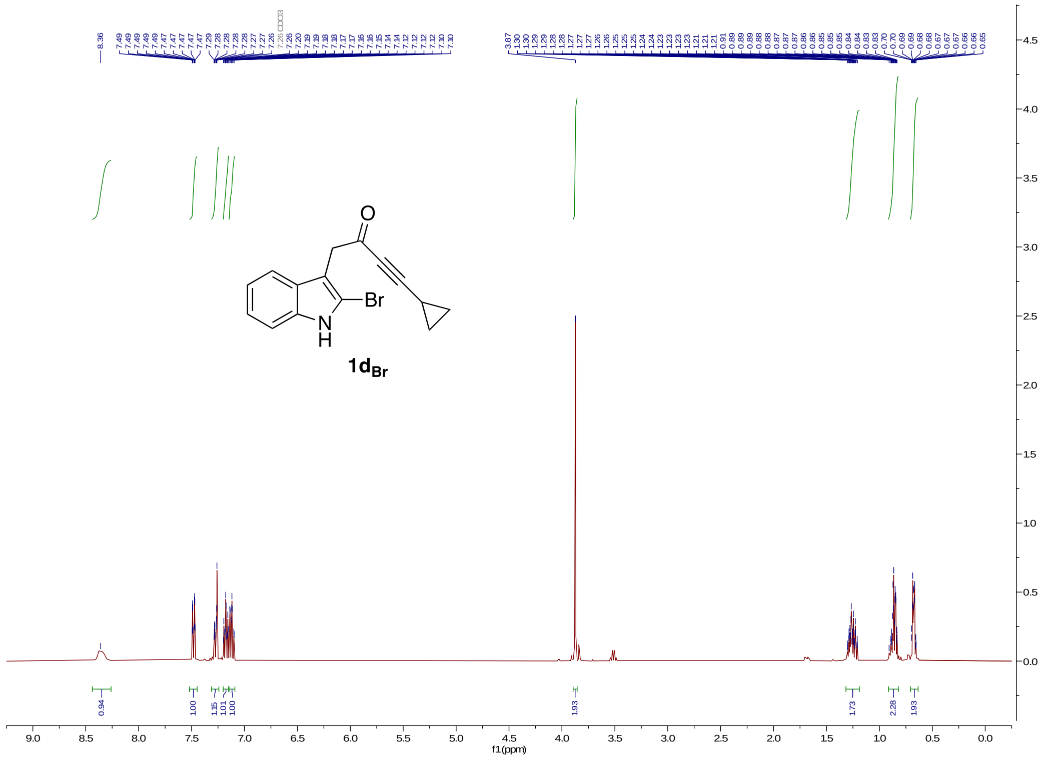

^{13}C NMR spectrum of **1-(2-Bromo-1*H*-indol-3-yl)-4-cyclopropylbut-3-yn-2-one ($1d_{Br}$)** (100 MHz, $CDCl_3$)

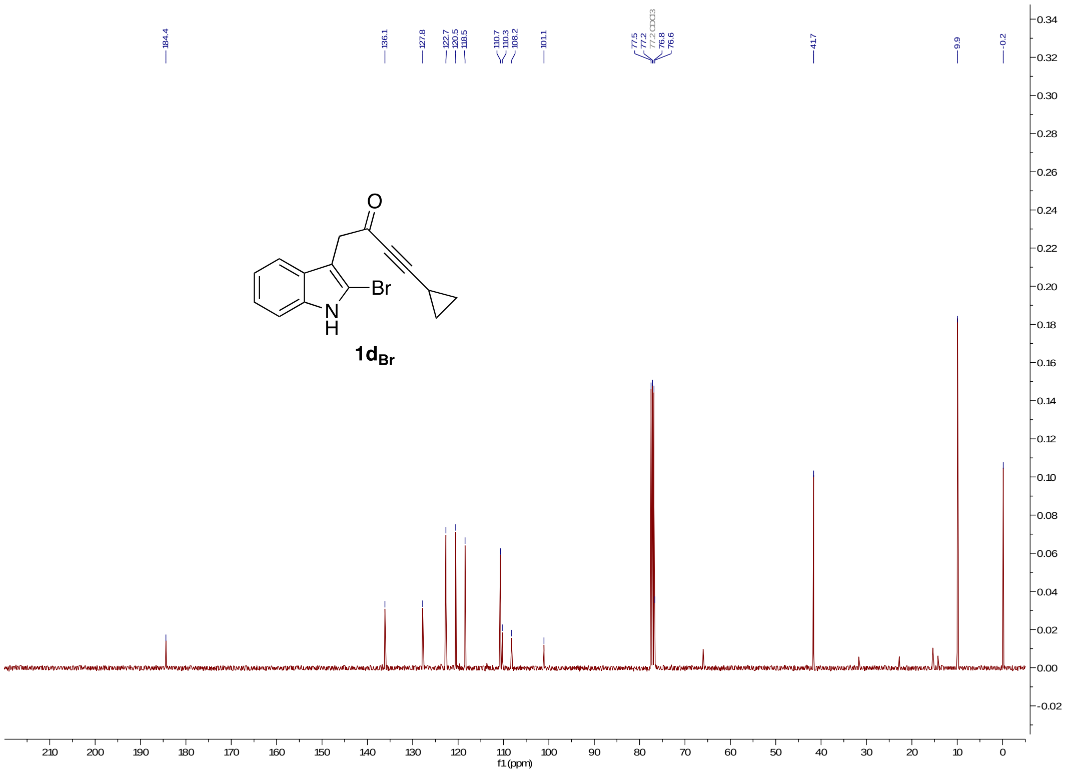

^{1}H NMR spectrum of **1-(2,5-dibromo-1*H*-indol-3-yl)-4-phenylbut-3-yn-2-one (1e$_{Br}$)** (400 MHz, $CDCl_3$)

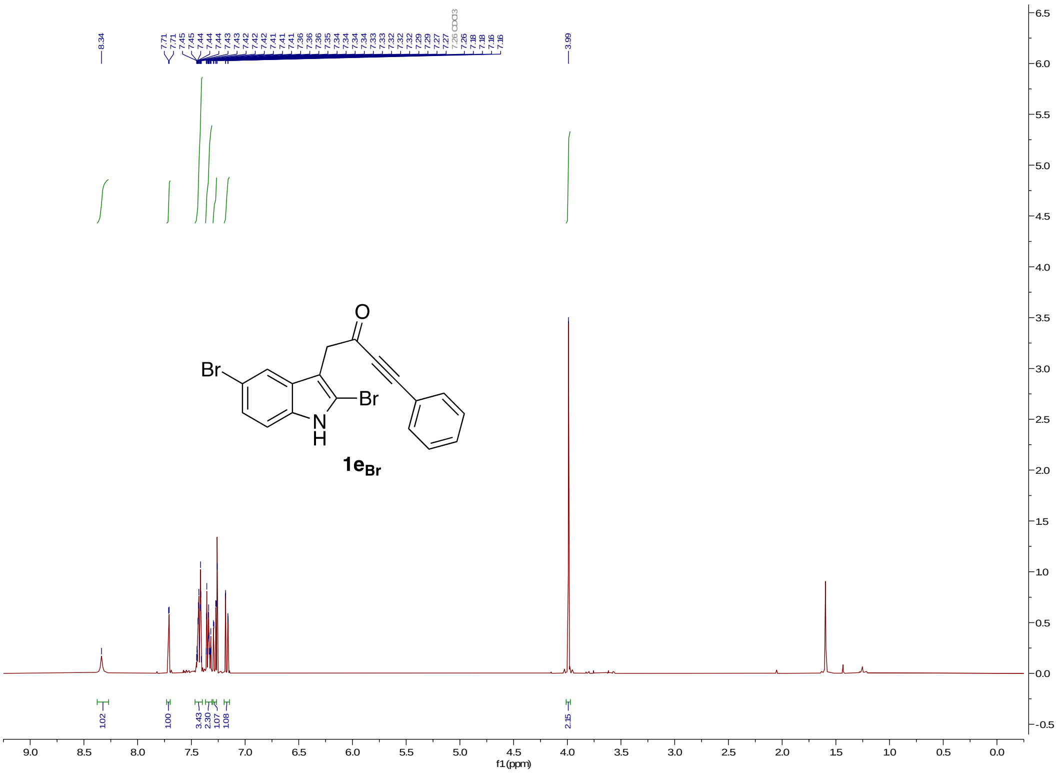

^{13}C NMR spectrum of **1-(2,5-dibromo-1*H*-indol-3-yl)-4-phenylbut-3-yn-2-one (1e$_{Br}$)** (100 MHz, $CDCl_3$)

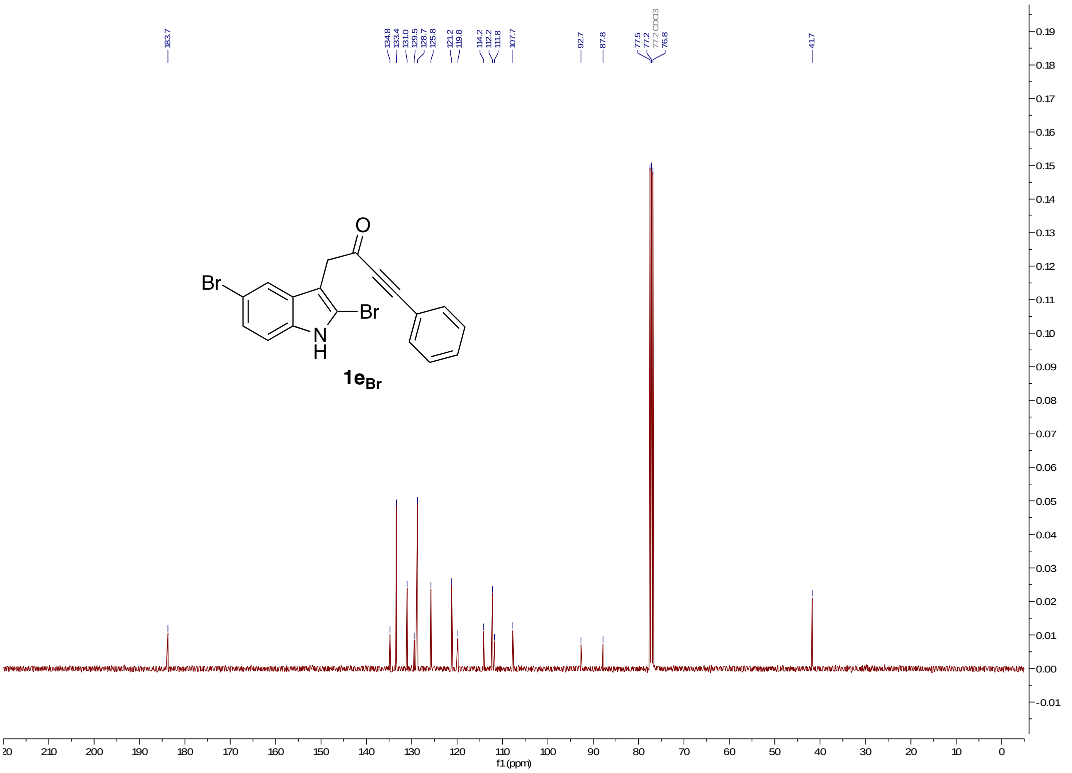

^{1}H NMR spectrum of **1-(2-bromo-1*H*-indol-3-yl)-4-(4-(dimethylamino)phenyl)but-3-yn-2-one (1f$_{Br}$)** (400 MHz, $CDCl_3$)

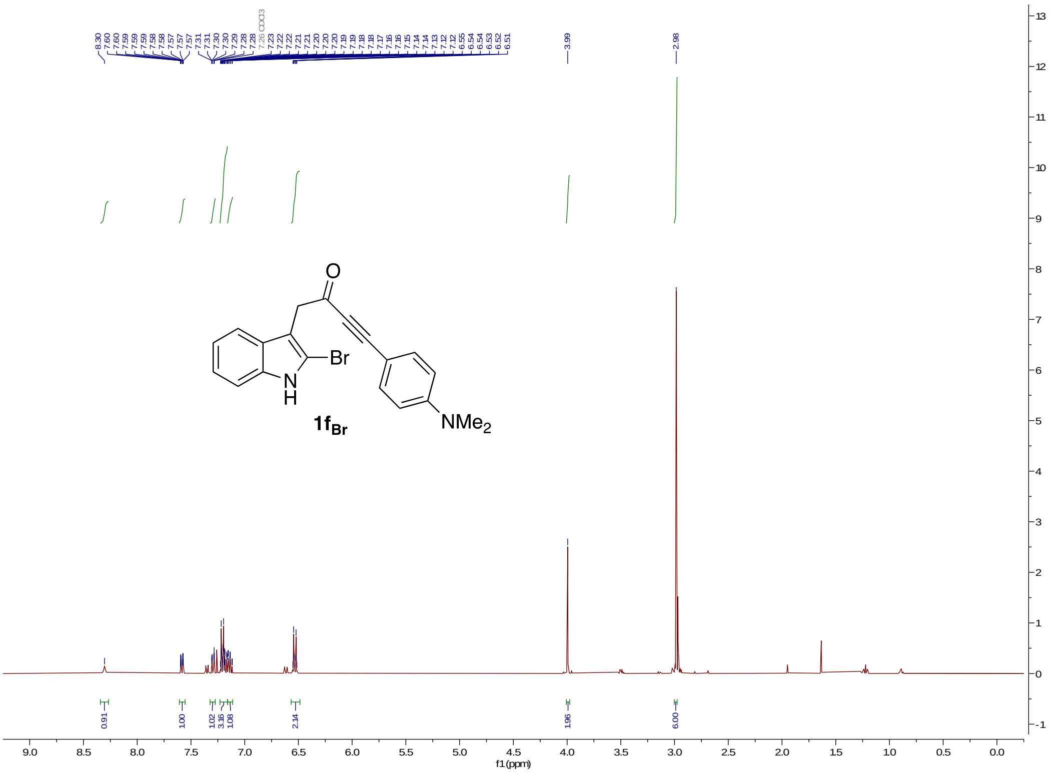

^{13}C NMR spectrum of **1-(2-bromo-1*H*-indol-3-yl)-4-(4-(dimethylamino)phenyl)but-3-yn-2-one (1f$_{Br}$)** (100 MHz, $CDCl_3$)

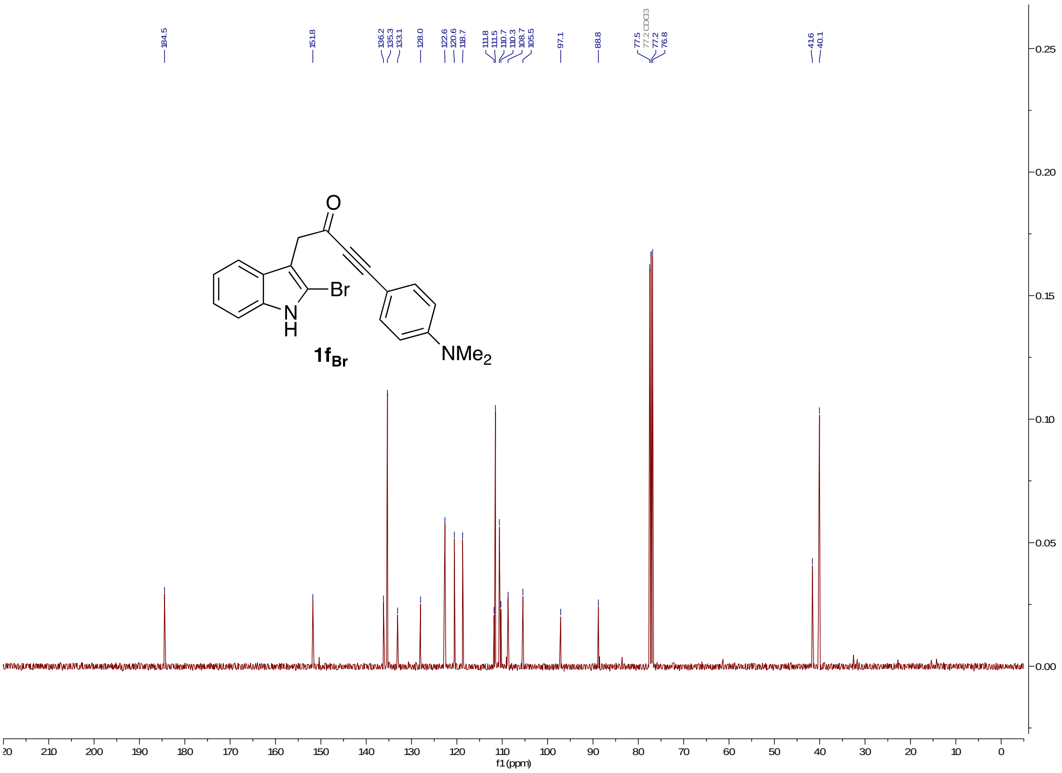

^{1}H NMR spectrum of **1-Phenyl-4-(phenylthio)-1,2-dihydro-3*H*-cyclopenta[*c*]quinolin-3-one (4b)** (400 MHz, $CDCl_3$)

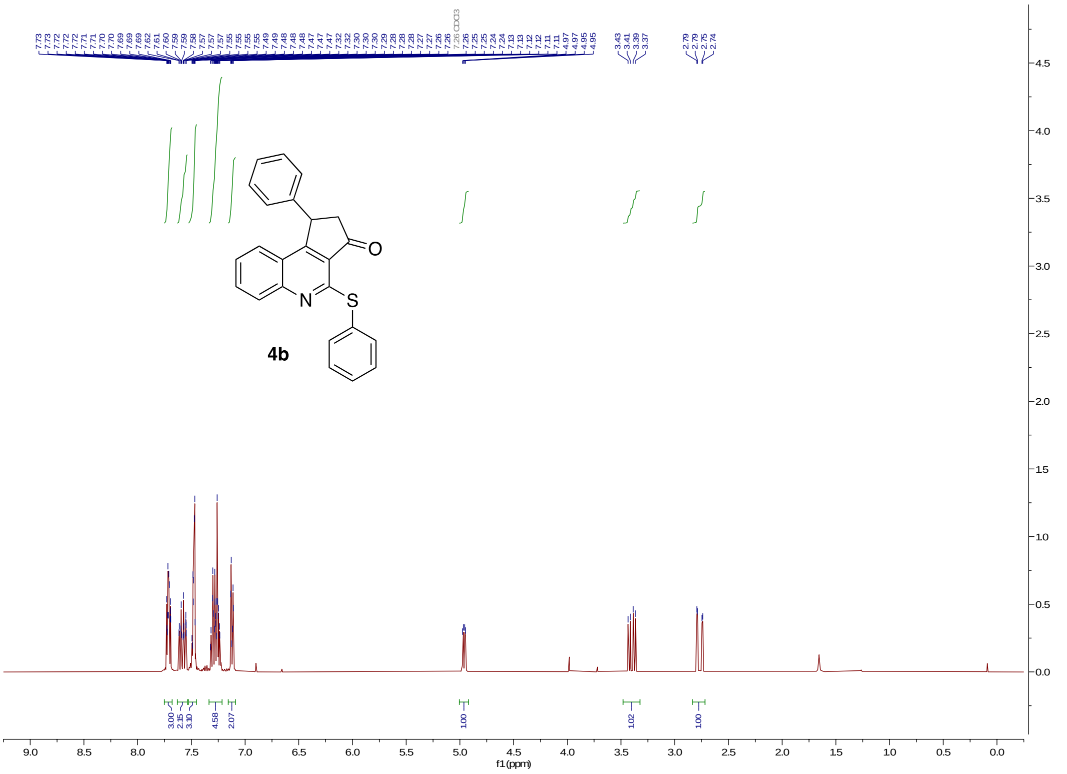

^{13}C NMR spectrum of **1-Phenyl-4-(phenylthio)-1,2-dihydro-3*H*-cyclopenta[*c*]quinolin-3-one (4b)** (100 MHz, $CDCl_3$)

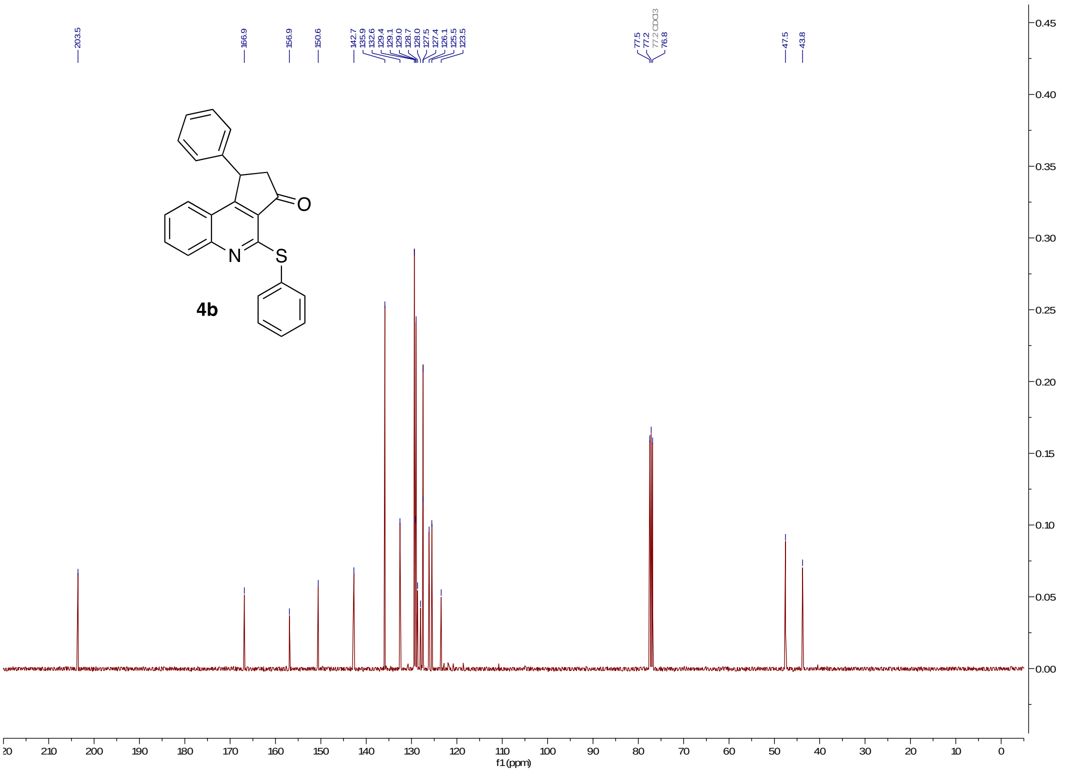

^{1}H NMR spectrum of **4-((4-(*tert*-Butyl)phenyl)thio)-1-phenyl-1,2-dihydro-3*H*-cyclopenta[*c*]quinolin-3-one (4c)** (400 MHz, $CDCl_3$)

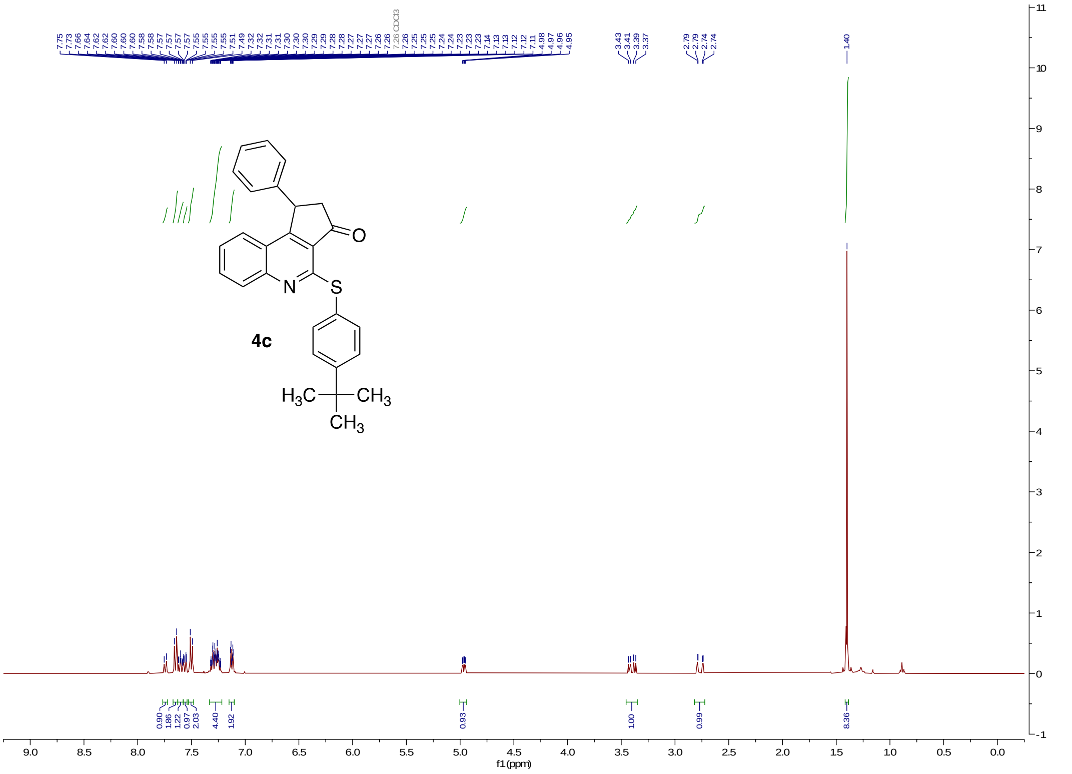

^{13}C NMR spectrum of **4-((4-(*tert*-Butyl)phenyl)thio)-1-phenyl-1,2-dihydro-3*H*-cyclopenta[*c*]quinolin-3-one (4c)** (100 MHz, $CDCl_3$)

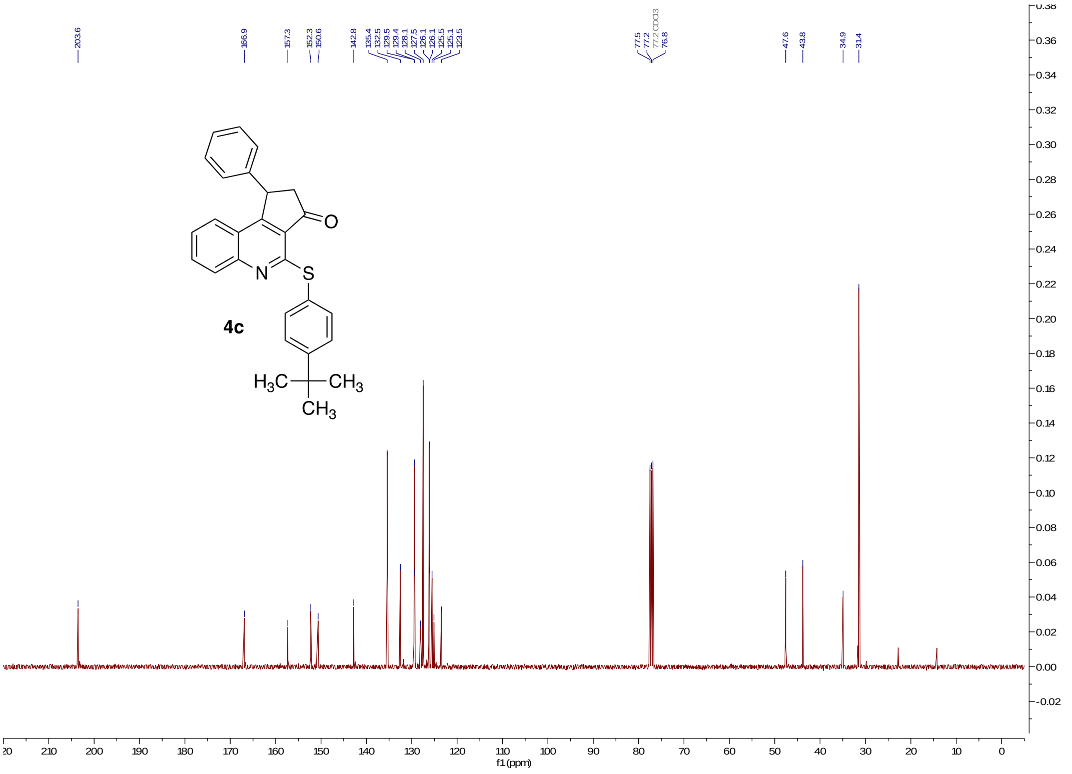

^{1}H NMR spectrum of **1-Phenyl-4-(*p*-tolylthio)-1,2-dihydro-3*H*-cyclopenta[*c*]quinolin-3-one (4d)** (400 MHz, $CDCl_3$)

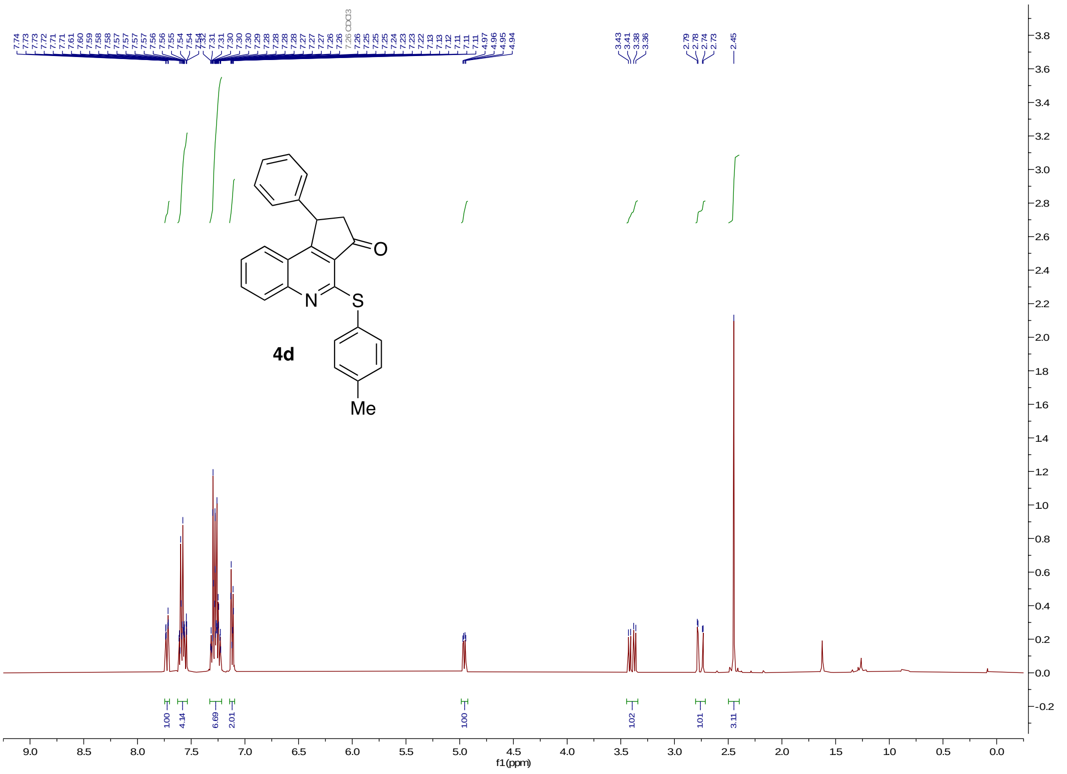

^{13}C NMR spectrum of **1-Phenyl-4-(*p*-tolylthio)-1,2-dihydro-3*H*-cyclopenta[*c*]quinolin-3-one (4d)** (100 MHz, $CDCl_3$)

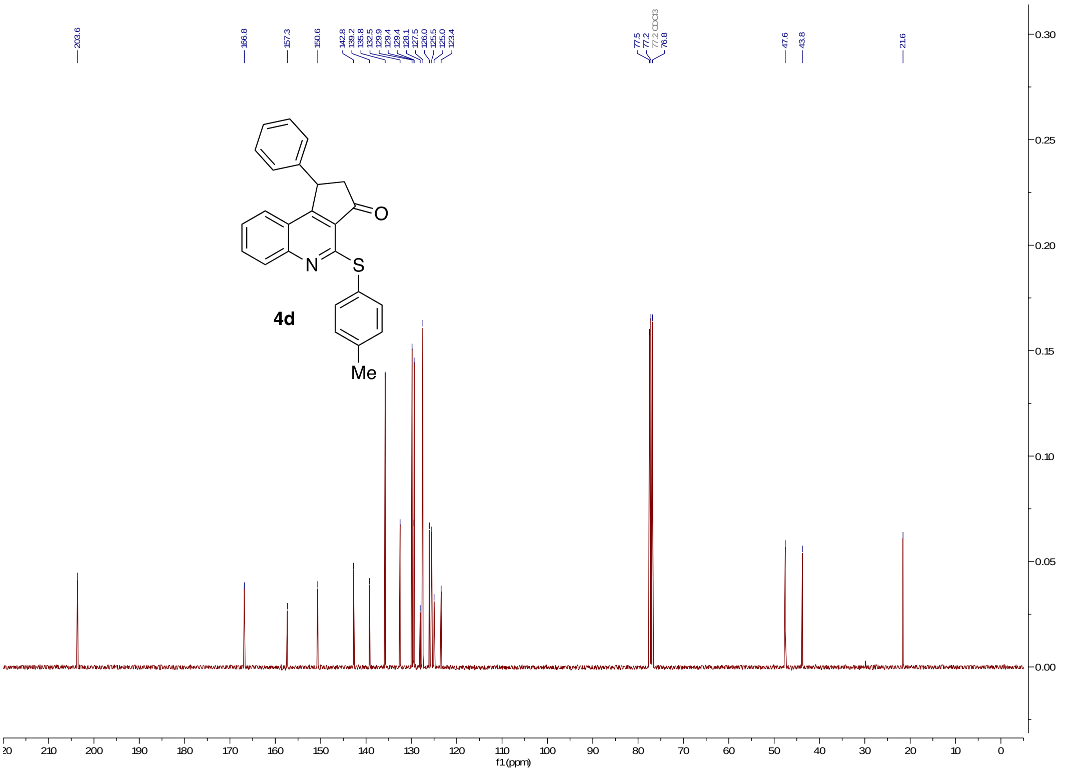

^{1}H NMR spectrum of **4-([1,1'-Biphenyl]-4-ylthio)-1-phenyl-1,2-dihydro-3*H*-cyclopenta[*c*]quinolin-3-one (4e)** (400 MHz, $CDCl_3$)

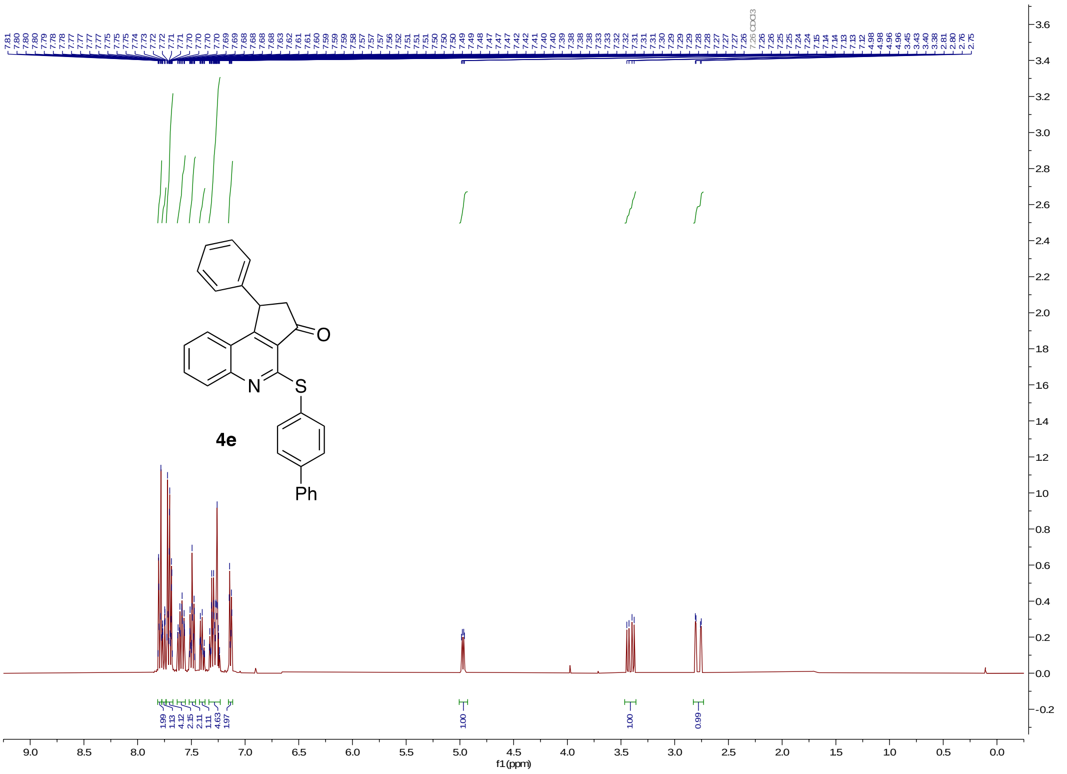

^{13}C NMR spectrum of **4-([1,1'-Biphenyl]-4-ylthio)-1-phenyl-1,2-dihydro-3*H*-cyclopenta[*c*]quinolin-3-one (4e)** (100 MHz, $CDCl_3$)

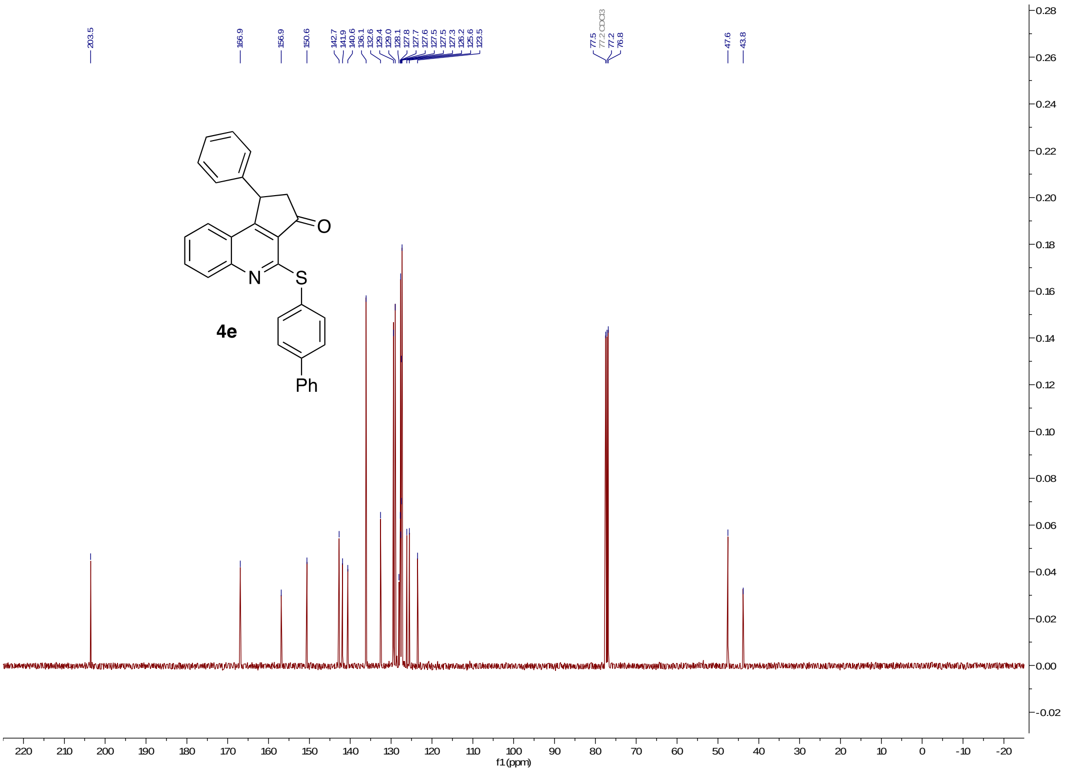

^{1}H NMR spectrum of **4-(Naphthalen-2-ylthio)-1-phenyl-1,2-dihydro-3*H*-cyclopenta[*c*]quinolin-3-one (4f)** (400 MHz, $CDCl_3$)

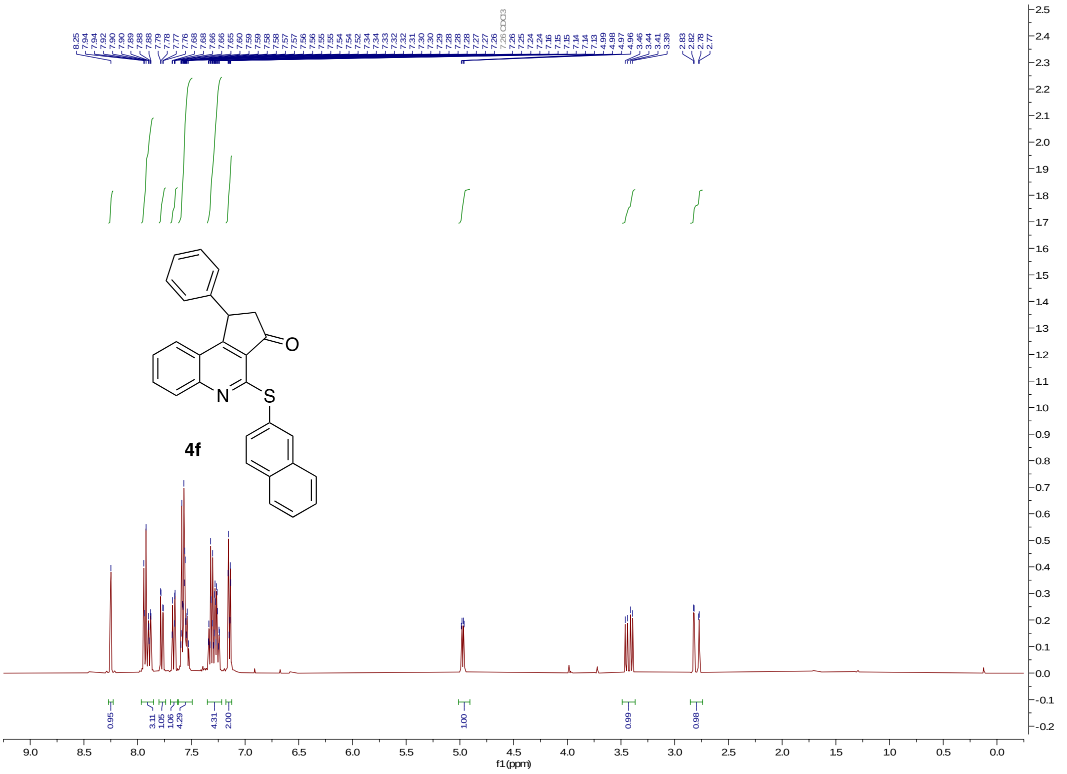

^{13}C NMR spectrum of **4-(Naphthalen-2-ylthio)-1-phenyl-1,2-dihydro-3*H*-cyclopenta[*c*]quinolin-3-one (4f)** (100 MHz, $CDCl_3$)

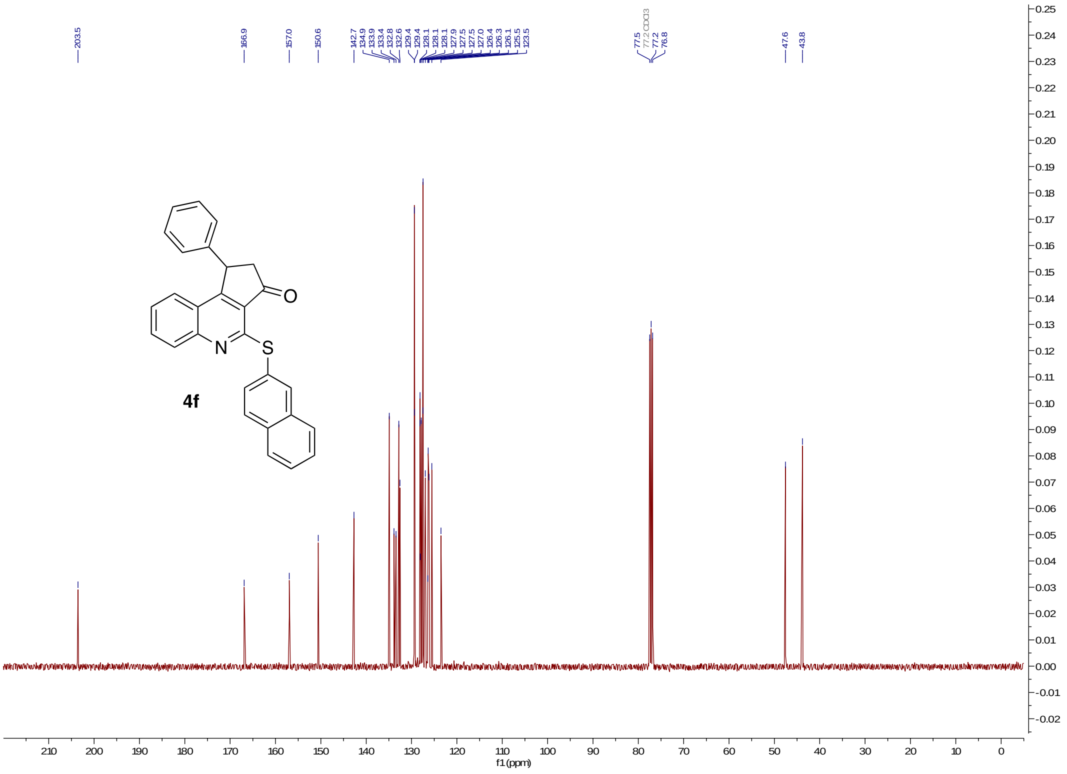

^{1}H NMR spectrum of **4-((4-Bromophenyl)thio)-1-phenyl-1,2-dihydro-3*H*-cyclopenta[*c*]quinolin-3-one (4g)** (400 MHz, $CDCl_3$)

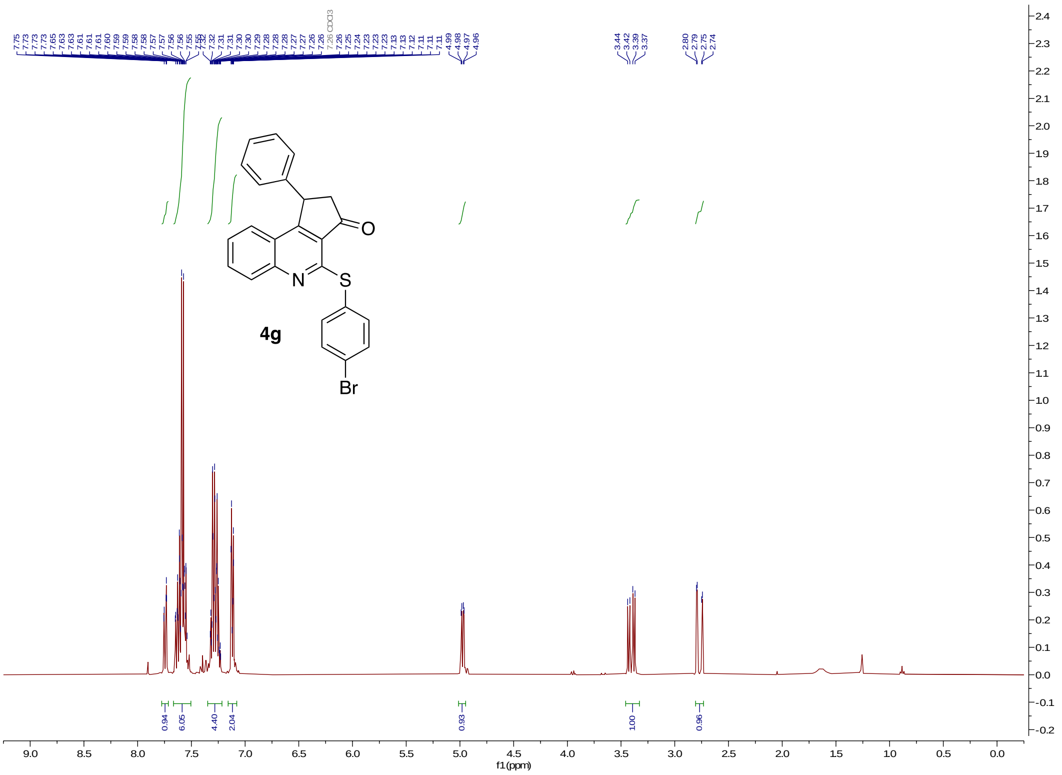

^{13}C NMR spectrum of **4-((4-Bromophenyl)thio)-1-phenyl-1,2-dihydro-3*H*-cyclopenta[*c*]quinolin-3-one (4g)** (100 MHz, $CDCl_3$)

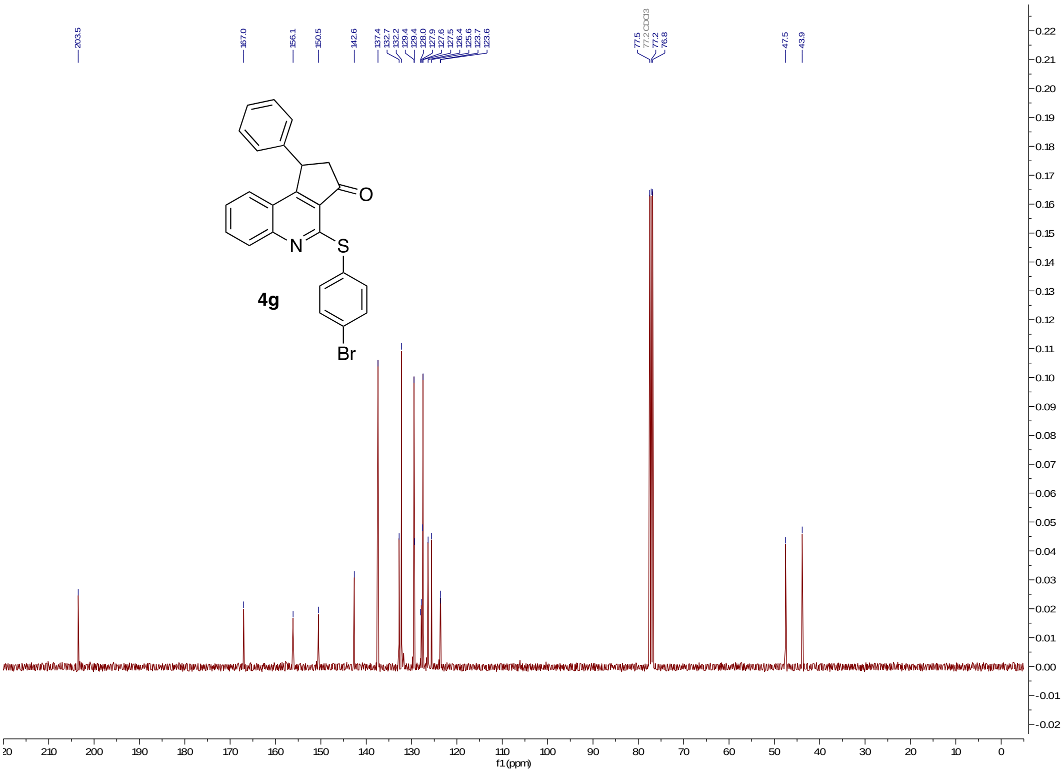

^{1}H NMR spectrum of **4-((4-Nitrophenyl)thio)-1-phenyl-1,2-dihydro-3*H*-cyclopenta[*c*]quinolin-3-one (4h)** (400 MHz, $CDCl_3$)

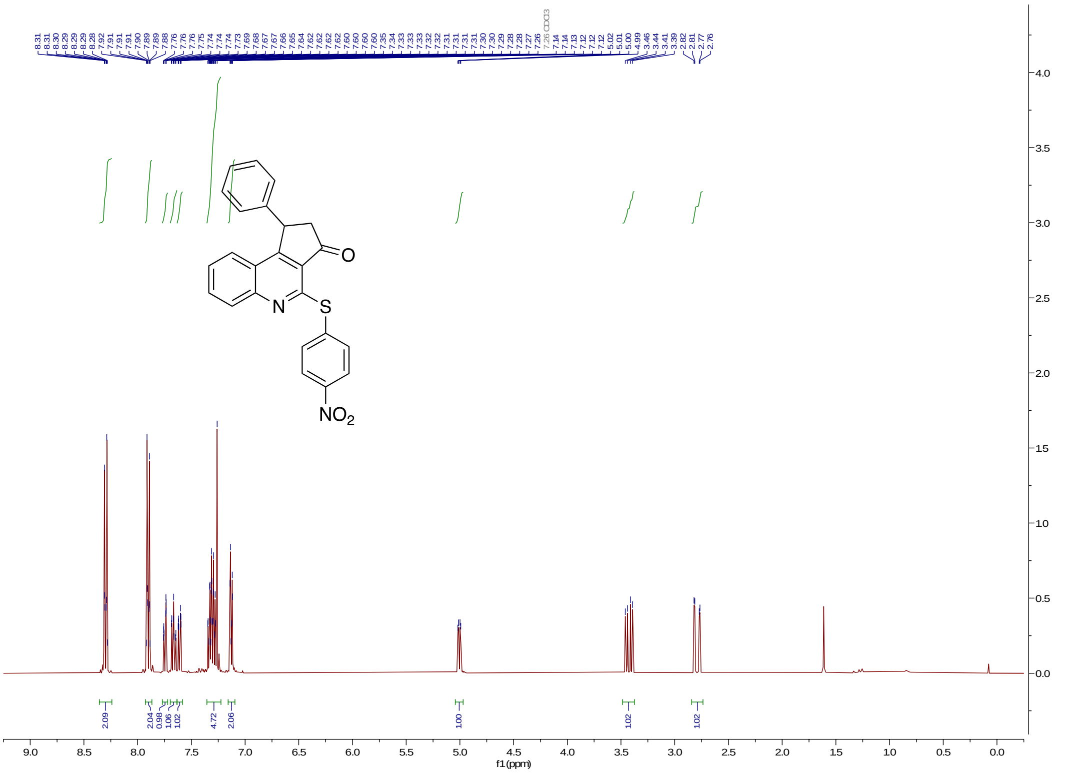

^{13}C NMR spectrum of **4-((4-Nitrophenyl)thio)-1-phenyl-1,2-dihydro-3*H*-cyclopenta[*c*]quinolin-3-one (4h)** (100 MHz, $CDCl_3$)

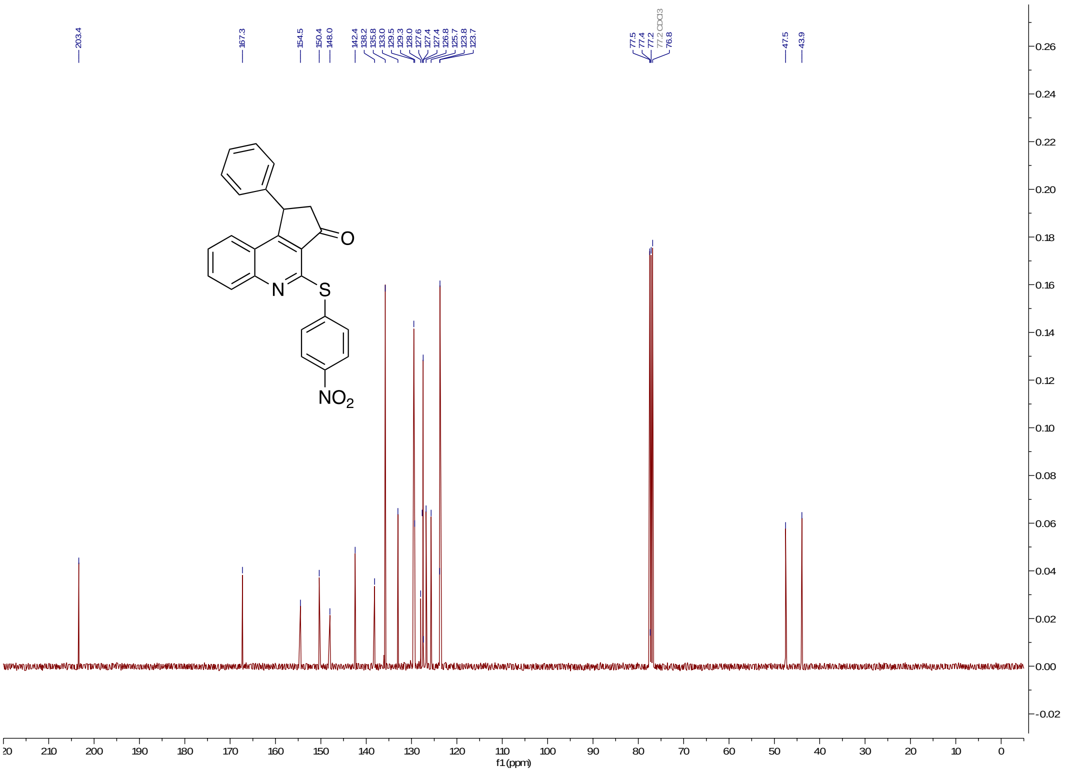

^{1}H NMR spectrum of **4-((3-Methoxyphenyl)thio)-1-phenyl-1,2-dihydro-3*H*-cyclopenta[*c*]quinolin-3-one (4i)** (400 MHz, $CDCl_3$)

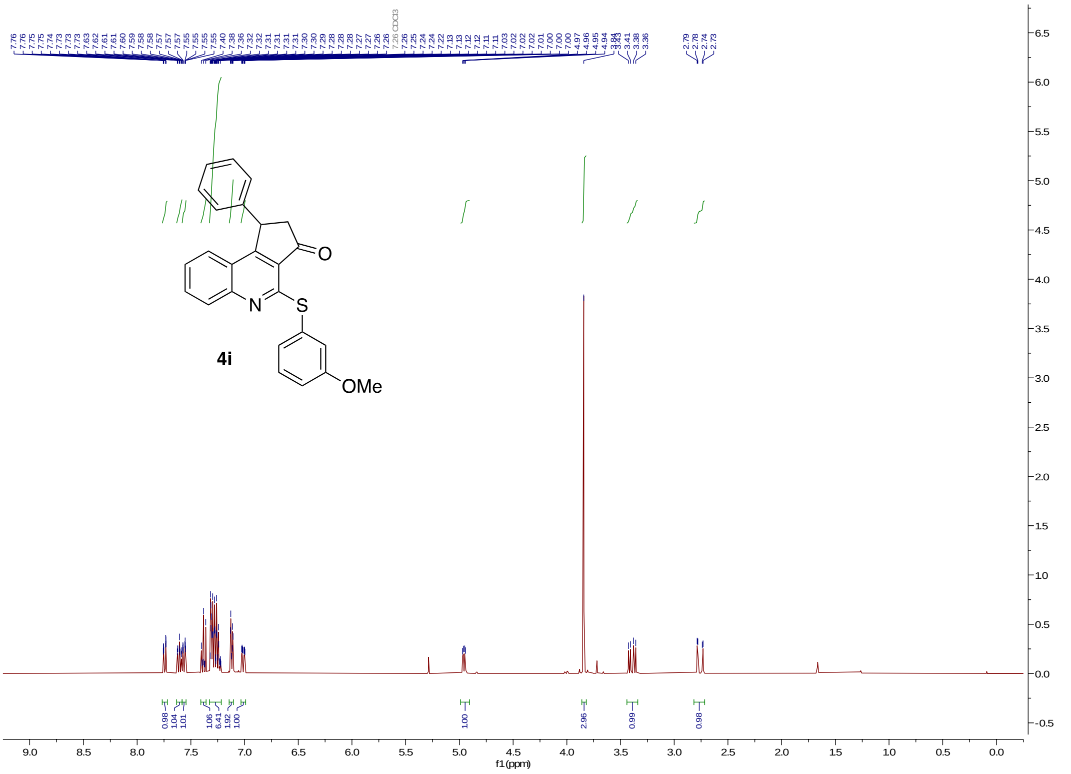

^{13}C NMR spectrum of **4-((3-Methoxyphenyl)thio)-1-phenyl-1,2-dihydro-3*H*-cyclopenta[*c*]quinolin-3-one (4i)** (100 MHz, $CDCl_3$)

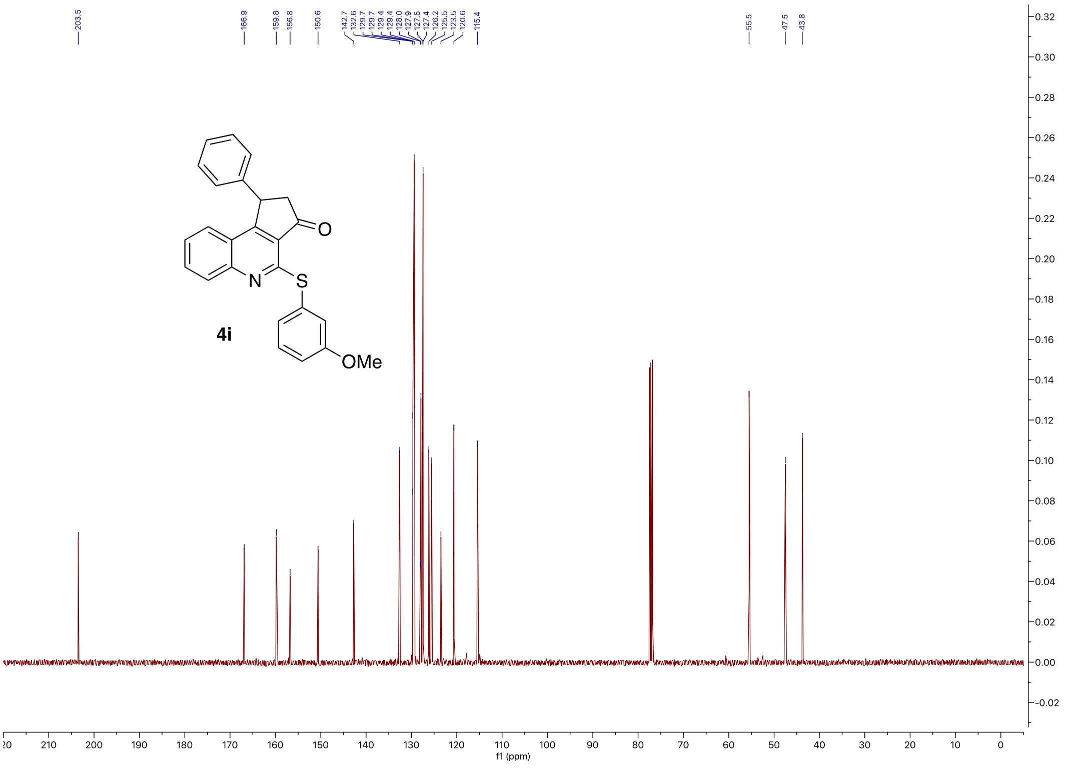

^{1}H NMR spectrum of **4-((2-Chlorophenyl)thio)-1-phenyl-1,2-dihydro-3*H*-cyclopenta[*c*]quinolin-3-one (4j)** (400 MHz, $CDCl_3$)

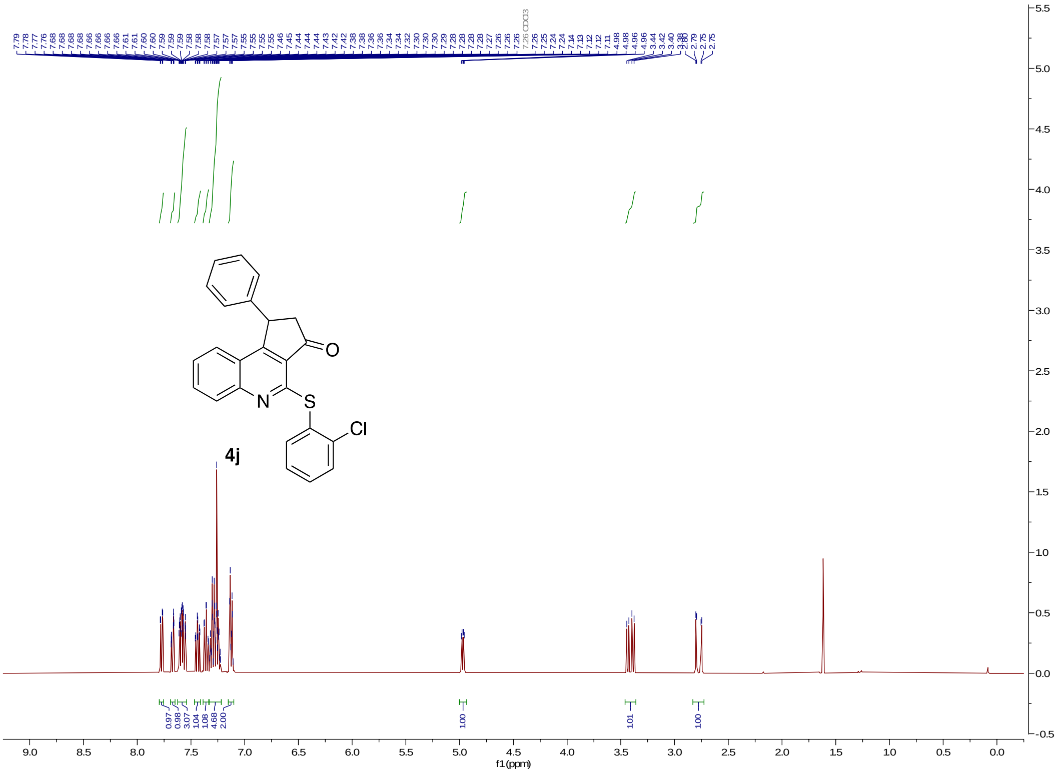

^{13}C NMR spectrum of **4-((2-Chlorophenyl)thio)-1-phenyl-1,2-dihydro-3*H*-cyclopenta[*c*]quinolin-3-one (4j)** (100 MHz, $CDCl_3$)

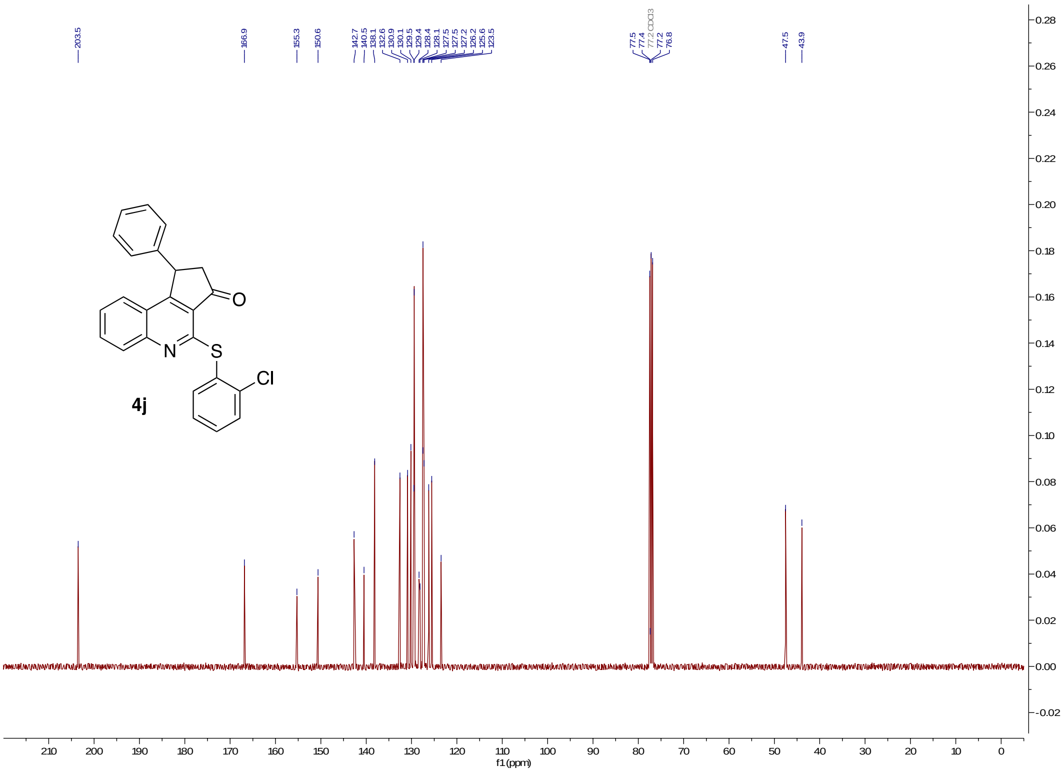

^{1}H NMR spectrum of **Methyl 2-((3-oxo-1-phenyl-2,3-dihydro-1*H*-cyclopenta[*c*]quinolin-4-yl)thio)benzoate (4k)** (400 MHz, $CDCl_3$)

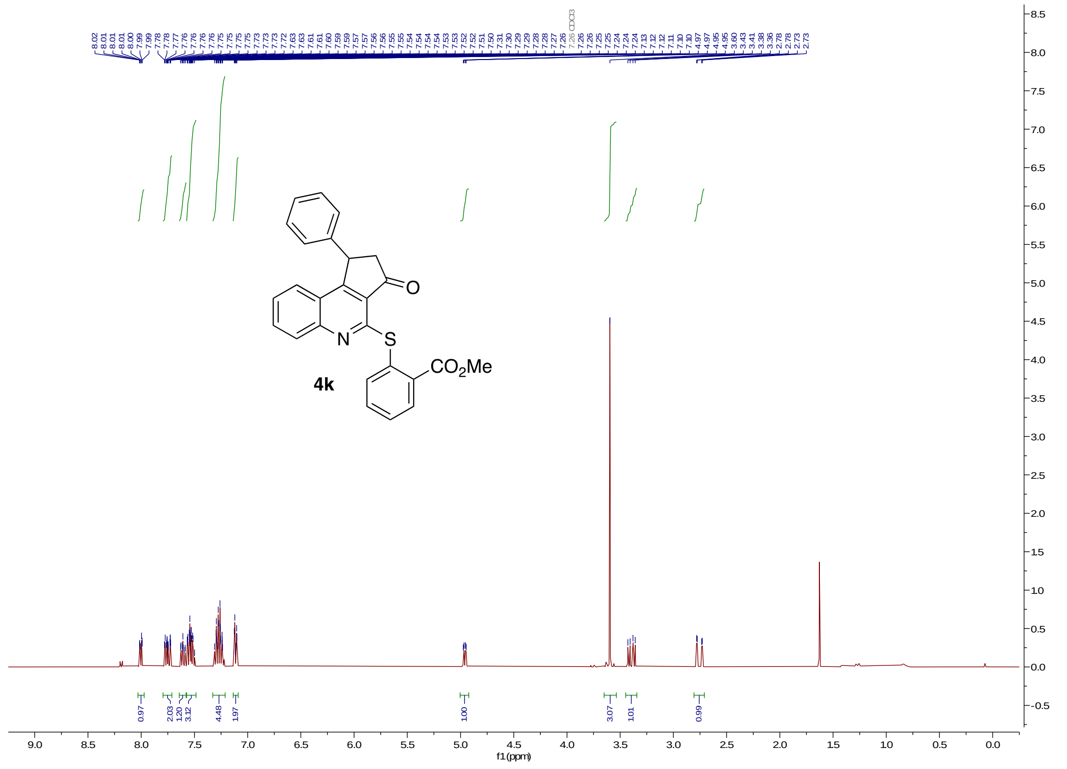

^{13}C NMR spectrum of **Methyl 2-((3-oxo-1-phenyl-2,3-dihydro-1*H*-cyclopenta[*c*]quinolin-4-yl)thio)benzoate (4k)** (100 MHz, $CDCl_3$)

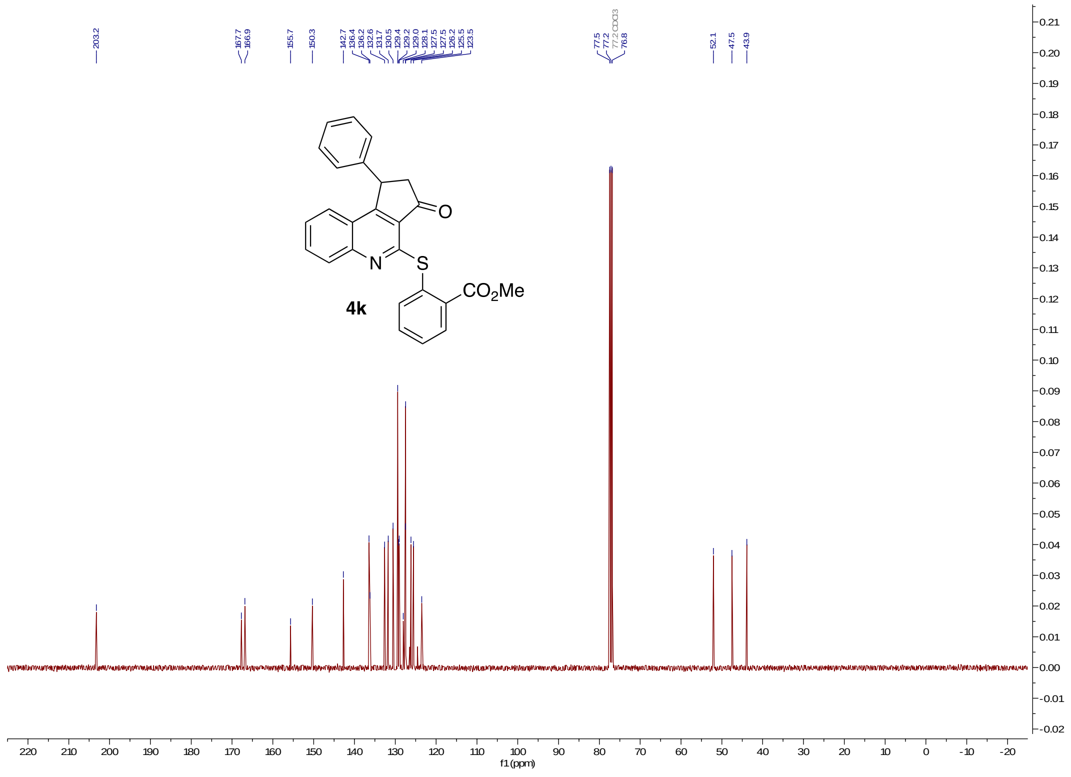

^{1}H NMR spectrum of **1-Phenyl-4-(propylthio)-1,2-dihydro-3*H*-cyclopenta[*c*]quinolin-3-one (4a)** (400 MHz, $CDCl_3$)

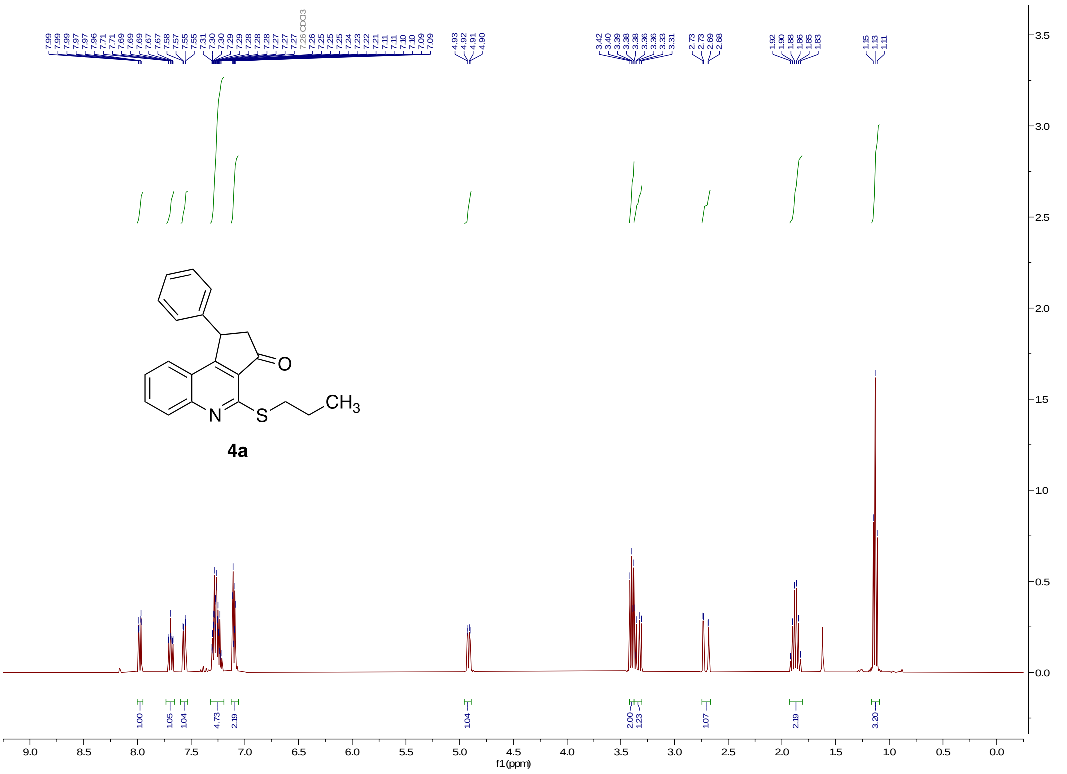

^{13}C NMR spectrum of **1-Phenyl-4-(propylthio)-1,2-dihydro-3*H*-cyclopenta[*c*]quinolin-3-one (4a)** (100 MHz, $CDCl_3$)

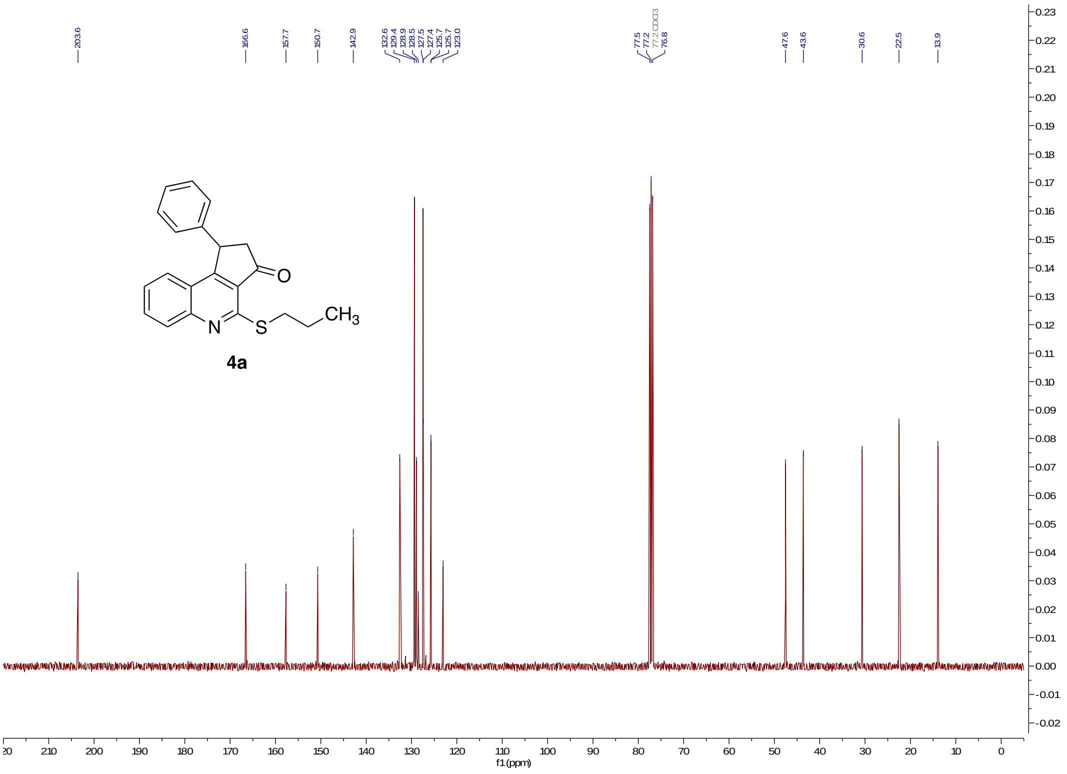

^{1}H NMR spectrum of **4-(Cyclohexylthio)-1-phenyl-1,2-dihydro-3*H*-cyclopenta[*c*]quinolin-3-one (4l)** (400 MHz, $CDCl_3$)

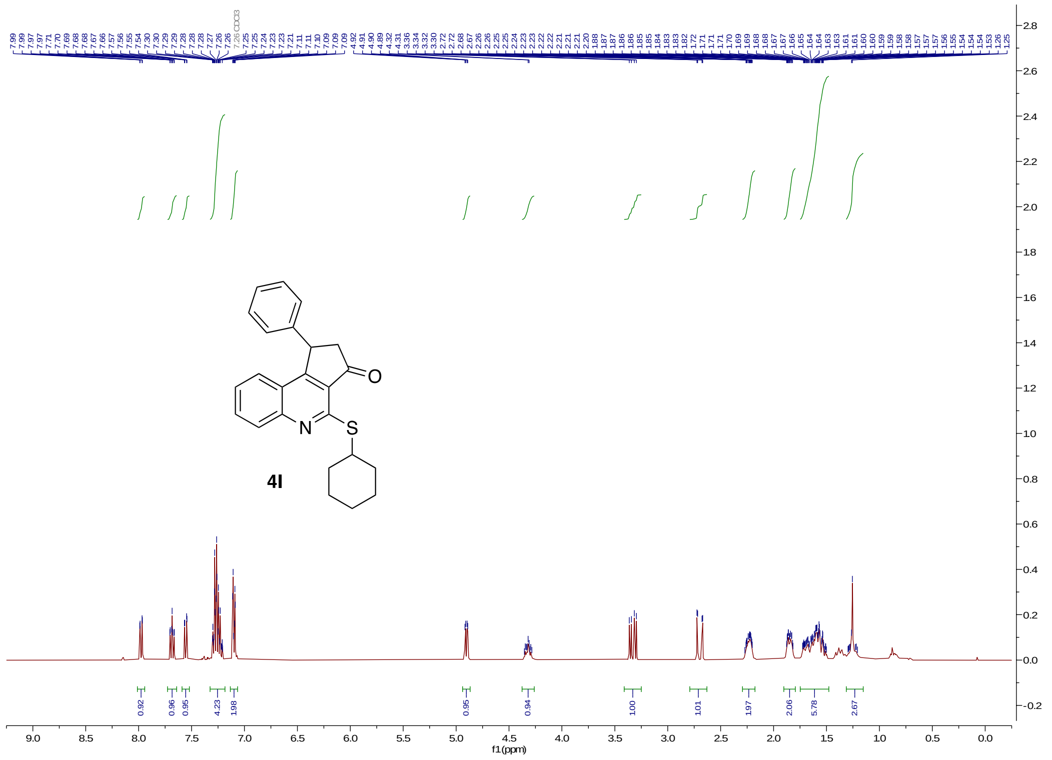

^{13}C NMR spectrum of **4-(Cyclohexylthio)-1-phenyl-1,2-dihydro-3*H*-cyclopenta[*c*]quinolin-3-one (4l)** (100 MHz, $CDCl_3$)

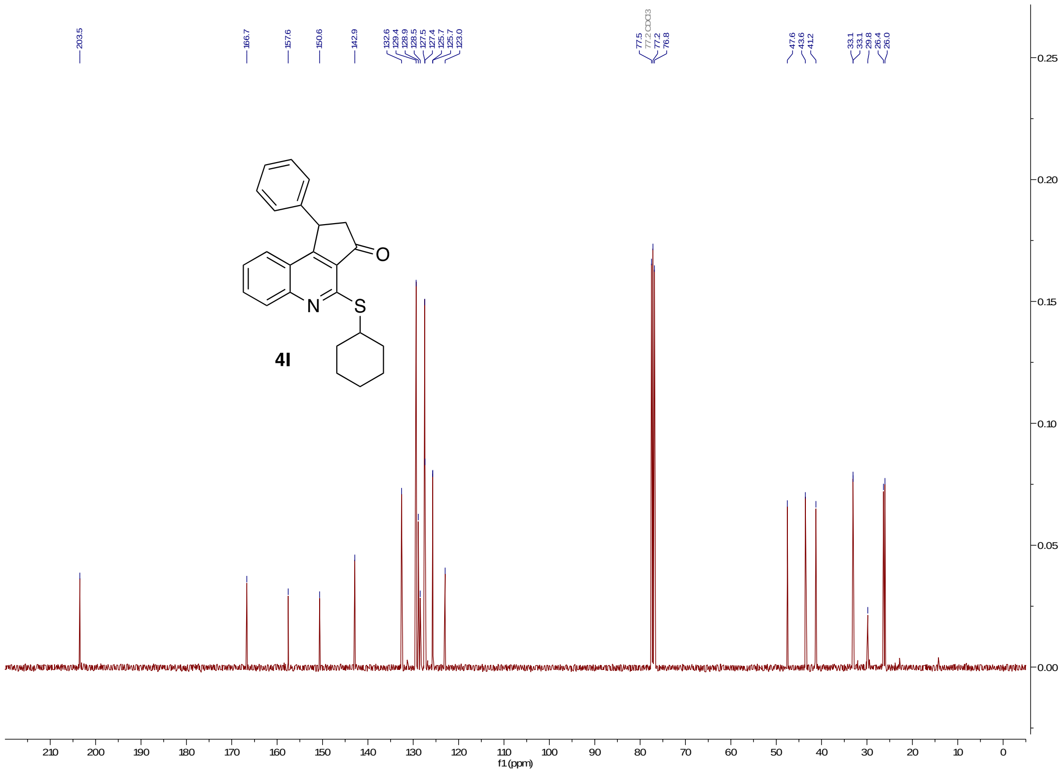

^{1}H NMR spectrum of **4-(Dodecylthio)-1-phenyl-1,2-dihydro-3*H*-cyclopenta[*c*]quinolin-3-one (4m)** (400 MHz, $CDCl_3$)

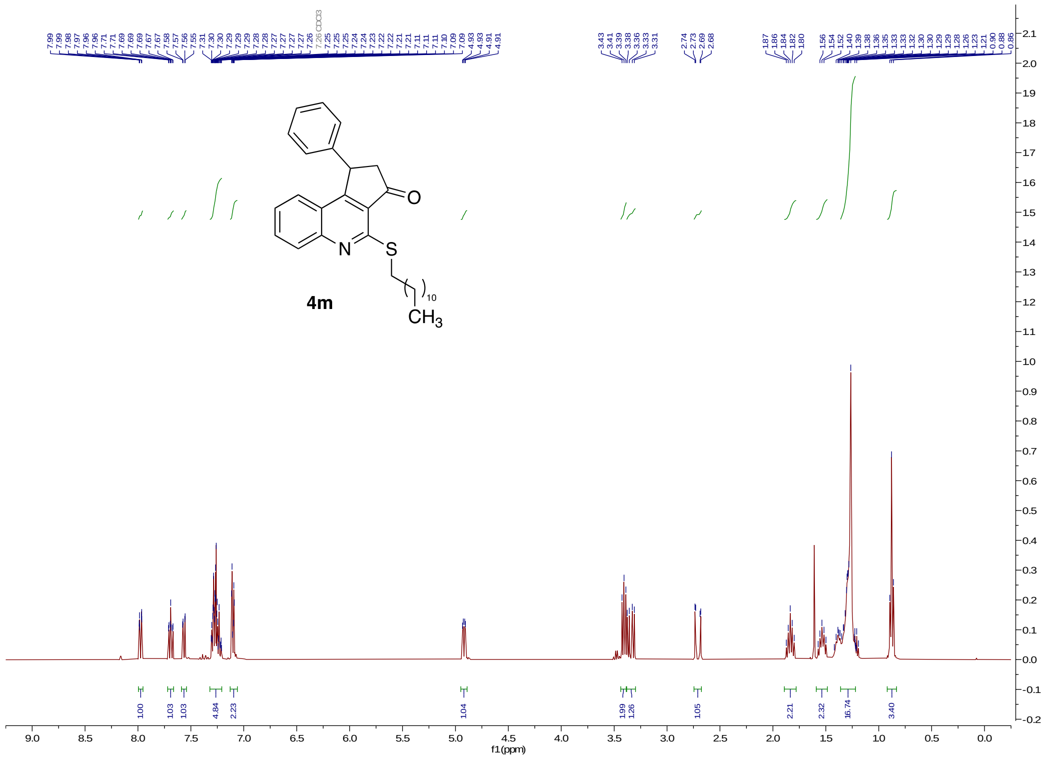

^{13}C NMR spectrum of **4-(Dodecylthio)-1-phenyl-1,2-dihydro-3*H*-cyclopenta[*c*]quinolin-3-one (4m)** (100 MHz, $CDCl_3$)

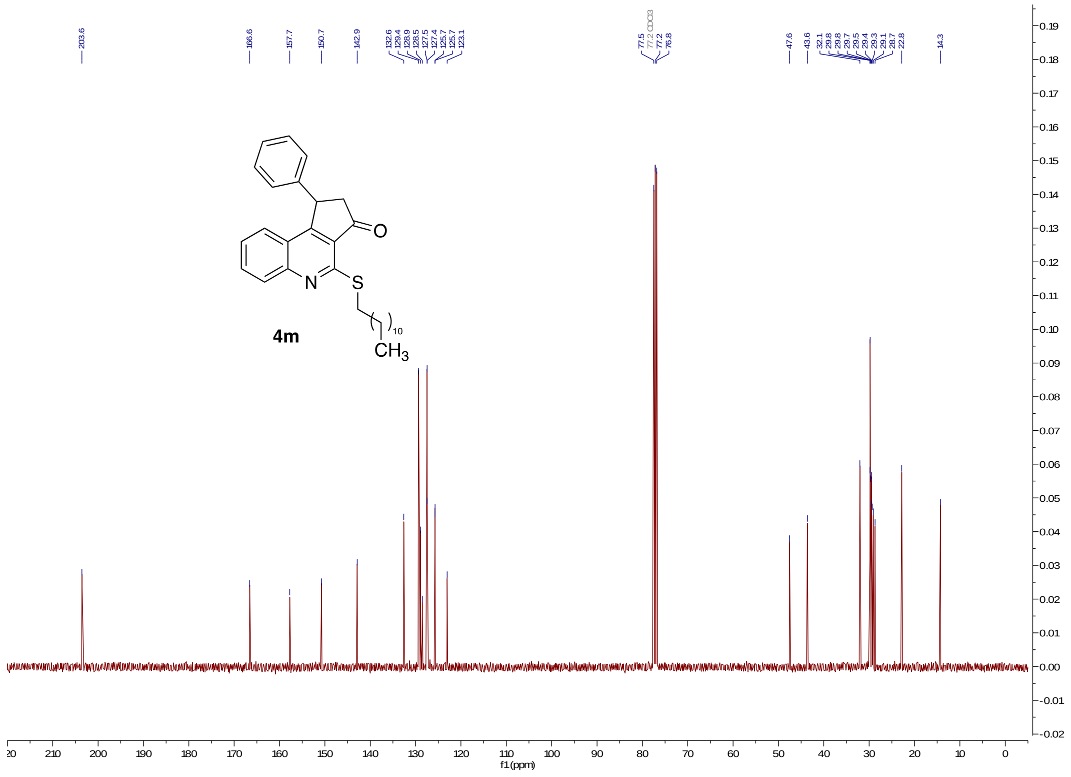

^{1}H NMR spectrum of **4-(Benzylthio)-1-phenyl-1,2-dihydro-3*H*-cyclopenta[*c*]quinolin-3-one (4n)** (400 MHz, $CDCl_3$)

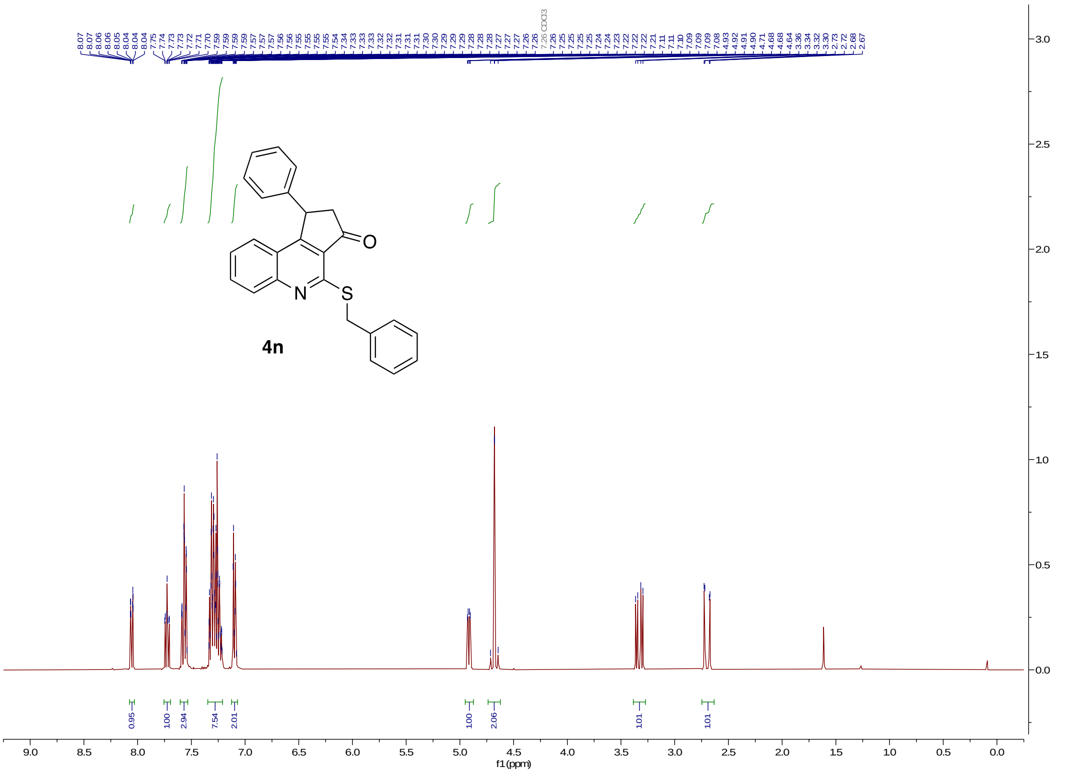

^{13}C NMR spectrum of **4-(Benzylthio)-1-phenyl-1,2-dihydro-3*H*-cyclopenta[*c*]quinolin-3-one (4n)** (100 MHz, $CDCl_3$)

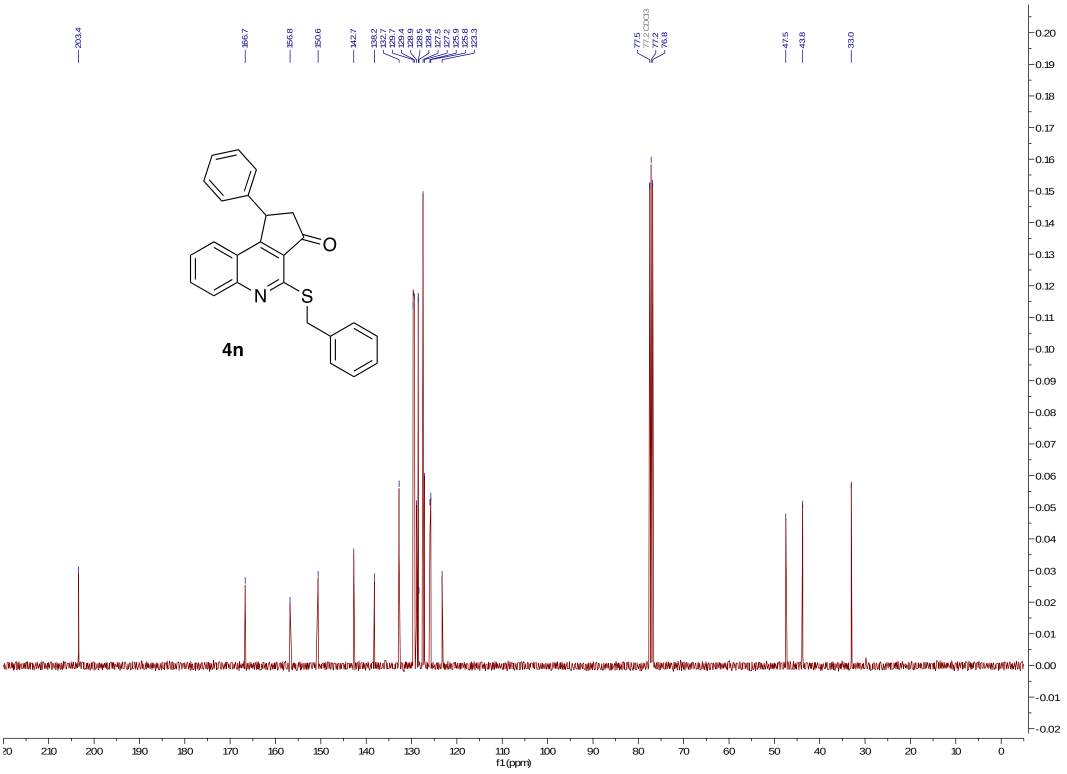

^{1}H NMR spectrum of **1-Phenyl-4-(phenylselanyl)-1,2-dihydro-3*H*-cyclopenta[*c*]quinolin-3-one (4b$_{Se}$)** (400 MHz, $CDCl_3$)

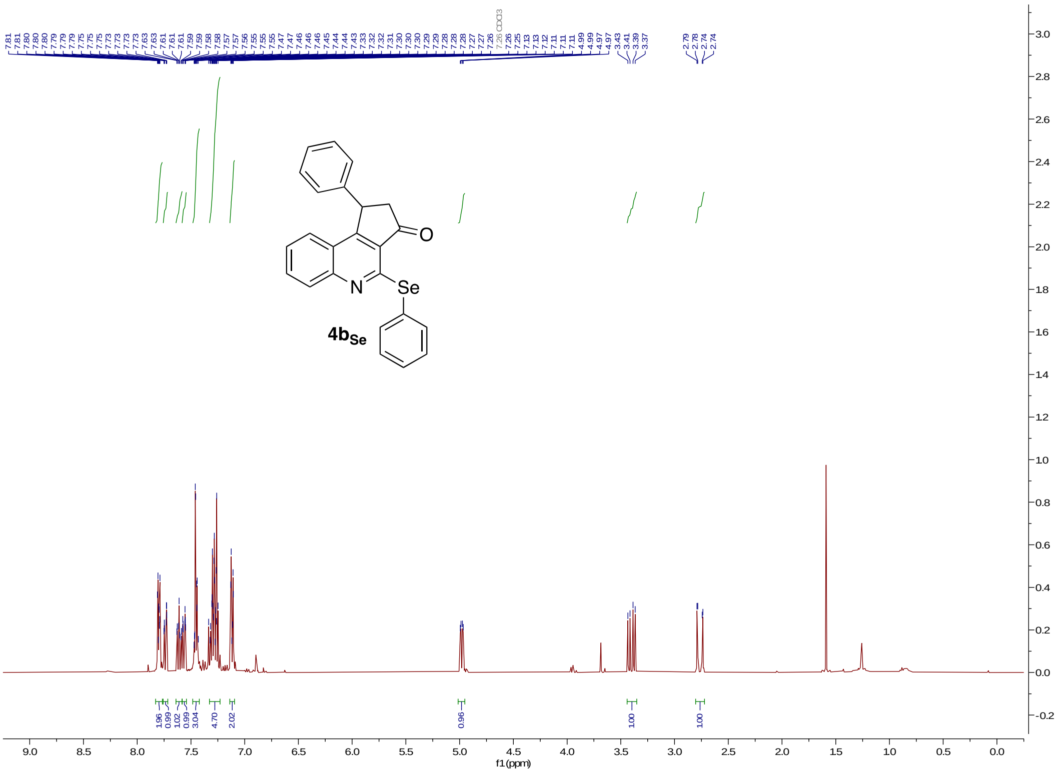

^{13}C NMR spectrum of **1-Phenyl-4-(phenylselanyl)-1,2-dihydro-3*H*-cyclopenta[*c*]quinolin-3-one (4b$_{Se}$)** (100 MHz, $CDCl_3$)

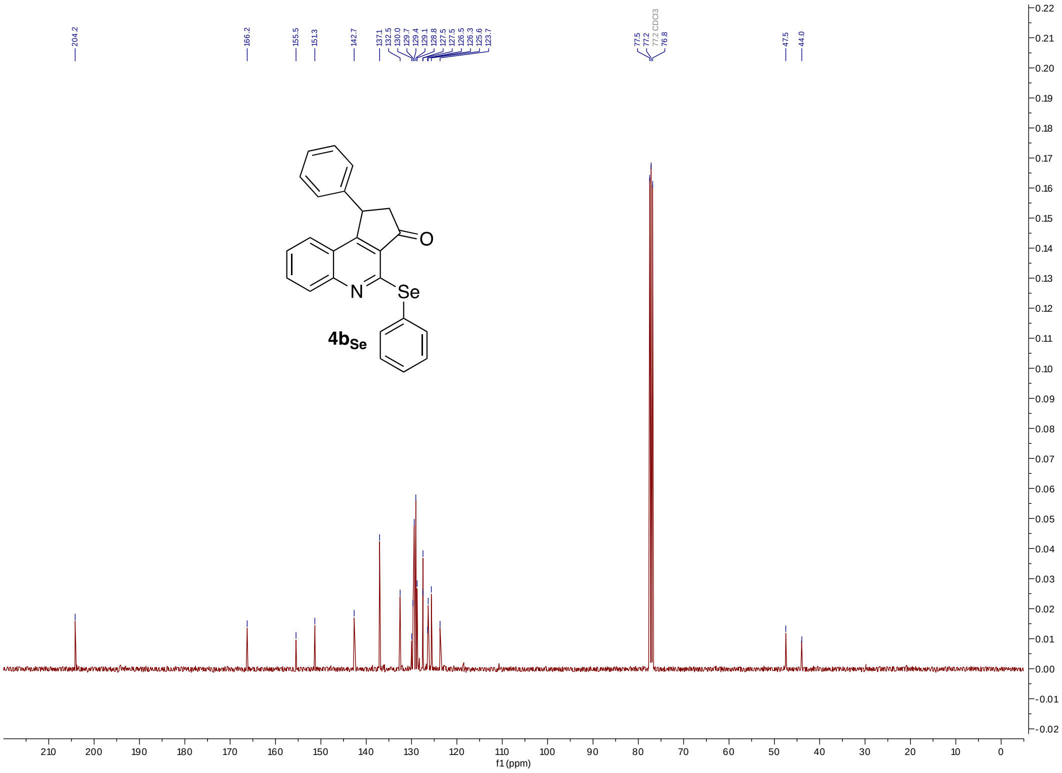

^{1}H NMR spectrum of **1-(4-Methoxyphenyl)-4-(propylthio)-1,2-dihydro-3*H*-cyclopenta[*c*]quinolin-3-one (4o)** (400 MHz, $CDCl_3$)

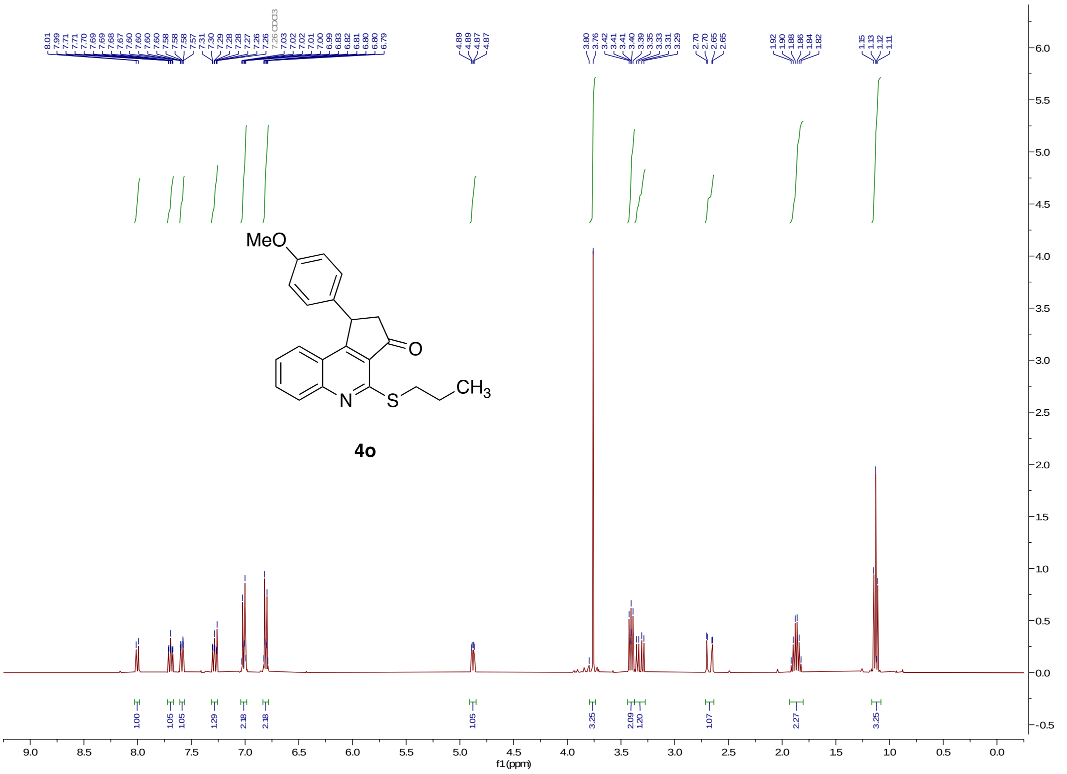

^{13}C NMR spectrum of **1-(4-Methoxyphenyl)-4-(propylthio)-1,2-dihydro-3*H*-cyclopenta[*c*]quinolin-3-one (4o)** (100 MHz, $CDCl_3$)

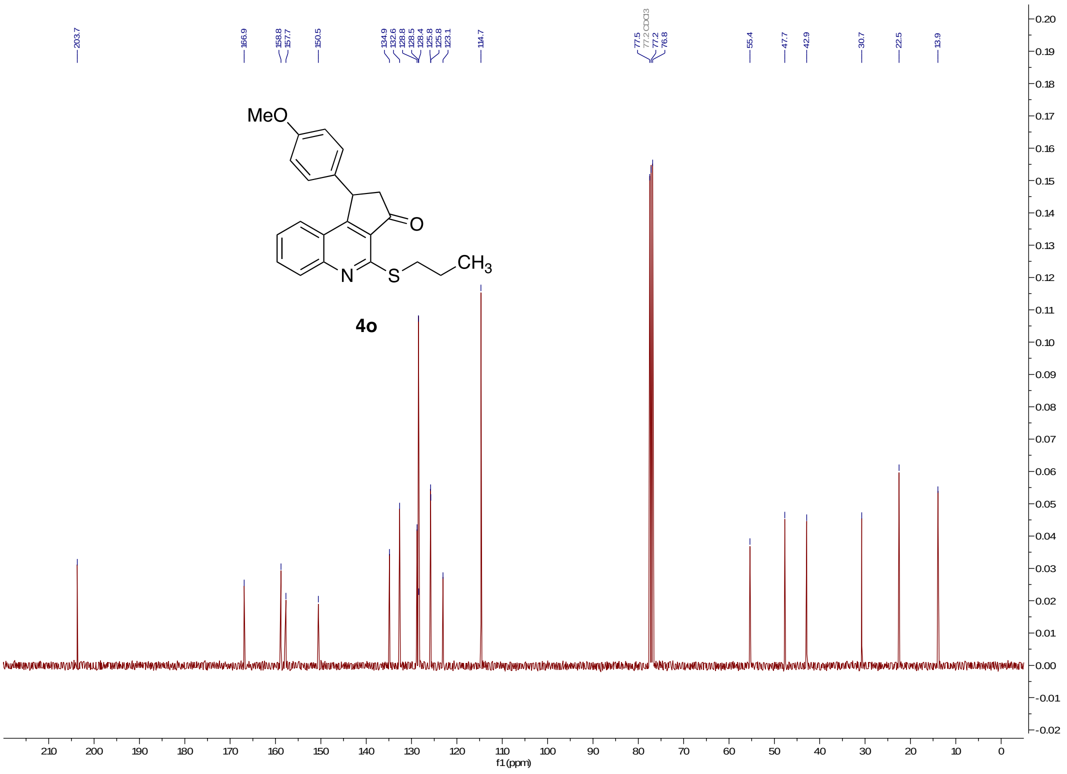

^{1}H NMR spectrum of **1-(4-Methoxyphenyl)-4-(*p*-tolylthio)-1,2-dihydro-3*H*-cyclopenta[*c*]quinolin-3-one (4p)** (400 MHz, $CDCl_3$)

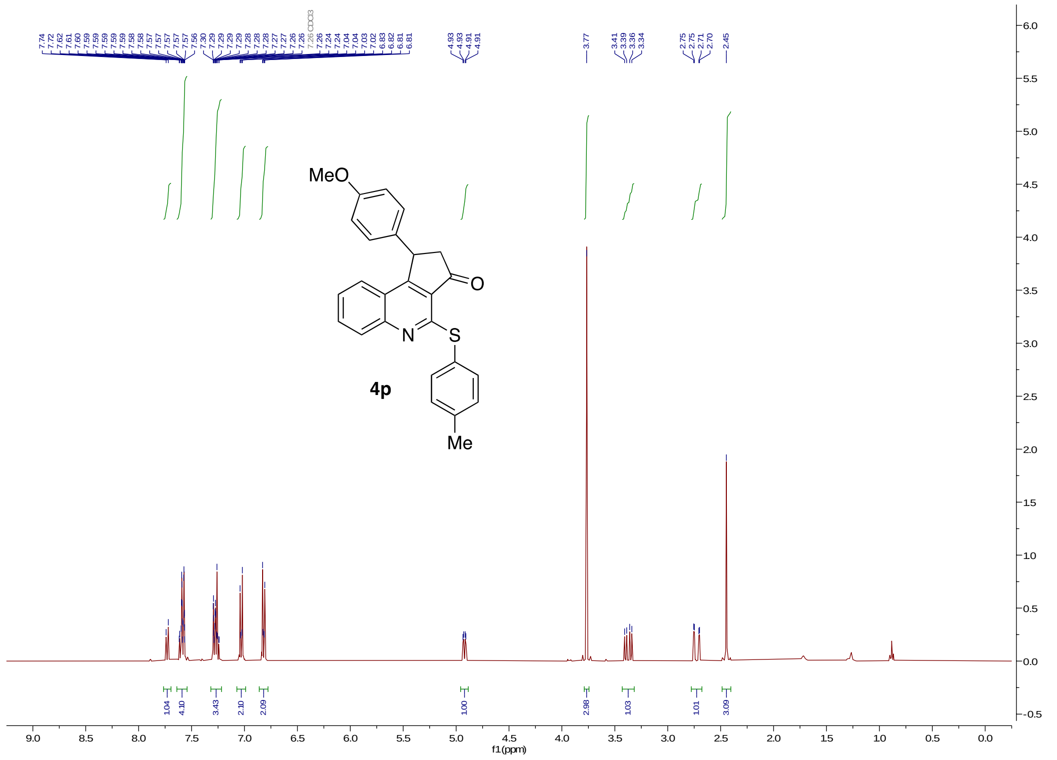

^{13}C NMR spectrum of **1-(4-Methoxyphenyl)-4-(*p*-tolylthio)-1,2-dihydro-3*H*-cyclopenta[*c*]quinolin-3-one (4p)** (100 MHz, $CDCl_3$)

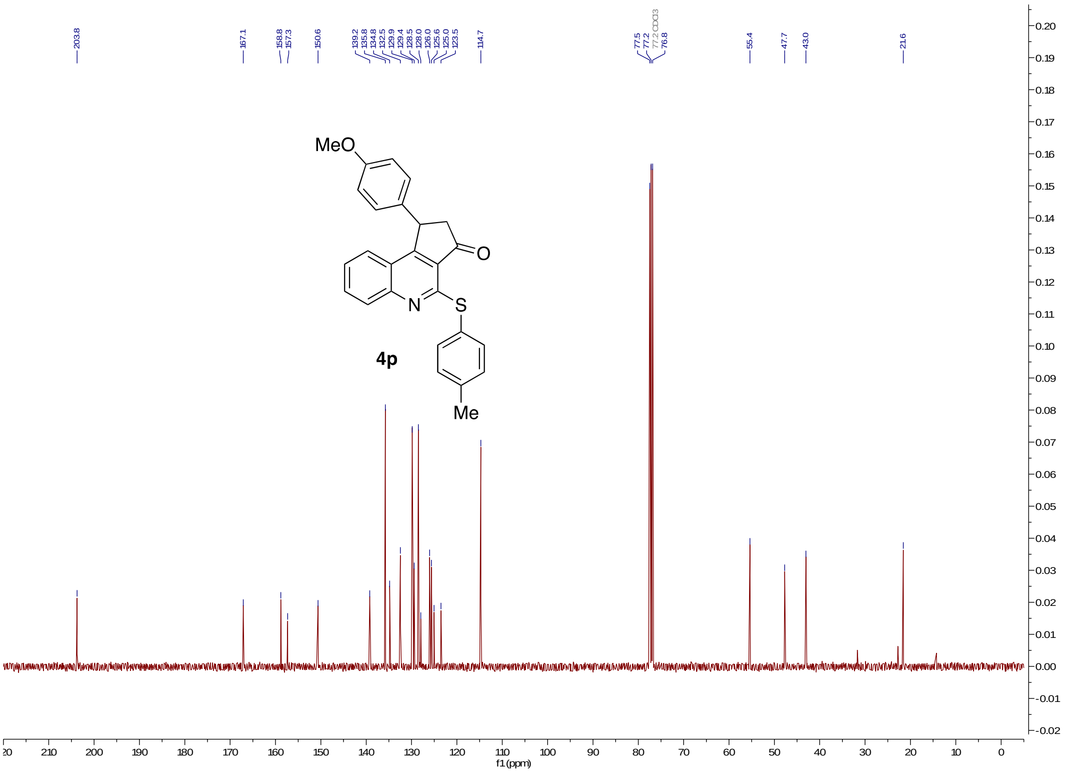

^{1}H NMR spectrum of **1-(4-Fluorophenyl)-4-(propylthio)-1,2-dihydro-3*H*-cyclopenta[*c*]quinolin-3-one (4q)** (400 MHz, $CDCl_3$)

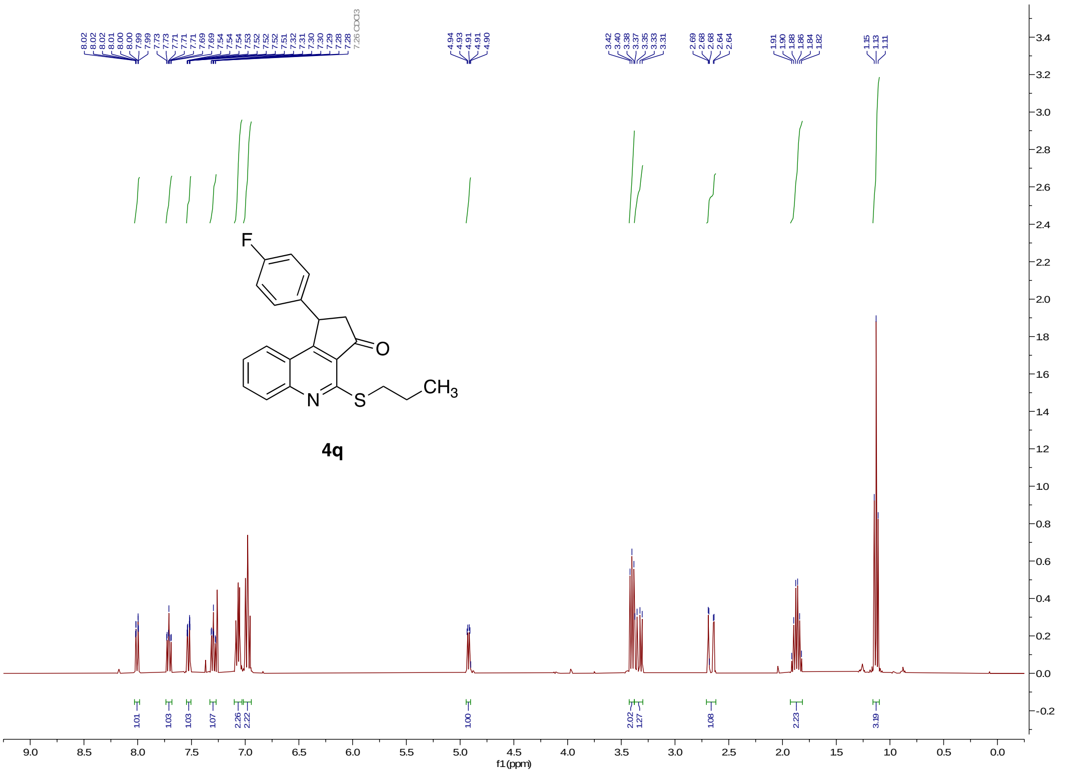

^{13}C NMR spectrum of **1-(4-Fluorophenyl)-4-(propylthio)-1,2-dihydro-3*H*-cyclopenta[*c*]quinolin-3-one (4q)** (100 MHz, $CDCl_3$)

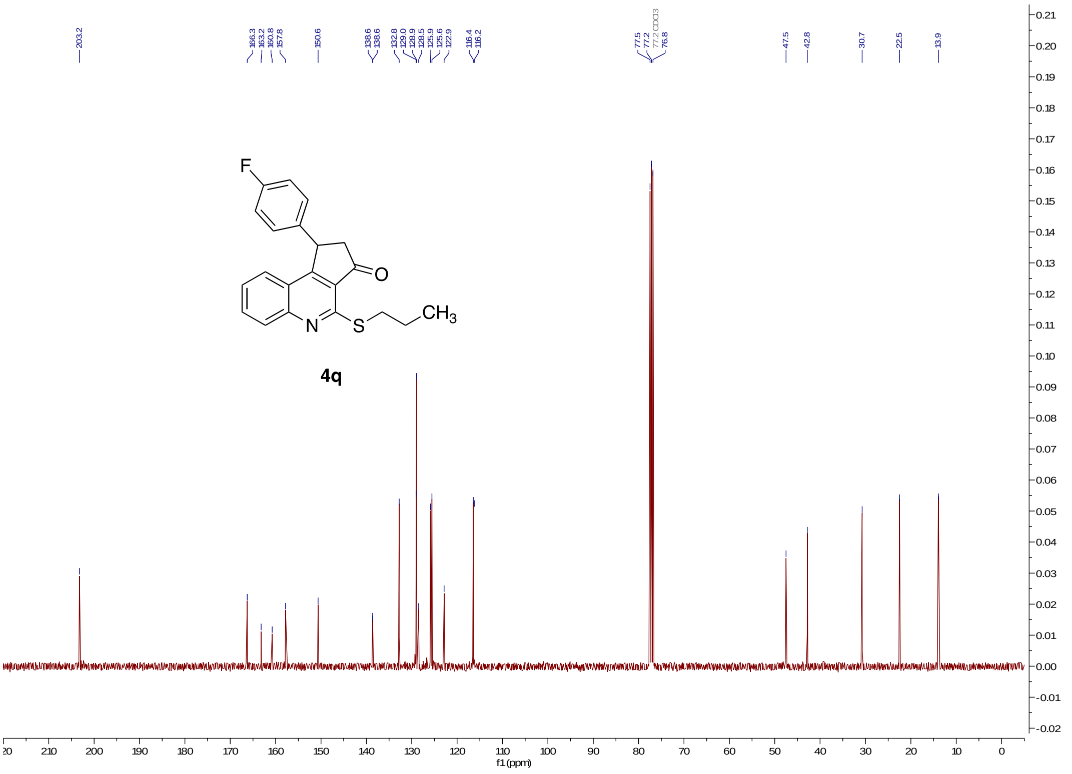

^{19}F NMR spectrum of **1-(4-Fluorophenyl)-4-(propylthio)-1,2-dihydro-3*H*-cyclopenta[*c*]quinolin-3-one (4q)** (376 MHz, $CDCl_3$)

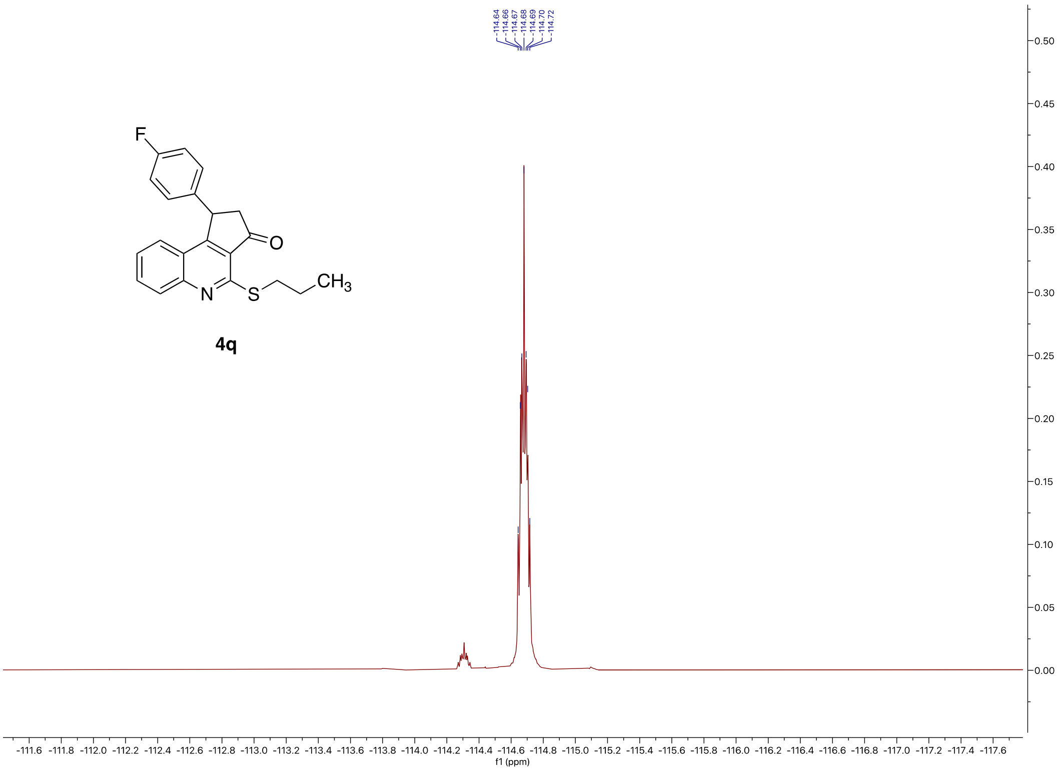

^{1}H NMR spectrum of **1-(4-Fluorophenyl)-4-(*p*-tolylthio)-1,2-dihydro-3*H*-cyclopenta[*c*]quinolin-3-one (4r)** (400 MHz, $CDCl_3$)

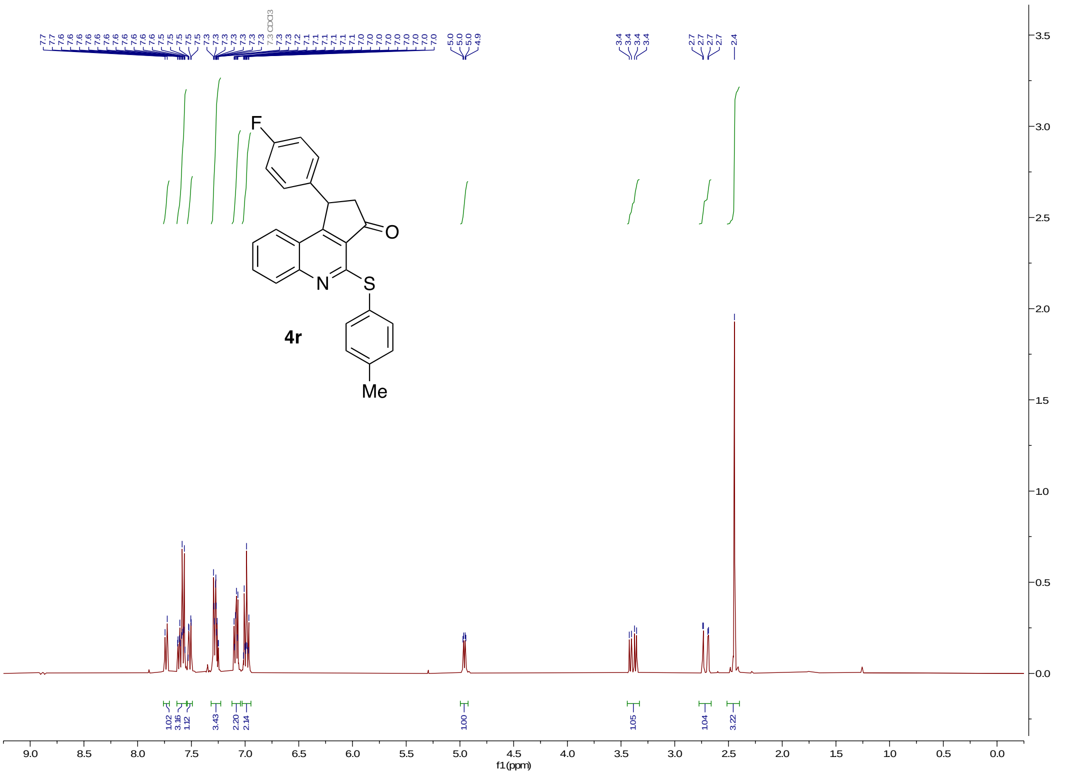

^{13}C NMR spectrum of **1-(4-Fluorophenyl)-4-(*p*-tolylthio)-1,2-dihydro-3*H*-cyclopenta[*c*]quinolin-3-one (4r)** (100 MHz, $CDCl_3$)

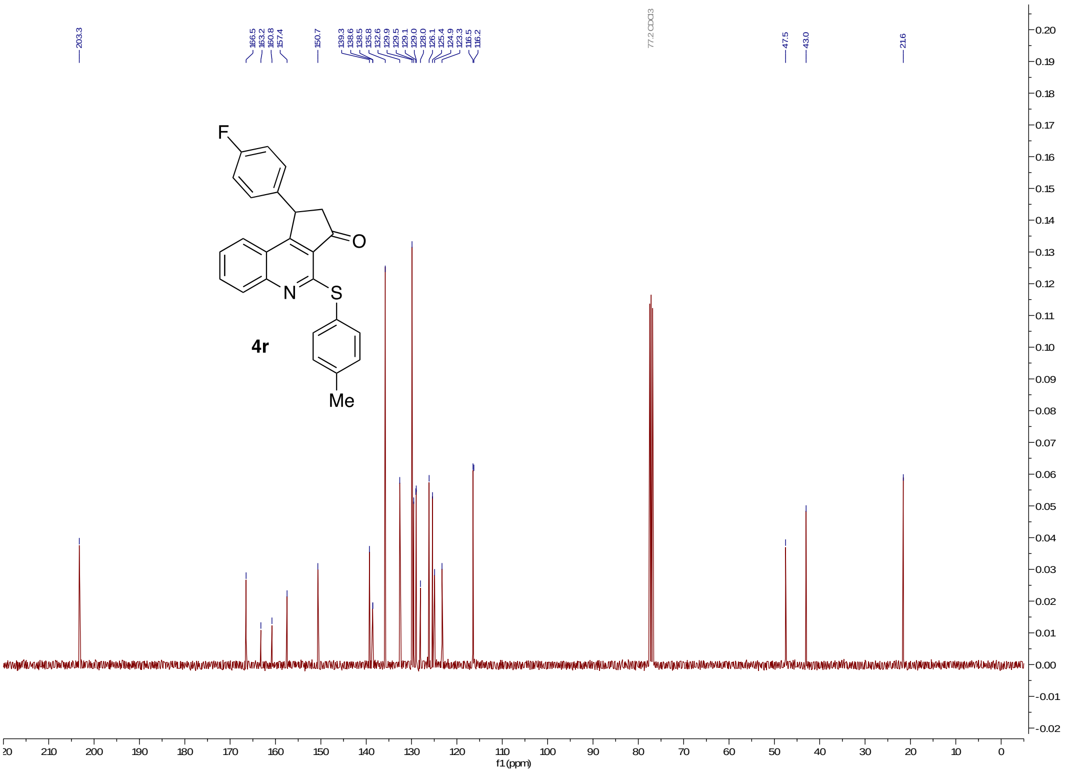

^{19}F NMR spectrum of of **1-(4-Fluorophenyl)-4-(*p*-tolylthio)-1,2-dihydro-3*H*-cyclopenta[*c*]quinolin-3-one (4r)** (376 MHz, $CDCl_3$)

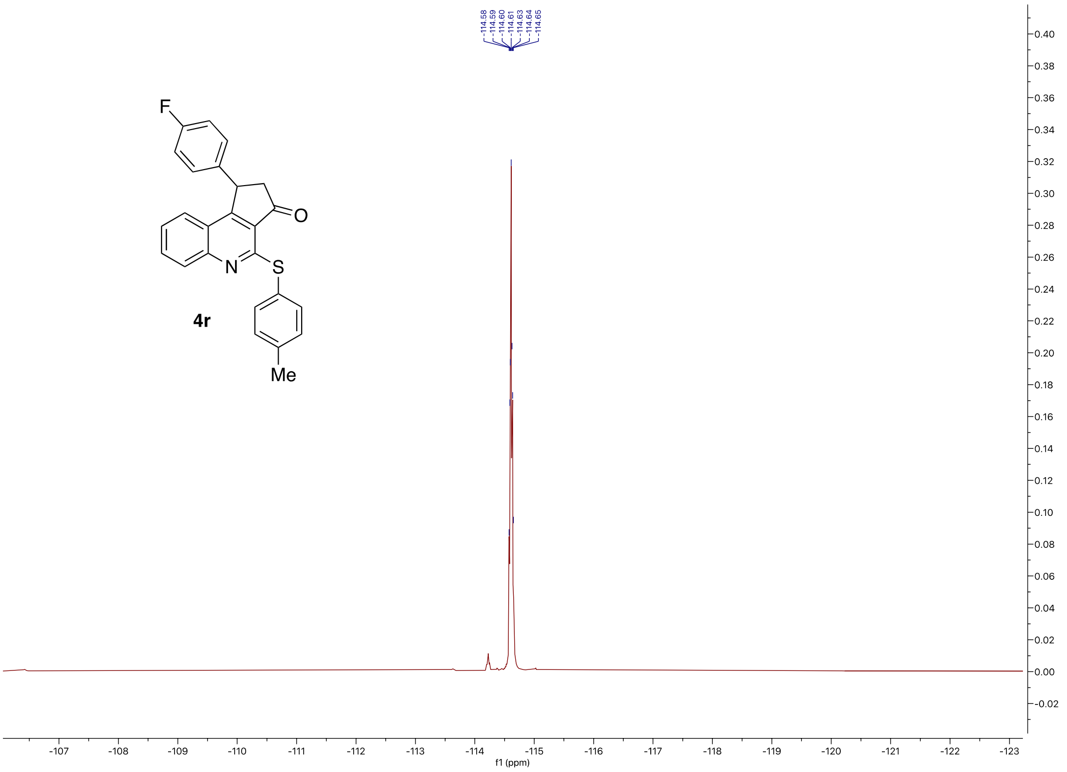

^{1}H NMR spectrum of **1-Cyclopropyl-4-(propylthio)-1,2-dihydro-3*H*-cyclopenta[*c*]quinolin-3-one (4s)** (400 MHz, $CDCl_3$)

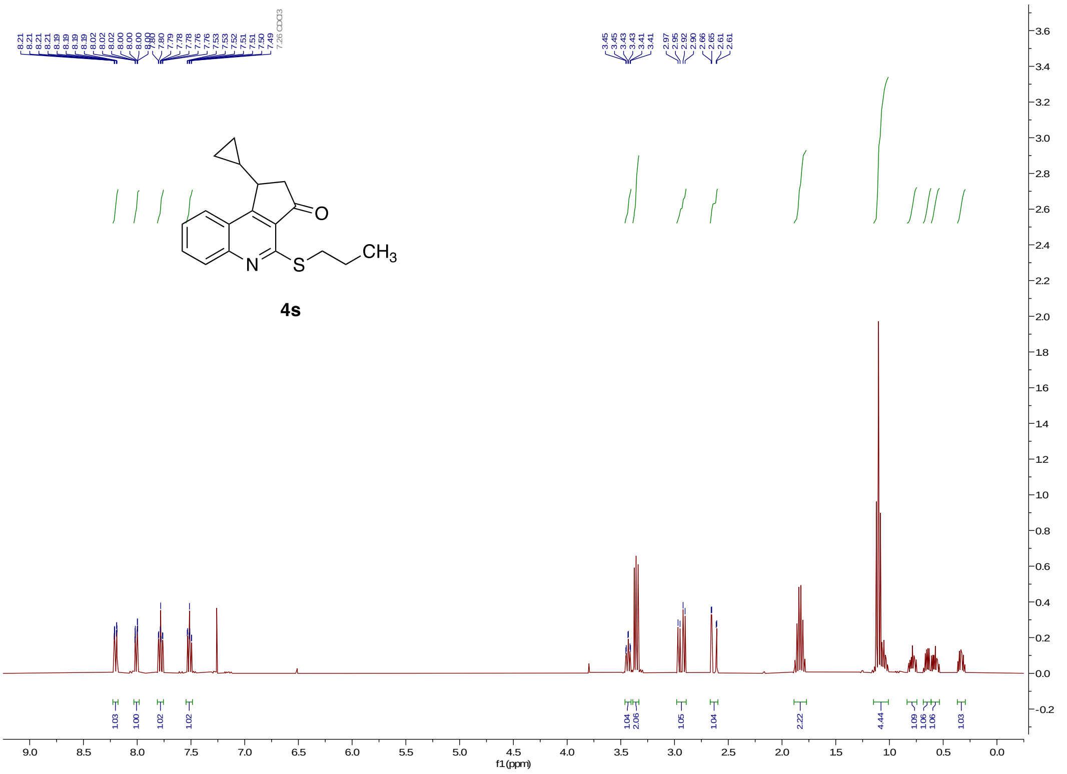

^{13}C NMR spectrum of **1-Cyclopropyl-4-(propylthio)-1,2-dihydro-3*H*-cyclopenta[*c*]quinolin-3-one (4s)** (100 MHz, $CDCl_3$)

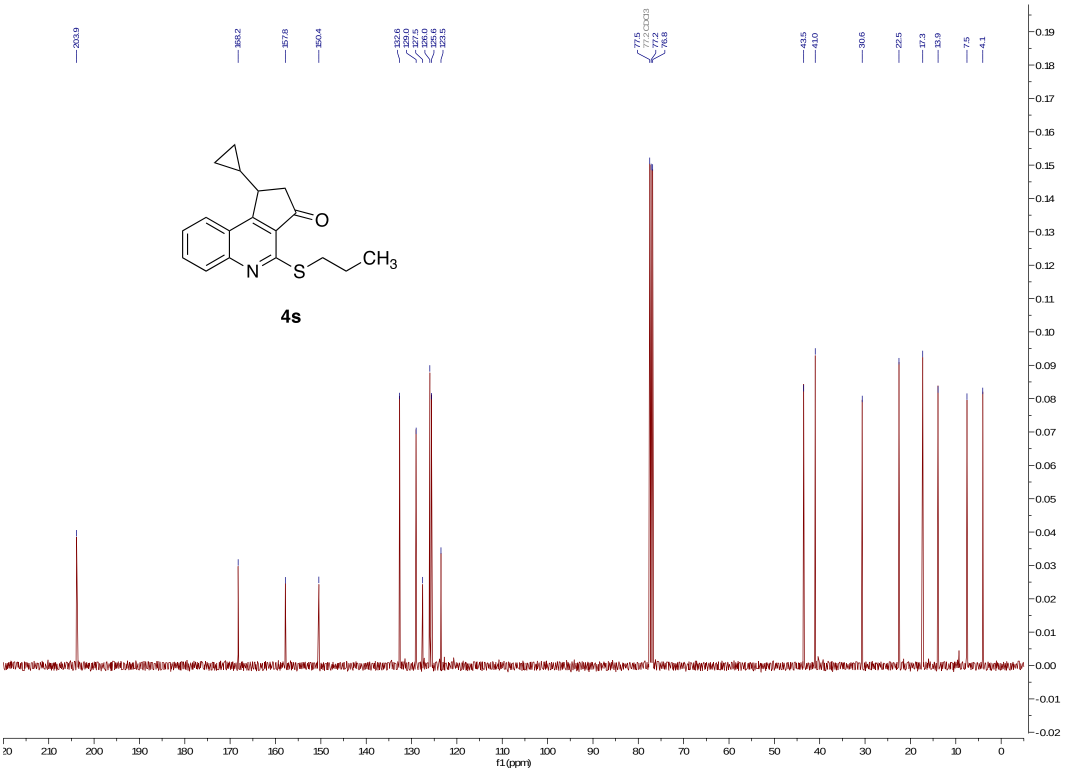

^{1}H NMR spectrum of **1-Cyclopropyl-4-(*p*-tolylthio)-1,2-dihydro-3*H*-cyclopenta[*c*]quinolin-3-one (4t)** (400 MHz, $CDCl_3$)

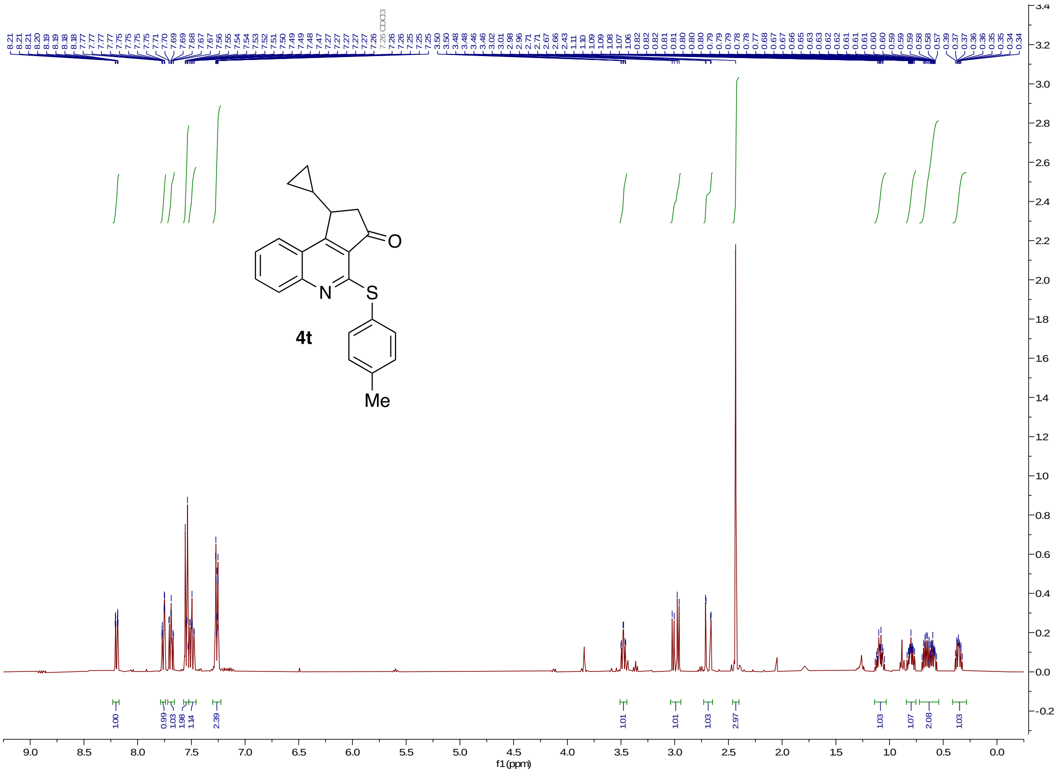

^{13}C NMR spectrum of **1-Cyclopropyl-4-(*p*-tolylthio)-1,2-dihydro-3*H*-cyclopenta[*c*]quinolin-3-one (4t)** (100 MHz, $CDCl_3$)

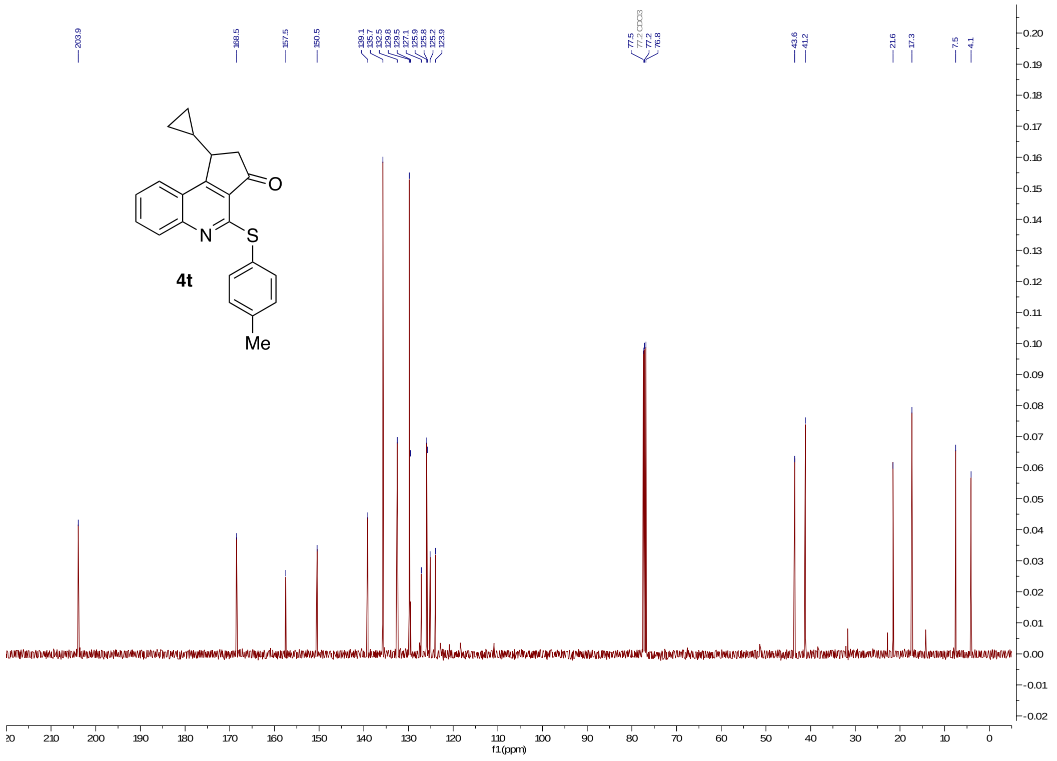

^{1}H NMR spectrum of **8-Bromo-1-phenyl-4-(propylthio)-1,2-dihydro-3*H*-cyclopenta[*c*]quinolin-3-one (4u)** (400 MHz, $CDCl_3$)

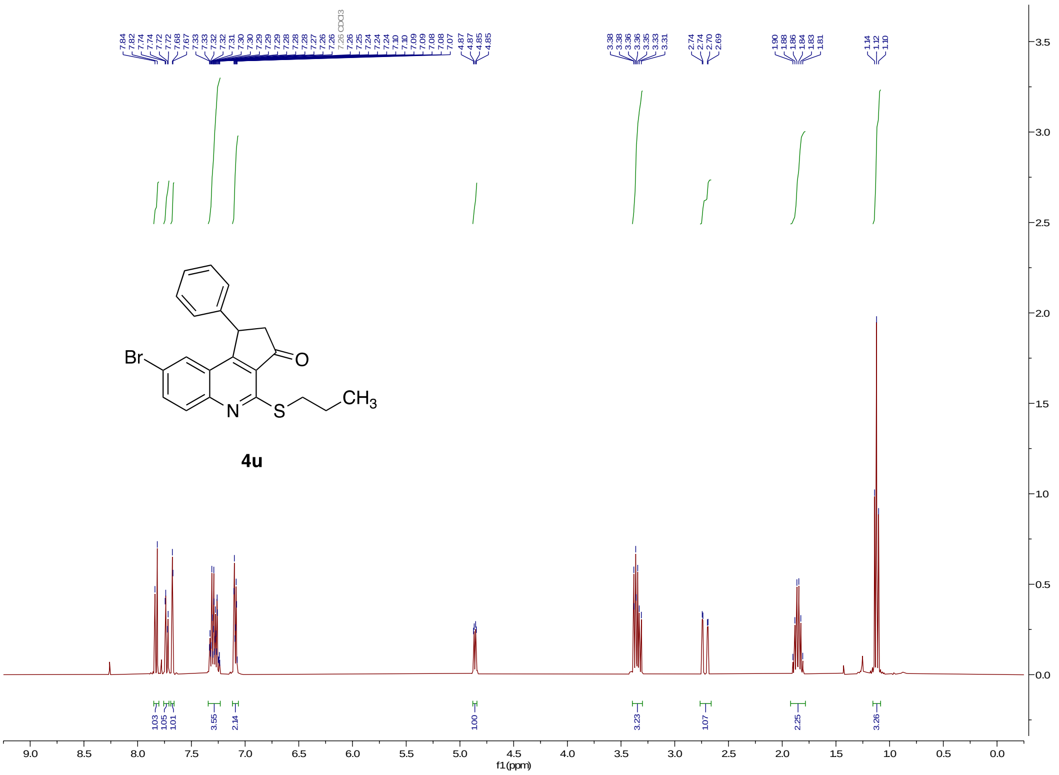

^{13}C NMR spectrum of **8-Bromo-1-phenyl-4-(propylthio)-1,2-dihydro-3*H*-cyclopenta[*c*]quinolin-3-one (4u)** (100 MHz, $CDCl_3$)

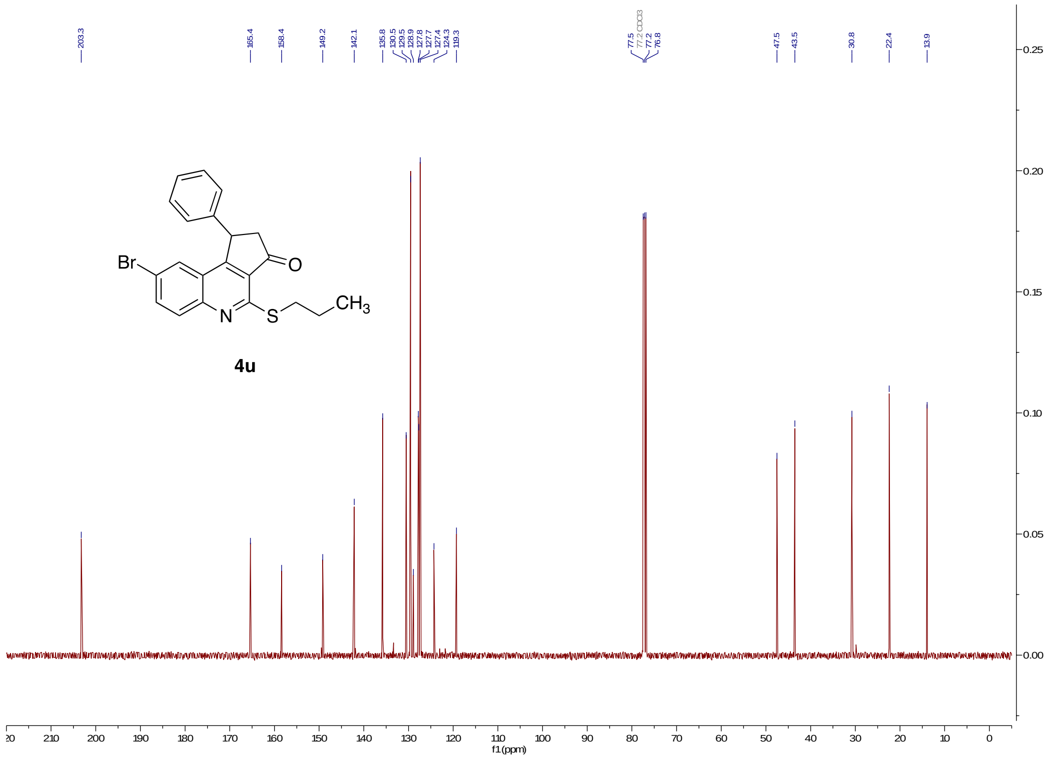

^{1}H NMR spectrum of **8-Bromo-1-phenyl-4-(*p*-tolylthio)-1,2-dihydro-3*H*-cyclopenta[*c*]quinolin-3-one (4v)** (400 MHz, $CDCl_3$)

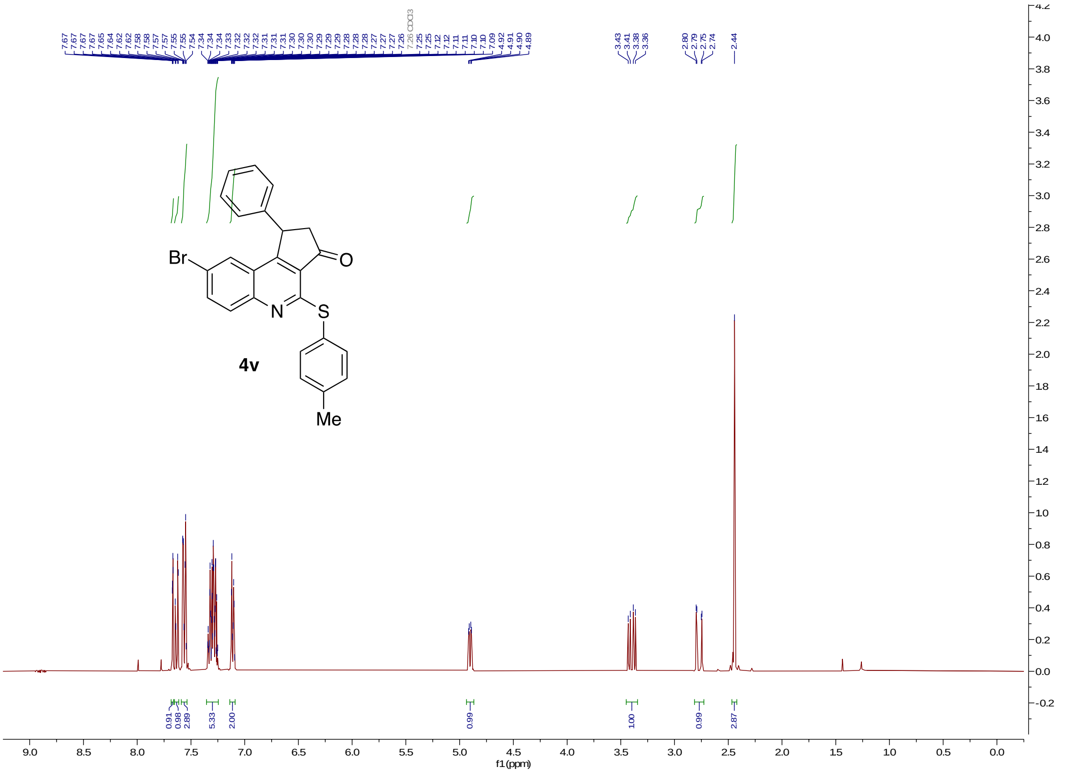

^{13}C NMR spectrum of **8-Bromo-1-phenyl-4-(*p*-tolylthio)-1,2-dihydro-3*H*-cyclopenta[*c*]quinolin-3-one (4v)** (100 MHz, $CDCl_3$)

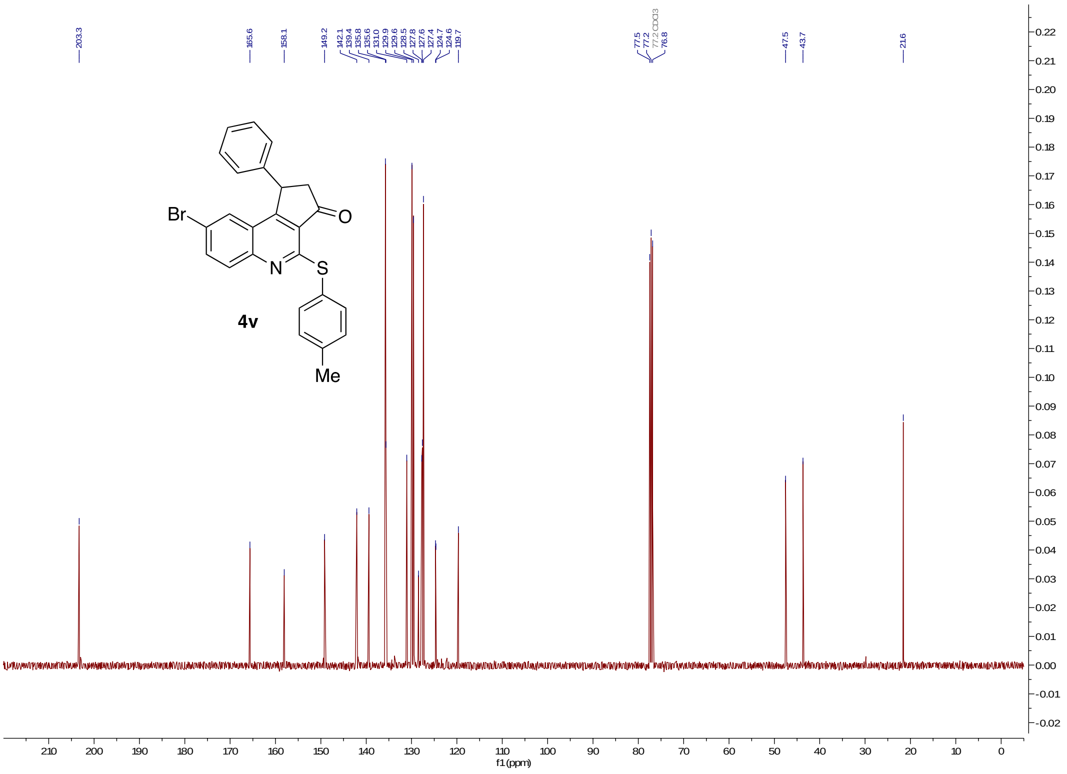

^{1}H NMR spectrum of **2'-Bromo-2-(4-(dimethylamino)phenyl)spiro[cyclopentane-1,3'-indol]-2-en-4-one (5b)** (400 MHz, $CDCl_3$)

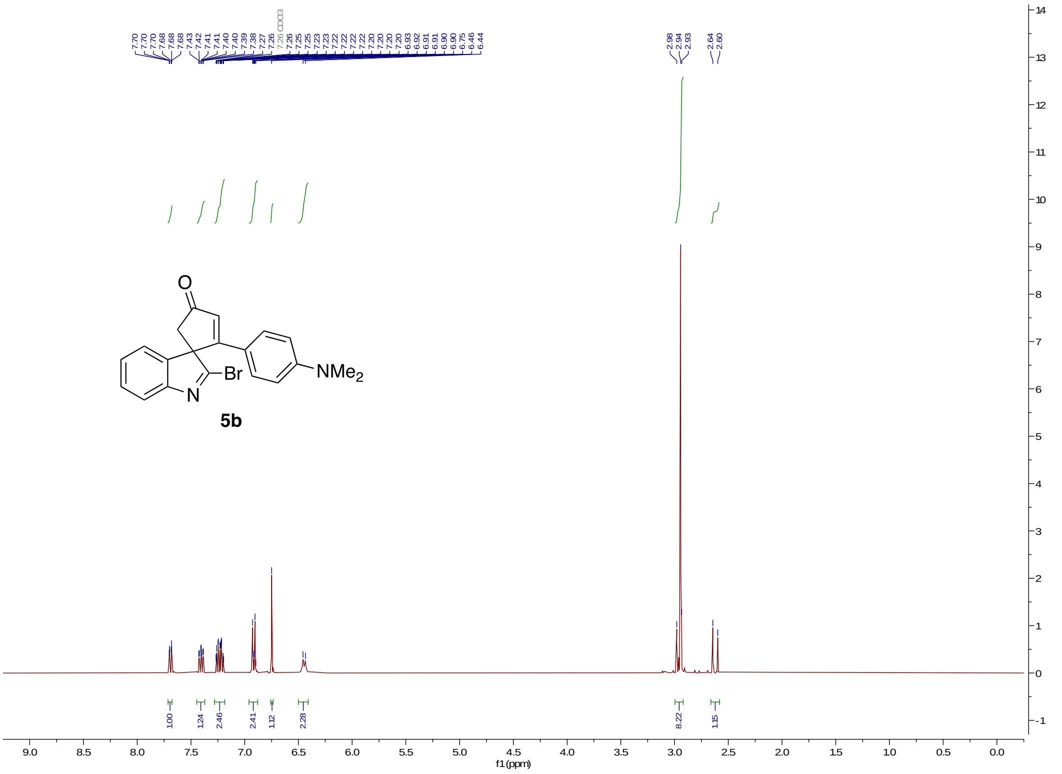

^{13}C NMR spectrum of **2'-Bromo-2-(4-(dimethylamino)phenyl)spiro[cyclopentane-1,3'-indol]-2-en-4-one (5b)** (100 MHz, $CDCl_3$)

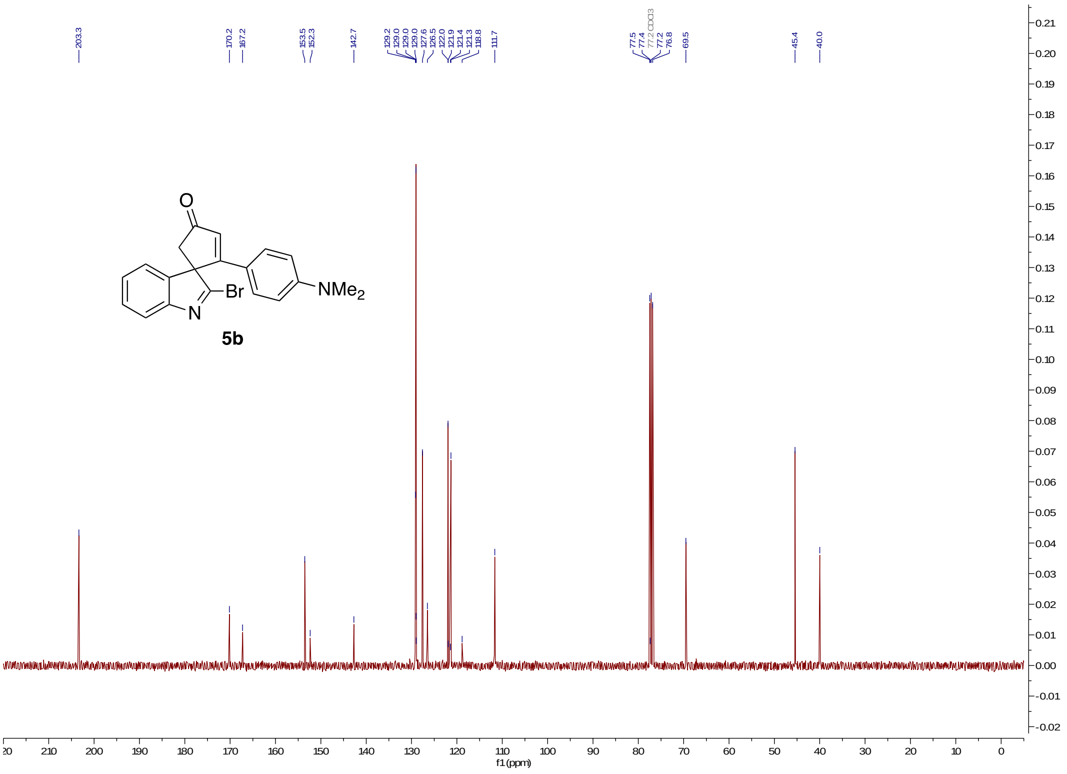

^{1}H NMR spectrum of **2-Phenylspiro[cyclopentane-1,3'-indolin]-2-ene-2',4-dione (7a)** (400 MHz, $CDCl_3$)

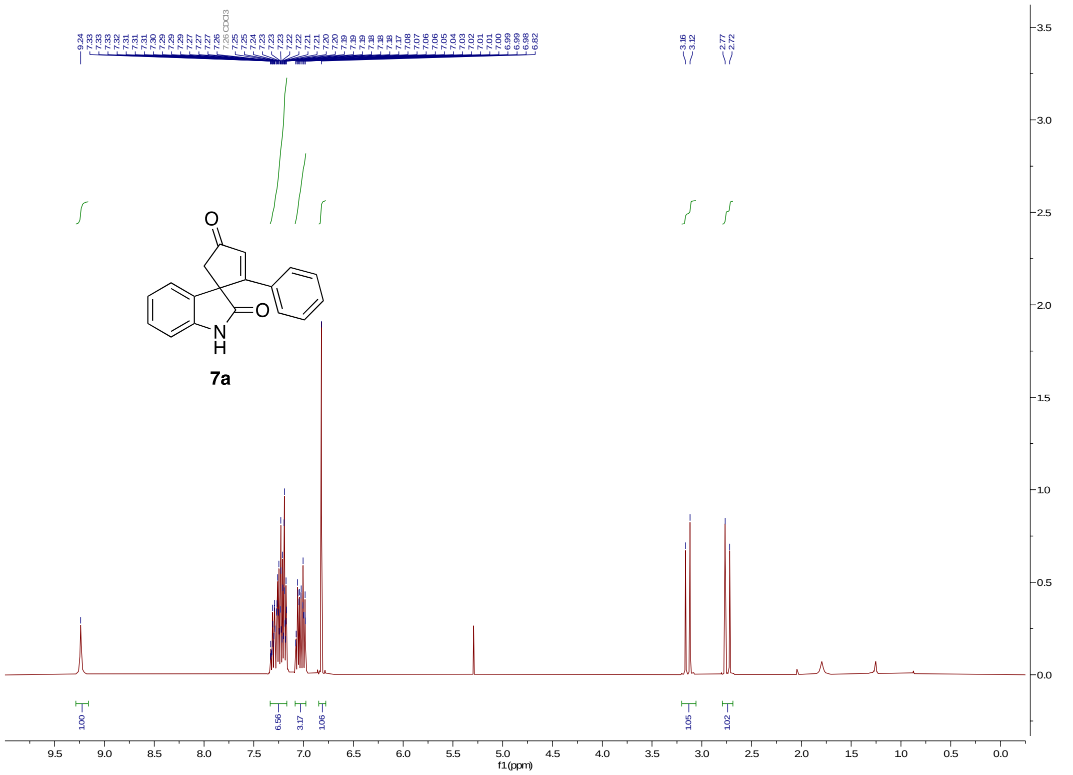

^{13}C NMR spectrum of **2-Phenylspiro[cyclopentane-1,3'-indolin]-2-ene-2',4-dione (7a)** (100 MHz, $CDCl_3$)

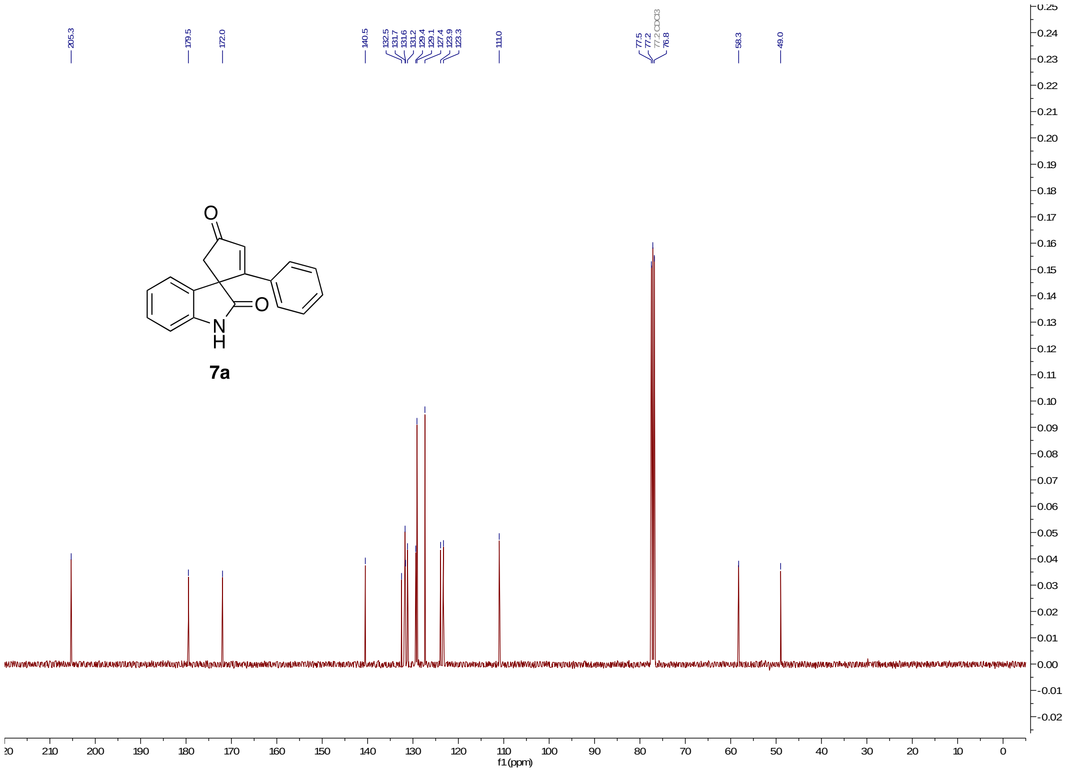

^{1}H NMR spectrum of **4-Bromo-1-phenyl-1,2-dihydro-3*H*-cyclopenta[*c*]quinolin-3-one (8)** (400 MHz, $CDCl_3$)

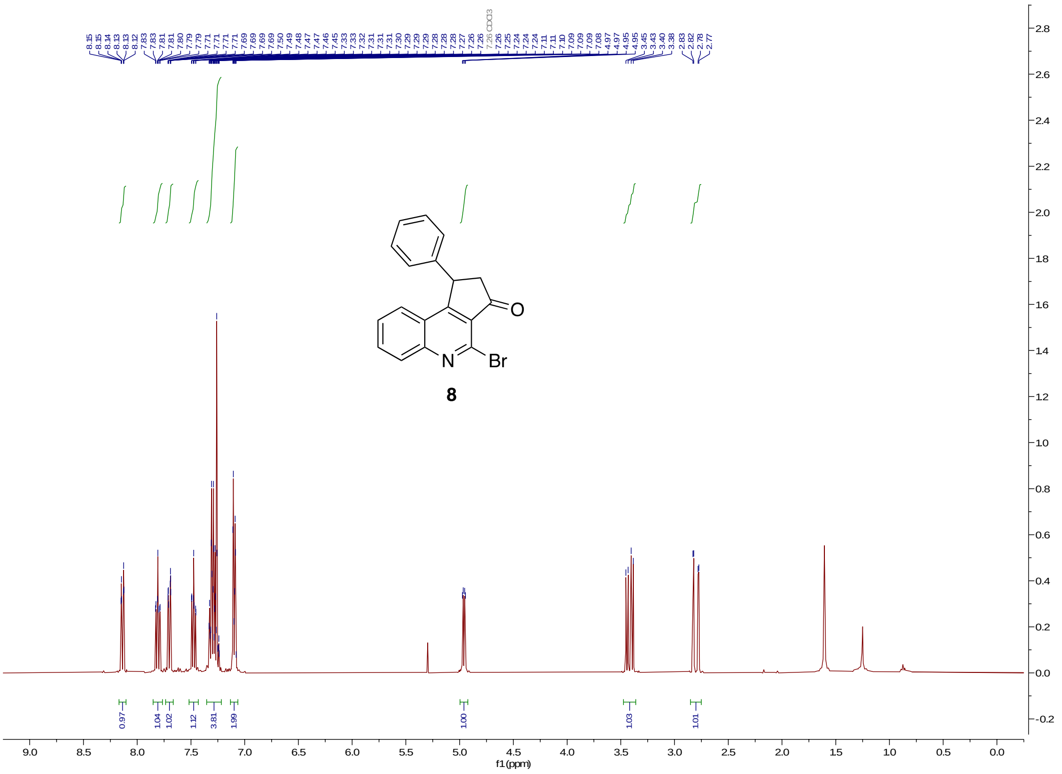

^{13}C NMR spectrum of **4-Bromo-1-phenyl-1,2-dihydro-3*H*-cyclopenta[*c*]quinolin-3-one (8)** (100 MHz, $CDCl_3$)

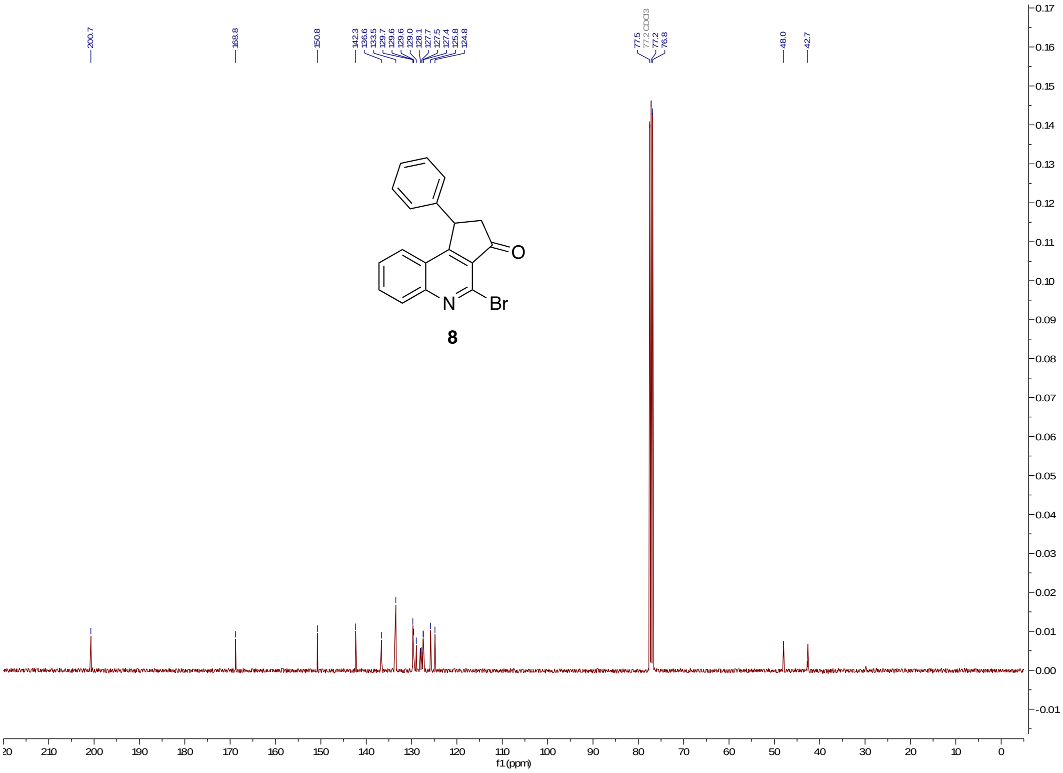

^{1}H NMR spectrum of **2-Phenyl-2'-thioxospiro[cyclopentane-1,3'-indolin]-2-en-4-one (9a)** (400 MHz, $CDCl_3$)

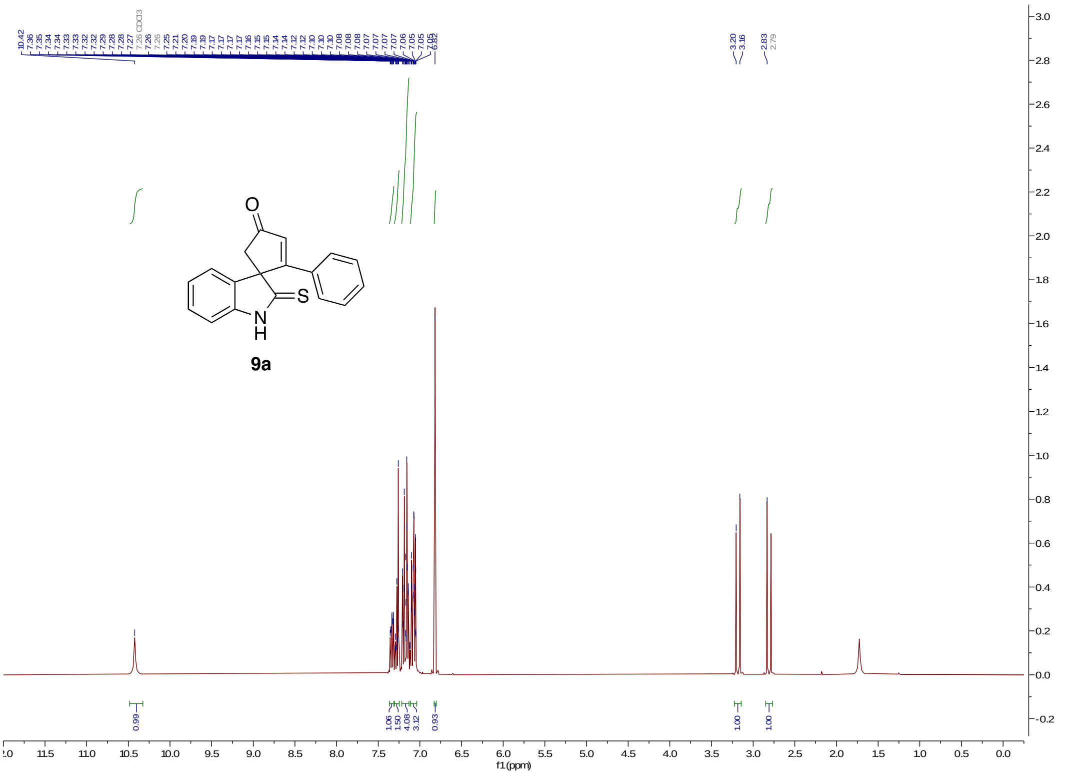

^{13}C NMR spectrum of **2-Phenyl-2'-thioxospiro[cyclopentane-1,3'-indolin]-2-en-4-one (9a)** (100 MHz, $CDCl_3$)

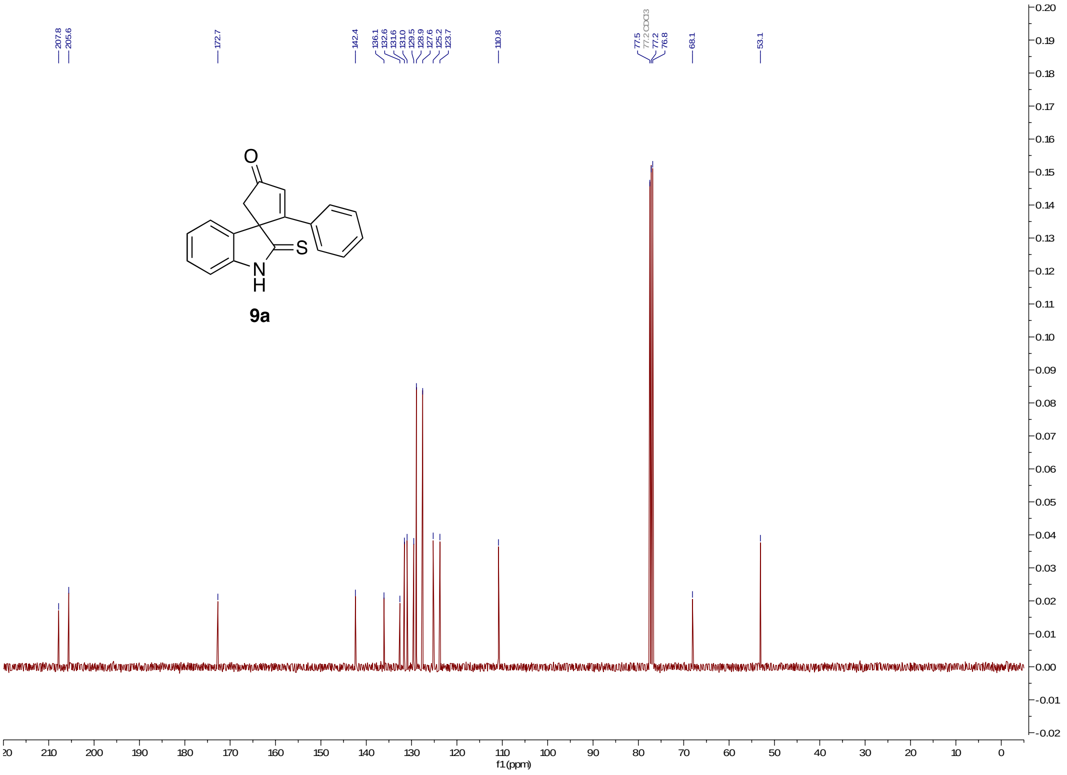

^{1}H NMR spectrum of **2-(4-Methoxyphenyl)-2'-thioxospiro[cyclopentane-1,3'-indolin]-2-en-4-one (9b)** (400 MHz, $CDCl_3$)

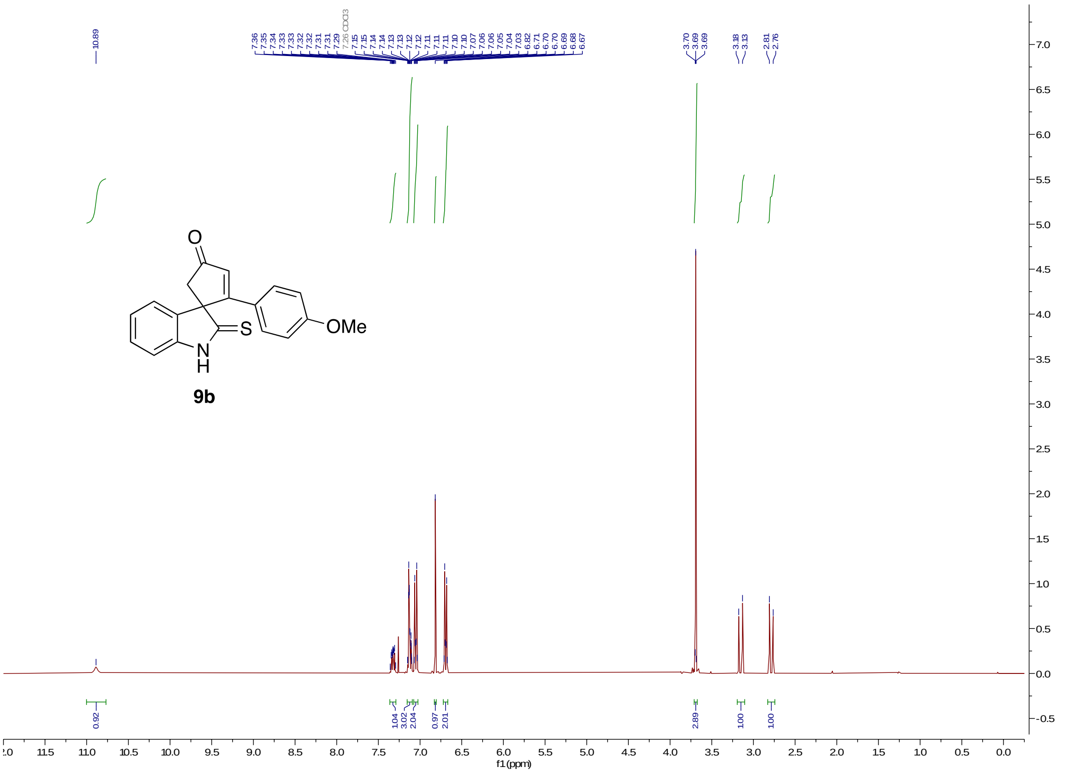

^{13}C NMR spectrum of **2-(4-Methoxyphenyl)-2'-thioxospiro[cyclopentane-1,3'-indolin]-2-en-4-one (9b)** (100 MHz, $CDCl_3$)

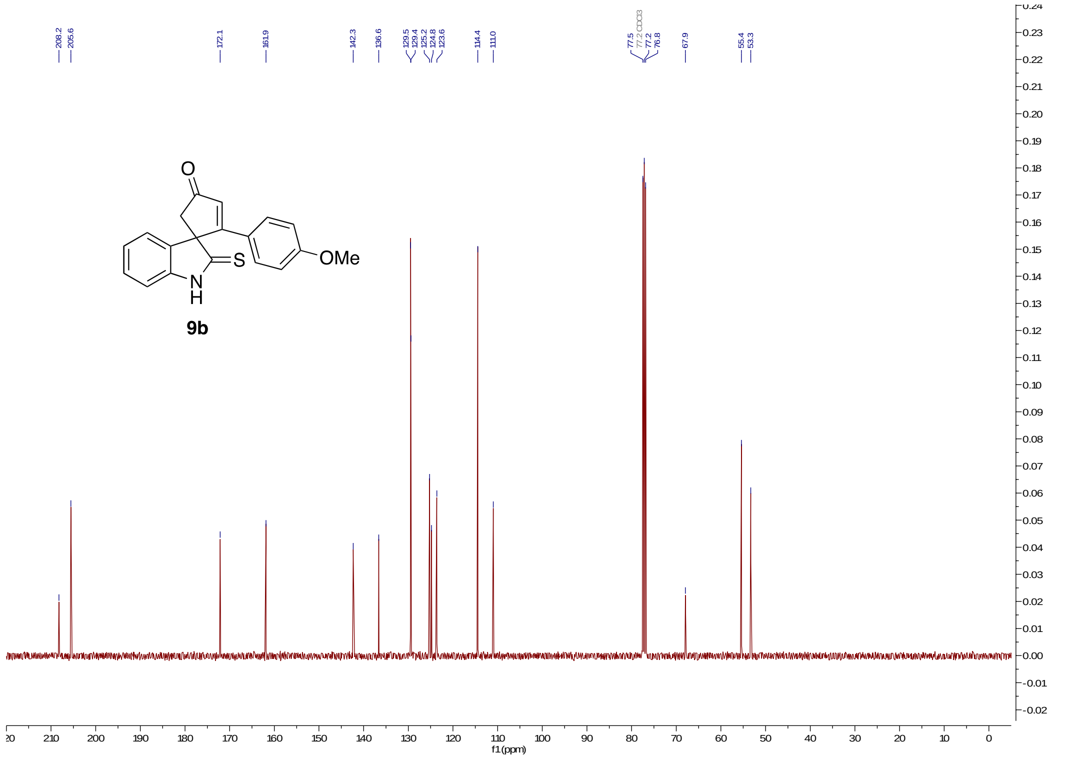

^{1}H NMR spectrum of **2-(4-Fluorophenyl)-2'-thioxospiro[cyclopentane-1,3'-indolin]-2-en-4-one (9c)** (400 MHz, $CDCl_3$)

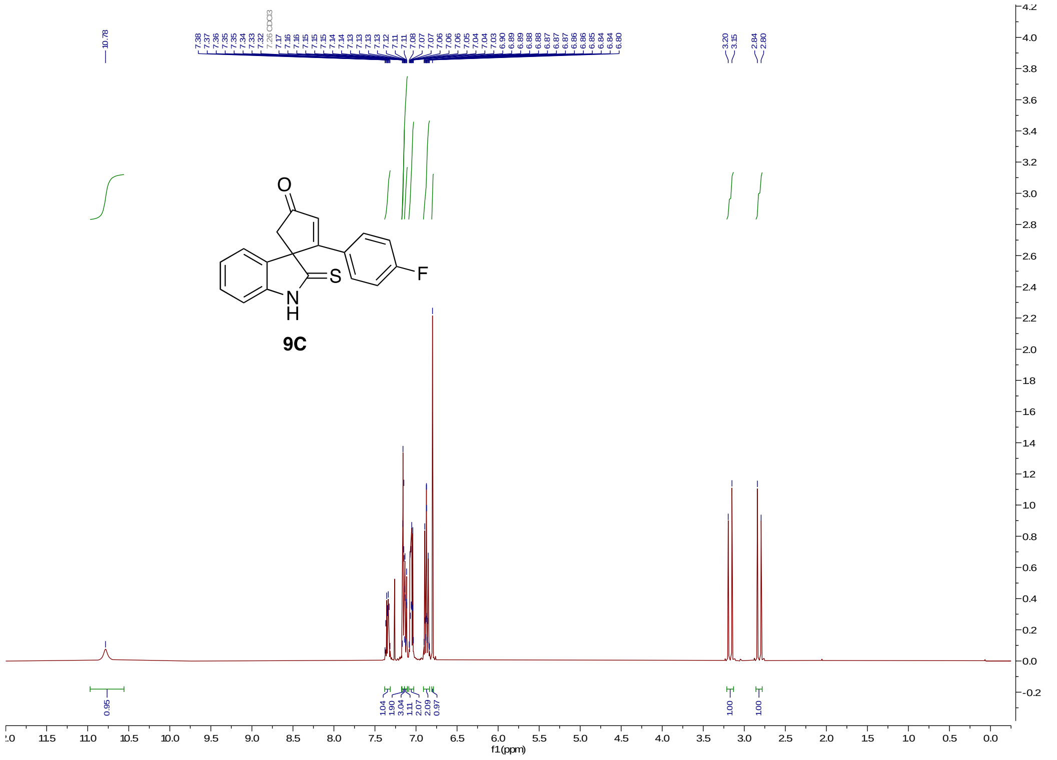

^{13}C NMR spectrum of **2-(4-Fluorophenyl)-2'-thioxospiro[cyclopentane-1,3'-indolin]-2-en-4-one (9c)** (100 MHz, $CDCl_3$)

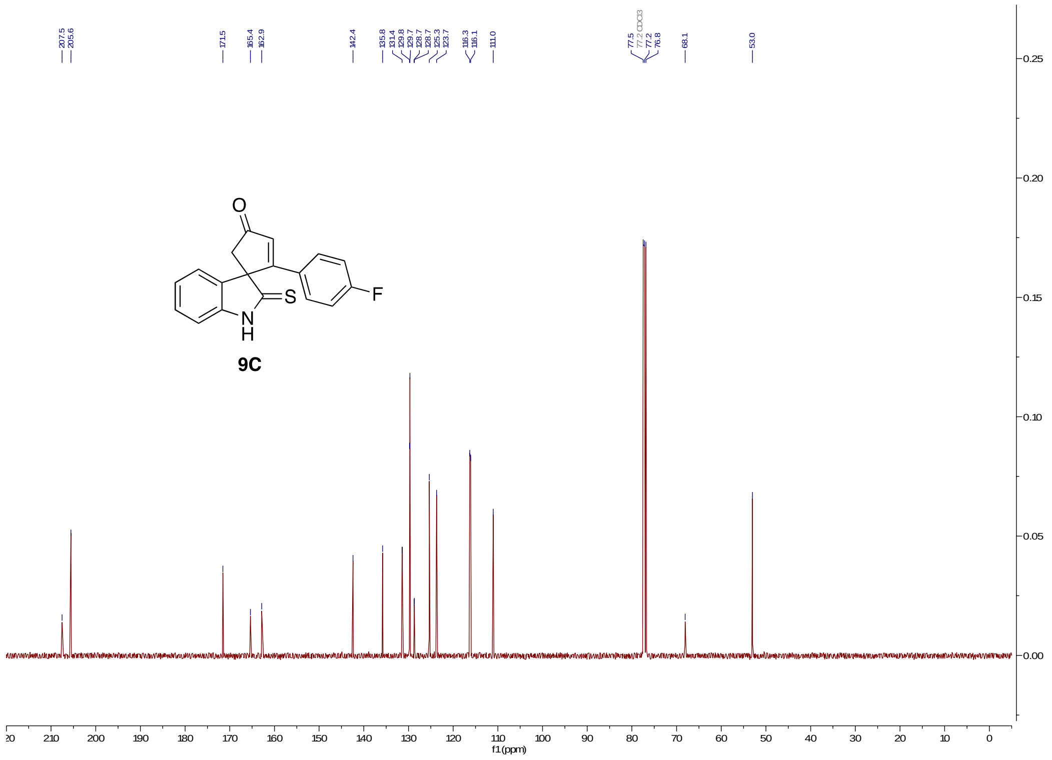

^{19}F NMR spectrum of **2-(4-Fluorophenyl)-2'-thioxospiro[cyclopentane-1,3'-indolin]-2-en-4-one (9c)** (376 MHz, $CDCl_3$)

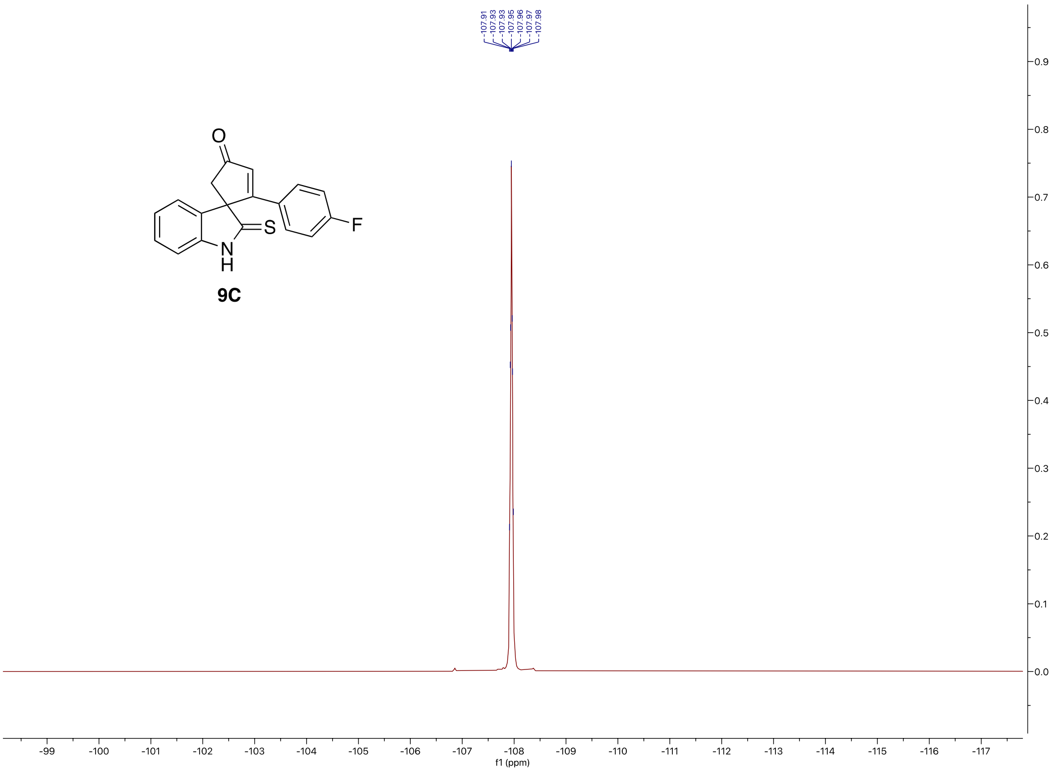

^{1}H NMR spectrum of **5'-Bromo-2-phenyl-2'-thioxospiro[cyclopentane-1,3'-indolin]-2-en-4-one (9d)** (400 MHz, DMSO-d_6)

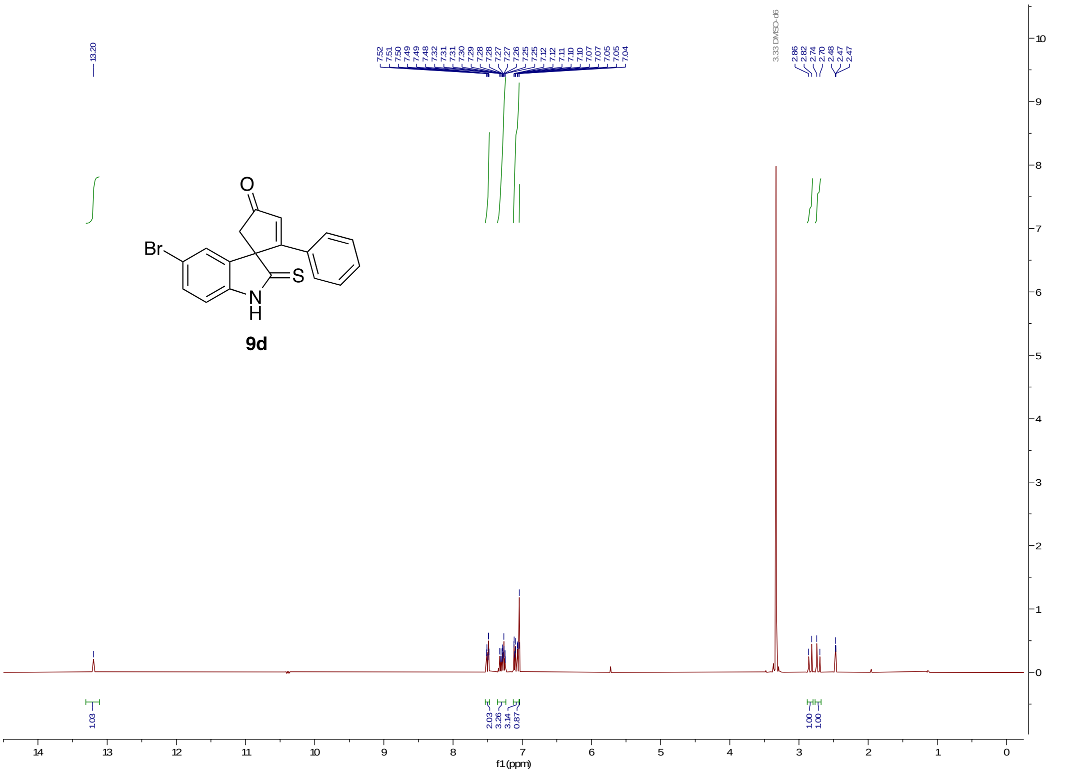

^{13}C NMR spectrum of **5'-Bromo-2-phenyl-2'-thioxospiro[cyclopentane-1,3'-indolin]-2-en-4-one (9d)** (100 MHz, DMSO-d_6)

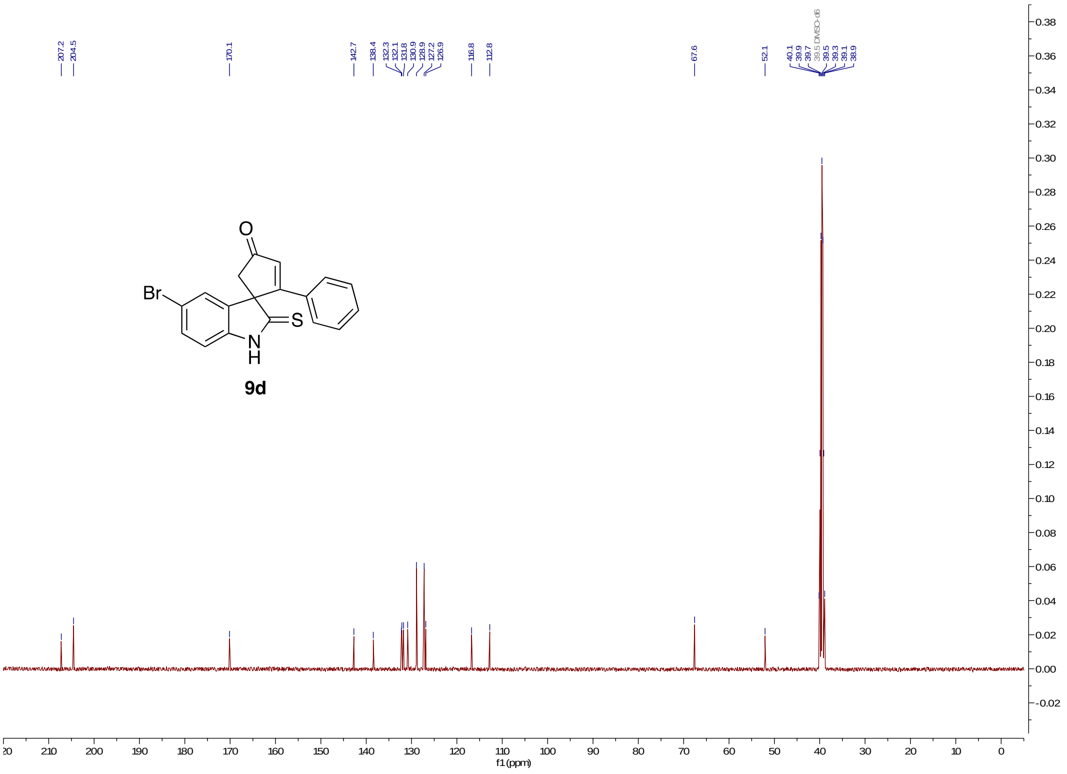

^{1}H NMR spectrum of **2-Phenyl-2'-(*p*-tolylthio)spiro[cyclopentane-1,3'-indol]-2-en-4-one (6a)** (400 MHz, $CDCl_3$)

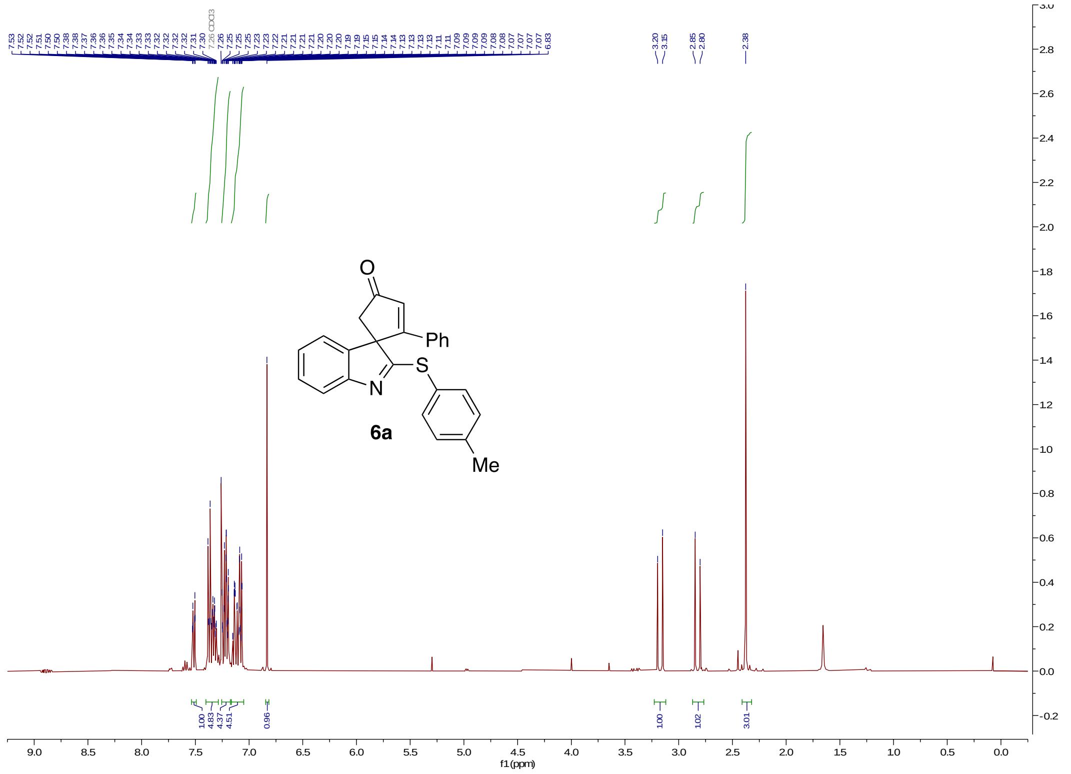

^{13}C NMR spectrum of **2-Phenyl-2'-(*p*-tolylthio)spiro[cyclopentane-1,3'-indol]-2-en-4-one (6a)** (100 MHz, $CDCl_3$)

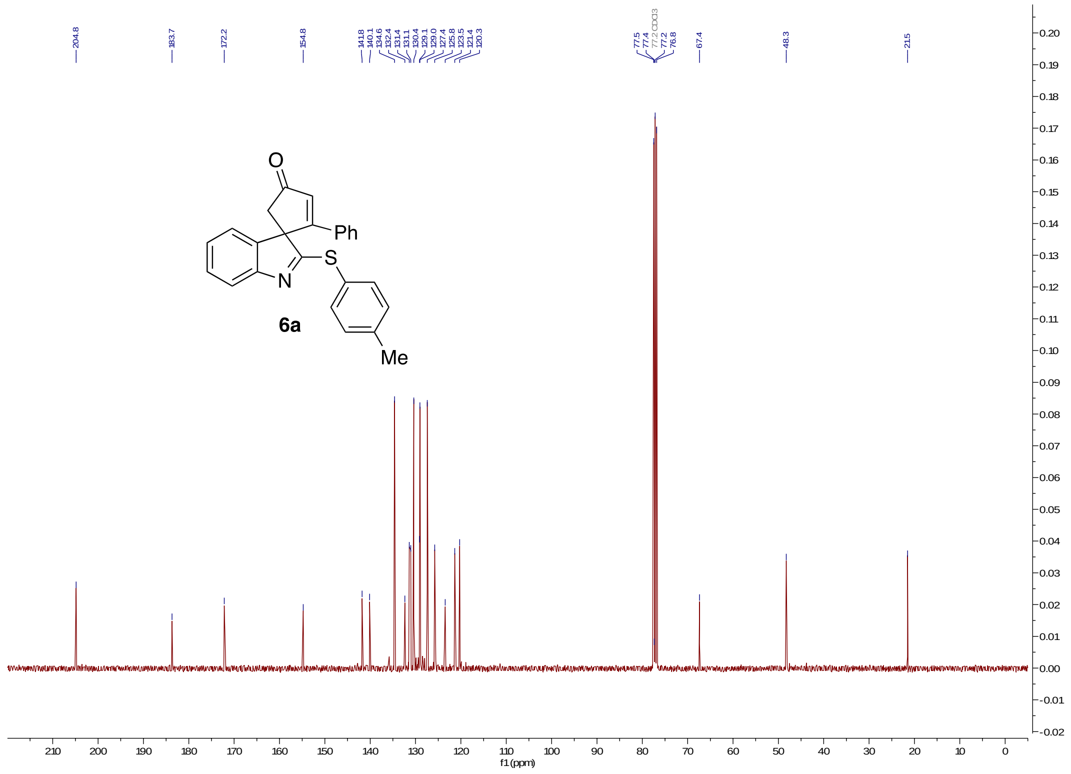

^{1}H NMR spectrum of **2-Phenyl-2'-(phenylthio)spiro[cyclopentane-1,3'-indol]-2-en-4-one (6a)** (400 MHz, $CDCl_3$)

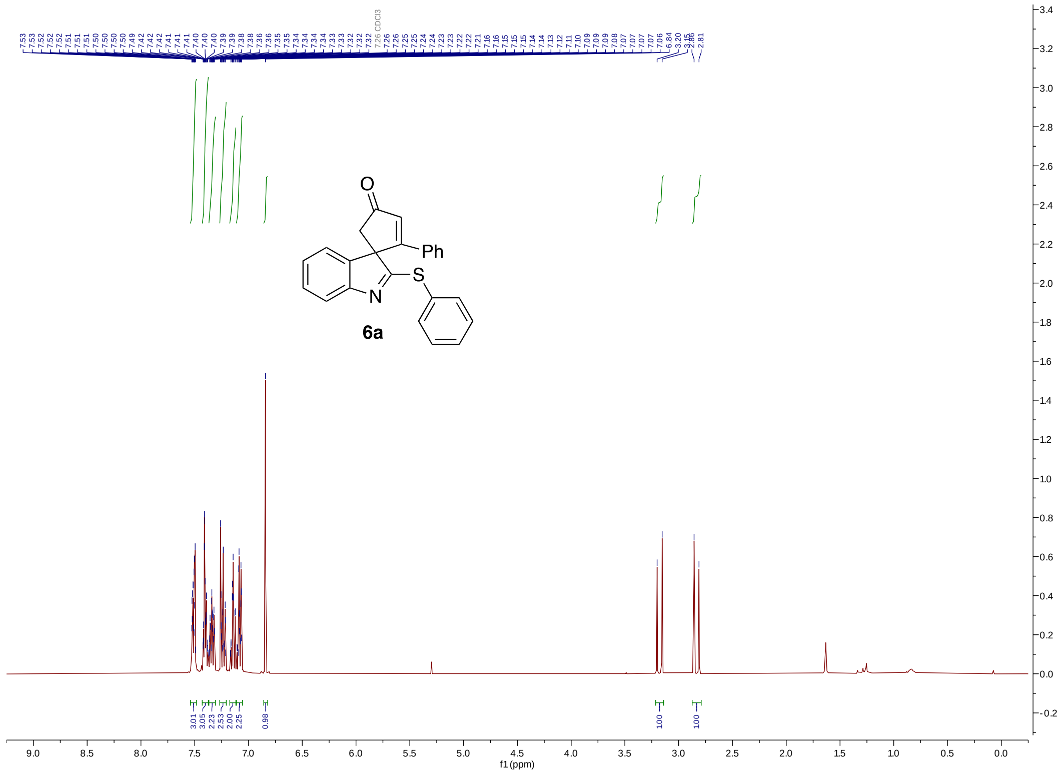

^{13}C NMR spectrum of **2-Phenyl-2'-(phenylthio)spiro[cyclopentane-1,3'-indol]-2-en-4-one (6a)** (100 MHz, $CDCl_3$)

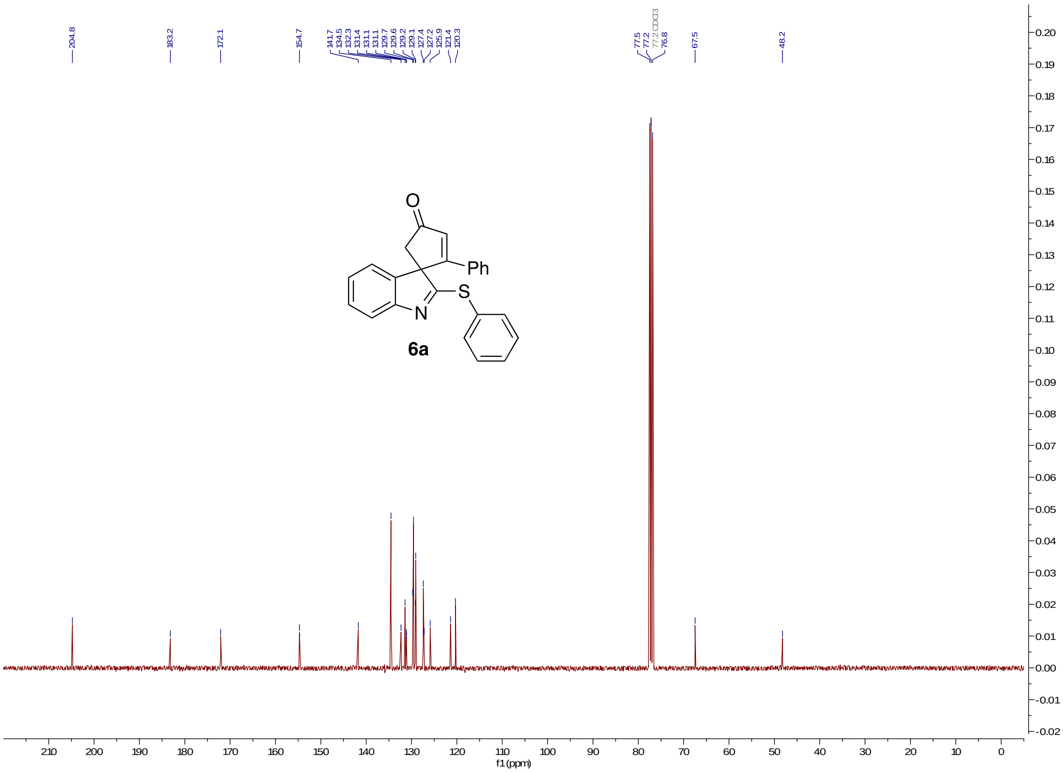

X-Ray Crystallography

Compound **4v** (CCDC 2054407) was confirmed by X-ray crystallography. Supplementary crystallography data can be downloaded from www.ccdc.cam.ac.uk/conts/retrieving.html.

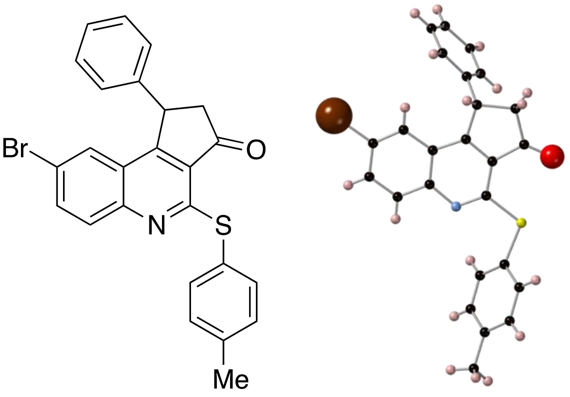

4v (CCDC 2054407)

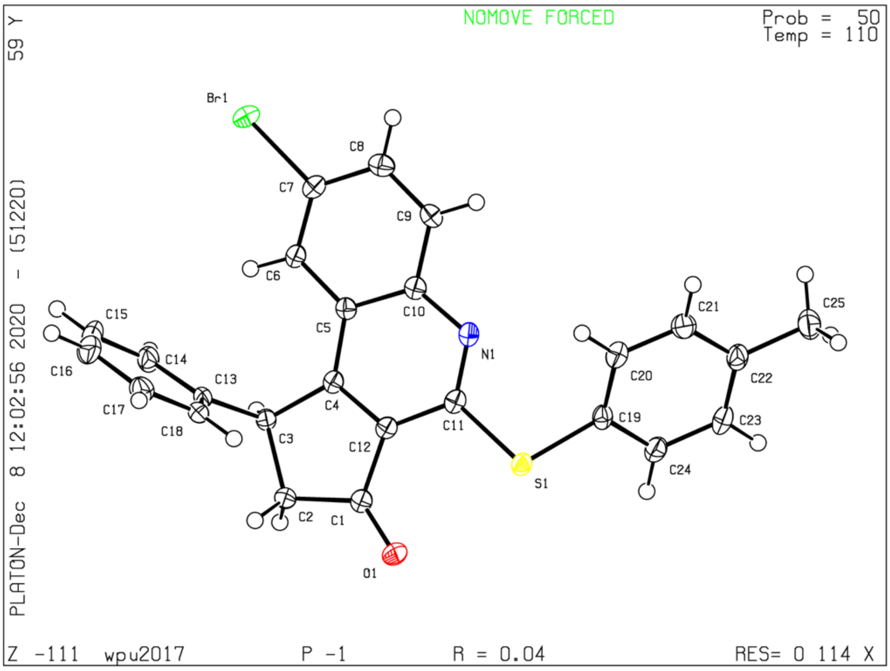

Experimental

A Single crystal of $C_{25}H_{18}BrNOS$ **4v** (CCDC 2054407) was prepared by slow evaporation of its solution in diethyl ether/hexane. A suitable crystal was selected and **[Oil on 200 um mount]** on a **SuperNova, Dual, Cu at home/near, Eos** diffractometer. The crystal was kept at 110.00(10) K during data collection. Using Olex2,[3] the structure was solved with the SHELXT[4] structure solution program using Intrinsic Phasing and refined with the SHELXL[5] refinement package using Least Squares minimisation.

Crystal data and structure refinement for 4v (CCDC 2054407).

Identification code	wpu2017
Empirical formula	$C_{25}H_{18}BrNOS$
Formula weight	460.37
Temperature/K	110.00(10)
Crystal system	triclinic
Space group	P-1
a/Å	7.7503(5)
b/Å	9.8144(8)
c/Å	14.4245(12)
α/°	108.074(7)
β/°	92.906(6)
γ/°	104.188(7)
Volume/Å^3	1001.63(14)
Z	2
ρ_{calc}g/cm^3	1.526
μ/mm^{-1}	2.173
F(000)	468.0
Crystal size/mm^3	0.263 × 0.174 × 0.127
Radiation	Mo Kα (λ = 0.71073)
2Θ range for data collection/°	6.594 to 58.188
Index ranges	$-10 \leq h \leq 10$, $-12 \leq k \leq 10$, $-16 \leq l \leq 19$
Reflections collected	8017
Independent reflections	4619 [R_{int} = 0.0232, R_{sigma} = 0.0455]
Data/restraints/parameters	4619/0/263
Goodness-of-fit on F^2	1.102
Final R indexes [I>=2σ (I)]	R_1 = 0.0440, wR_2 = 0.0990
Final R indexes [all data]	R_1 = 0.0536, wR_2 = 0.1039
Largest diff. peak/hole / e Å^{-3}	2.38/-0.66

References

1. J. T. Liddon, A. K. Clarke, R. J. Taylor and W. P. Unsworth, Org. Lett., 2016, 18, 6328-6331.
2. J. T. Liddon, M. J. James, A. K. Clarke, P. O'Brien, R. J. K. Taylor and W. P. Unsworth, Chem. Eur. J., 2016, 22, 8777-8780.
3. Dolomanov, O.V., Bourhis, L.J., Gildea, R.J, Howard, J.A.K. & Puschmann, H. (2009), J. Appl. Cryst. 42, 339-341.
4. Sheldrick, G.M. (2015). Acta Cryst. A71, 3-8.
5. Sheldrick, G.M. (2015). Acta Cryst. C71, 3-8.
